# Bonding Features and Magnetic Ordering in Thiolate‐Bridged Copper‐Nickel Clusters Synthesized at Elevated Temperature

**DOI:** 10.1002/smll.202506920

**Published:** 2025-08-08

**Authors:** Arijit Jana, Yaofeng Wang, Lukas Guggolz, Yaorong Chen, Franziska Ganslmaier, Bastian Weinert, Mario Ruben, Stefanie Dehnen

**Affiliations:** ^1^ Institute of Nanotechnology Karlsruhe Institute of Technology (INT) Kaiserstraße 12 76131 Karlsruhe Germany; ^2^ Institute of Quantum Materials and Technologies (IQMT) Karlsruhe Institute of Technology Kaiserstraße 12 76131 Karlsruhe Germany; ^3^ Centre Européen de Sciences Quantiques Institut de Science et d'Ingénierie Supramoléculaires (ISIS, UMR 7006) CNRS‐Université de Strasbourg 8 allée Gaspard Monge BP Strasbourg Cedex 70028 67083 France

**Keywords:** heterometallic nickel‐based clusters, magnetism, optical properties, *pseudo*‐polymorphism, density functional theory calculations

## Abstract

Atomically‐precise heterometallic nickel‐based clusters are an emerging class of functional nanomaterials with intriguing optical and magnetic properties. However, synthetic challenges restrict their exploration in comparison to heterometallic coinage metal‐based nanoclusters. This study presents a single‐step synthesis of the two new thiolate‐bridged copper‐nickel cluster compounds [Cu_2_Ni_6_S_3_(MCP)_6_] (**1**) and [Cu_2_Ni_6_(MCP)_12_I_2_] (**2**) (MCPH = 2‐mercaptopyridine; MCP = deprotonated 2‐mercaptopyridine) at an elevated temperature. Single‐crystal X‐ray diffraction reveals that **1** is composed of polymeric strands of linked cluster units. Each of the units exhibit a bicapped trigonal prismatic {Cu_2_Ni_6_} core that is surrounded by three capping sulfide and six MCP ligands. Cluster **2** features individual clusters, each bearing a hexagonal bipyramidal {Cu_2_Ni_6_} core with twelve MCP units as well as two iodide ions as additional ligands. In spite of the different aggregation modes, both of these clusters exhibit molecule‐like characteristic multiband optical absorption features. Temperature‐dependent magnetic susceptibility measurements for **1** revealed dual antiferromagnetic and ferromagnetic coupling among six Ni(II) centers with an *S* = 2 ground state, while **2** exhibits strong ferromagnetic coupling, whereby the susceptibility increases with decreasing temperature to an *S* = 4 ground state. This study is an example of solvothermal synthesis of related ligand‐supported copper‐nickel cluster compounds with structure‐specific optical and magnetic properties.

## Graphical abstract

Two thiolate‐decorated bimtallic Cu─Ni clusters formed under solvothermal synthesis conditions. [Cu_2_Ni_6_S_3_(MCP)_6_] (MPCH = 2‐mercaptopyridine; MCP = deprotonated 2‐mercaptopyridine) features a bicapped trigonal prismatic {Cu_2_Ni_6_} core, while [Cu_2_Ni_6_(MCP)_12_I_2_] possesses a hexagonal bipyramidal {Cu_2_Ni_6_} core. The structural differences are also reflected in a different magnetic coupling behavior of the Ni ions.

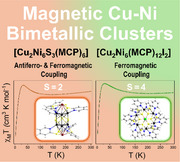

## 1 Introduction

Atomically precise nanoclusters composed of metal atoms arranged in well‐defined geometries and stabilized by ligands form a unique class of functional nanomaterials, characterized by diverse structural motifs and distinctive physicochemical properties.^[^ ^1^, ^2^, ^3^ ^]^ These ultra‐small particles with a typical size of below 3 nm have characteristic absorption/emission properties and nonlinear optical behavior, as well as interesting electrochemical properties.^[^ ^4^, ^5^, ^6^, ^7^, ^8^ ^]^ These properties make them suitable candidates to use in the fields of optoelectronic or imaging devices, for the photo‐ and electrocatalytic reduction of carbon dioxide or nitrogen, electrocatalytic oxidation of water, organic coupling reactions, as well as in the electrochemical sensing of various analytes.^[^ ^9^, ^10^, ^11^, ^12^, ^13^, ^14^, ^15^, ^16^ ^]^ In the context of ligand stabilized metal clusters, a large number of atomically precise clusters with coinage metal atoms (i.e., gold, silver and copper) were previously synthesized and characterized.^[^ ^1^, ^2^, ^3^, ^17^, ^18^, ^19^, ^20^, ^21^, ^22^ ^]^ In contrast to this, sensitivity to air and moisture, unfavorable reduction behavior of precursor metal ions, and the lack of strong intermetallic covalent bonding so far hampered the accessibility of similar lighter fourth period metal clusters, particularly Ni analogues.^[^ ^23^, ^24^, ^25^ ^]^ Heterometallic nickel‐based clusters constitute a subclass, in which appropriate heterometal atoms are added to form a mixed‐metal cluster core. There are only a few reported cases of bimetallic clusters where heterometal atoms such as gold, platinum, tin, iridium, antimony and tungsten atoms have been successfully integrated into nickel clusters.^[^ ^26^, ^27^, ^28^, ^29^, ^30^, ^31^, ^32^ ^]^ To the best of our knowledge, there is no report of ligand protected bimetallic clusters comprised of copper and nickel. Hence, the development of robust heterometallic copper‐nickel clusters will be a significant addition to the family of heterometallic nickel‐based clusters.

Surface‐protecting ligands play a crucial role for the stabilization of nickel‐based clusters. The majority of these clusters are protected by phosphines,^[^ ^33^, ^34^, ^35^, ^36^ ^]^ alcohols,^[^ ^37^ ^]^ oximates,^[^ ^38^, ^39^ ^]^ calixarenes,^[^ ^40^, ^41^, ^42^ ^]^ Schiff bases,^[^ ^43^, ^44^ ^]^ carbonyl^[^ ^45^, ^46^ ^]^ and polyoxometalates^[^ ^47^, ^48^ ^]^ and some of them are stabilized by thiolated ligands.^[^ ^49^ ^]^ Notably, thiolated nickel clusters are typically synthesized at temperatures ranging from 0 °C to room temperature through the chemical reduction of nickel thiolate complexes, often using reducing agents like sodium borohydride.^[^ ^50^, ^51^ ^]^ Structural investigations using single‐crystal X‐ray diffraction (SC‐XRD) classify nickel clusters into two groups: oligocyclic and non‐oligocyclic. Oligocyclic nickel and heterometallic nickel‐based clusters are composed of ring‐shaped, oligonuclear, or tiara‐like nickel‐chalcogenide cores, which are protected by surface ligands.^[^ ^52^, ^53^, ^54^ ^]^ Additionally, a handful of non‐oligocyclic nickel clusters have been reported and are noted for their Ni─Ni bonds, e.g., [(^i^Pr_3_P)Ni]_4_H_4_L (L = NCH_2_Ph, (BH)_2_, (Cl)_2_ O; Ni─Ni 2.3467–2.6064 Å, [Ni_30_S_16_(PEt_3_)_11_], or [Ni_26_S_14_(PEt_3_)_10_] (Ni─Ni 2.4825–2.7964), or at least weak interactions in [Ni_8_(Xpz)_12_(OH)_6_]^2−^ (X═Cl Br, I; Ni─Ni 2.9580–3.0009).^[^ ^55^, ^56^, ^57^, ^58^ ^]^ The delocalized d‐electrons play a crucial role in stabilizing these clusters. In this context, efforts continue to synthesize heterometallic nickel‐based clusters with novel structures, different sizes, and different ligand envelopes.

Nickel and heterometallic nickel‐based clusters, although less frequently discussed, exhibit unique structure‐specific magnetic properties due to the presence of unpaired electrons present in the Ni(II) centers. Furthermore, their magnetic properties hold significant potential for advanced technologies, including single‐molecule magnets, quantum bits for quantum computing, and magnetic data storage.^[^ ^59^, ^60^, ^61^, ^62^, ^63^ ^]^ In this context, extensively studied coinage metal clusters predominantly display either weak paramagnetic behavior or diamagnetism, as determined by electron paramagnetic resonance measurements.^[^ ^64^, ^65^ ^]^ Unlike bulk ferromagnetic nickel metal, nickel and heterometallic nickel‐based clusters exhibit diverse types of paramagnetic, ferromagnetic, and superparamagnetic properties due to the presence of unpaired electrons in their open‐shell ground electronic states.^[^ ^66^, ^67^, ^68^, ^69^ ^]^ Magnetic properties also depend on the electronic coupling of nearby metal atoms, intermetallic bonding, charge states and the arrangement of ligands, as demonstrated for nickel clusters with 15 atoms, in which the magnetic moments vary depending on the nature of intermetallic bonding.^[^ ^70^ ^]^ The mixing of 3d and 4s atomic orbitals within the inner framework of these clusters also influences its magnetic properties by disrupting spin symmetry and exchange coupling between the nearby Ni centers.^[^ ^71^ ^]^ To investigate the correlation between molecular structure and magnetic properties, we aimed to synthesize examples of a so far unexplored class of thiolated copper‐nickel clusters.

In this study, two copper‐nickel cluster compounds, [Cu_2_Ni_6_S_3_(MCP)_6_] (**1**) and [Cu_2_Ni_6_(MCP)_12_I_2_] (**2**), were obtained in a solvothermal approach. The cluster in **1** features a {Ni_6_} core and exhibits a 1D extension via Cu─Cu and Cu─S linkages. The cluster in **2**, in contrast, forms non‐linked clusters based on a crown‐like skeleton, primarily protected by 2‐mercaptopyridine ligands. Besides X‐ray diffraction analyses, we characterized both compounds using high‐resolution mass spectrometry, quantum chemical calculations and various spectroscopic techniques. Temperature‐dependent magnetic measurements revealed an antiferromagnetic and ferromagnetic coupling behavior for **1** and ferromagnetic coupling behavior for **2**, indicating that the structural differences observed for these related clusters with similar chemical compositions are instrumental for their characteristic electronic and magnetic properties.

## 2 Results and Discussion

**Scheme 1:**
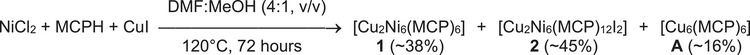
Non‐stoichiometric reaction scheme for the solvothermal synthesis of compounds **1**, **2** and **A**.

Compounds **1** and **2** were synthesized by a solvothermal reaction of nickel(II) chloride, copper(I) iodide and 2‐mercaptopyridine (MCPH) in a solvent mixture (4:1, v/v) of *N*,*N*‐dimethylformamide (DMF) and methanol (MeOH), see **Scheme** **1**.

**Figure 1:**
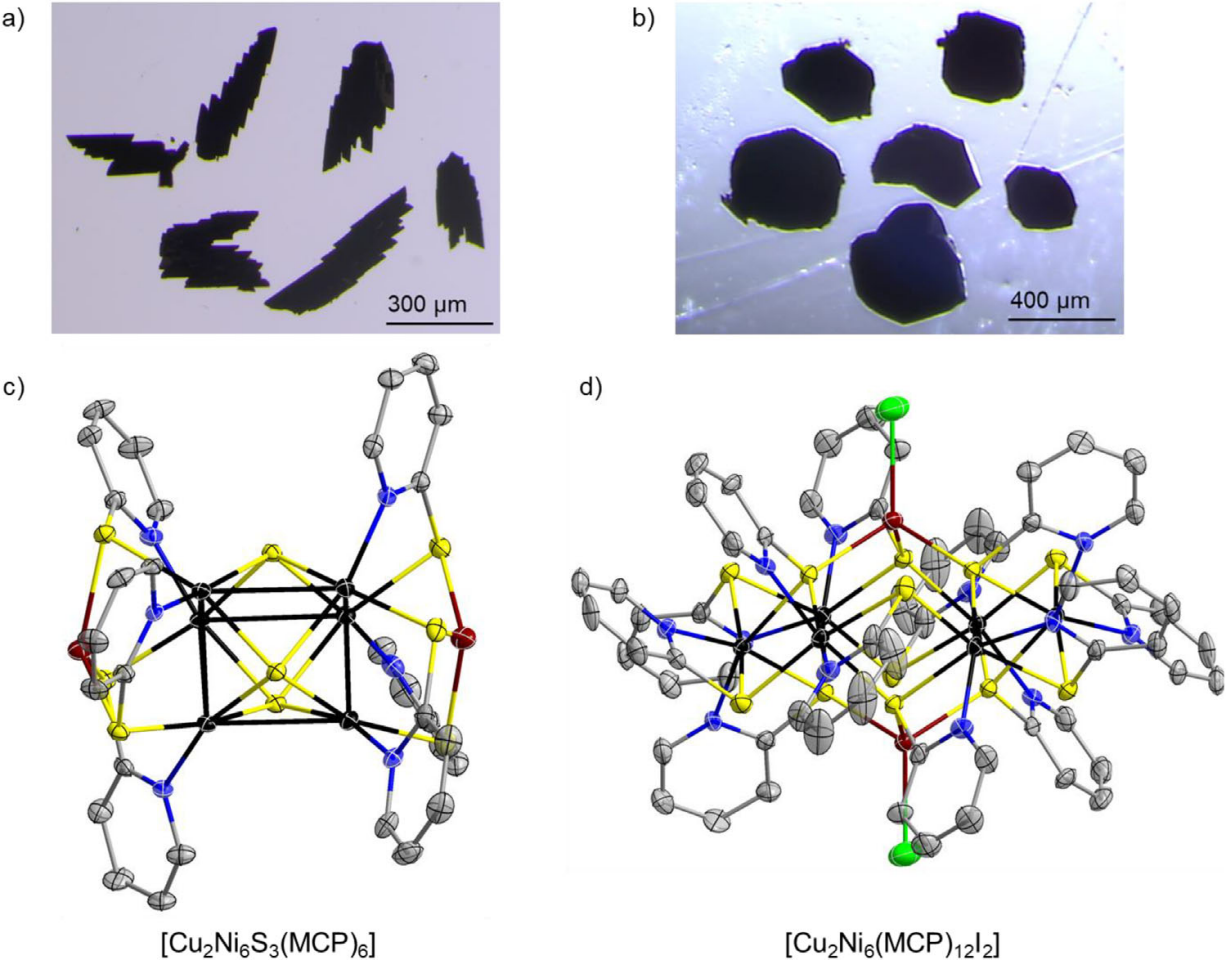
Optical photograph of single crystals of a) **1** and b) **2a**. Illustration of the molecular structures of clusters c) [Cu_2_Ni_6_S_3_(MCP)_6_] in **1** and d) [Cu_2_Ni_6_(MCP)_12_I_2_] in **2**. Thermal ellipsoids are drawn at the 50% probability level. Atom color code: black = Ni, dark red = Cu, yellow = S, blue = N, grey = C, green = I; H atoms are omitted for clarity.

After 72 h of solvothermal treatment in a vacuum‐sealed pyrex ampule at 120 °C, **1** crystallizes as black sheet‐like single crystals, while **2** forms dark green polyhedral and green cuboidal crystals (**Figure** **1**a,b). Further details are provided in the experimental section. Additionally, we obtained orange rod‐shaped crystals, resembling the previously reported cluster compound [Cu_6_(MCP)_6_] (**A**, Figure S1, Supporting Information), according to single‐crystal X‐ray diffraction (SC‐XRD) analyses.^[^ ^72^, ^73^, ^74^ ^]^ A lack of copper(I) iodide under the same reaction conditions leads to a substantial quantity of hair‐like microcrystals (Figure S2, Supporting Information), the identity of which could not be determined by means of SC‐XRD. The powder X‐ray diffraction (P‐XRD) patterns of these aggregates do not match those of **1** and **2**, indicating the formation of a yet unknown Ni‐MCP compound (Figure S3, Supporting Information). Another control reaction without using nickel(II) chloride resulted in quantitative (yield 95%) synthesis of a crystalline product. The obtained P‐XRD diffraction pattern of the product matches well with the simulated pattern of [Cu_6_(MCP)_6_] (**A**, Figure S4, Supporting Information).

SC‐XRD studies reveal that compound **1** crystallizes in the orthorhombic crystal system, space group type *Pbcn*, with the cell parameters *a*  =  19.2770(18) Å, *b*  =  19.490(2) Å, *c*  =  21.376(3) Å, *V*  =  8031.2(15) Å^3^ and *Z*  =  1 (Table S1, Supporting Information). The molecular structure of one cluster unit is shown in Figure 1c.

**Figure 2:**
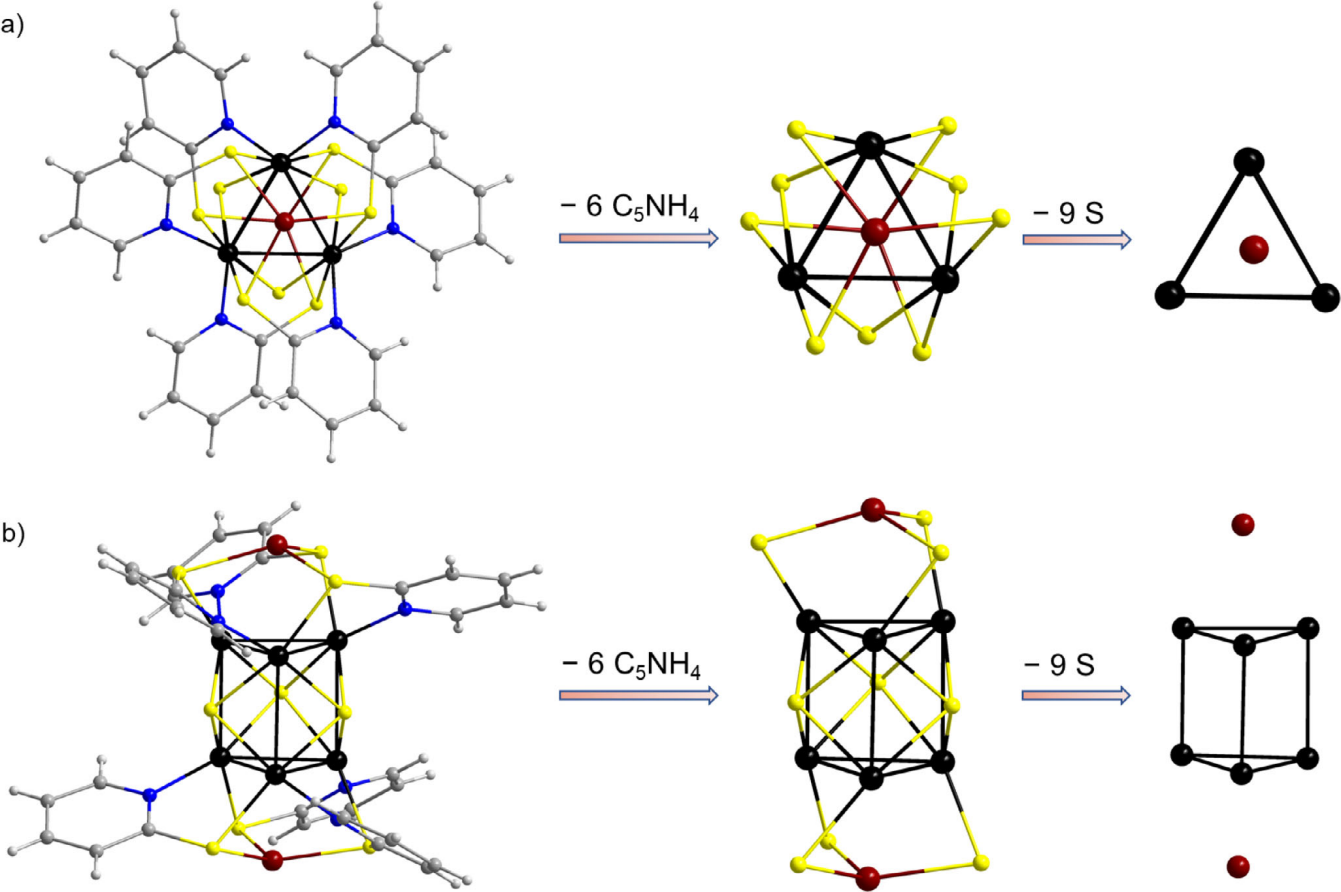
Molecular structure of the [Cu_2_Ni_6_S_3_(MCP)_6_] cluster units in **1** in different orientations: a) front view and b) side view, both with additional representations upon removing, first, the six pyridine groups (center) and, second, all sulfur atoms (right). Atom color code: black = Ni, dark red = Cu, yellow = S, blue = N, grey = C, white = H.

The [Cu_2_Ni_6_S_3_(MCP)_6_] cluster in **1** features a {Cu_2_Ni_6_} core composed of a trigonal prismatic {Ni_6_} inner framework (**Figure** **2**). Each triangular {Ni_3_} face of the prism is capped by a Cu atom, while each rectangular {Ni_4_} face is capped by a *µ*_4_‐S ligand. The MCP molecules act as bidentate ligands, with the N donor atoms coordinating one nickel atom and the S donor atoms acting as *µ*_2_‐bridges between the Cu and the Ni atoms. This results in a tripodal coordination behavior of the MCP ligands, which at the same time induces a helical orientation of the three pyridine ligands on each side of the cluster (Figure S5, Supporting Information). Direct Ni─Ni contacts vary between 2.691(14) and 2.860(14) Å, which are well within the range of distances reported for other clusters exhibiting bonding Ni─Ni interactions.^[^ ^55^, ^56^, ^57^ ^]^ The (non‐bonding) distance between the capping Cu atoms is 7.511(18) Å. The *µ*_4_‐S─Ni distances for the capping sulfide atoms amount to 2.179(18)–2.233(18) Å, while the *µ*_2_‐S─Ni and *µ*_2_‐S─Cu bond lengths fall within the range of 2.240(20)–2.306(20) Å and 2.295(20)–2.382(20) Å, respectively. Ni─N distances are 1.946(62)–1.969(59) Å. As Ni and Cu cannot be discriminated by means of standard X‐ray diffraction experiments, the metal‐atom composition of the cluster was confirmed by micro‐X‐ray fluorescence spectroscopy (*µ*‐XRF) performed on the corresponding crystals. The *µ*‐XRF spectra of three individual single crystals consistently show a Cu:Ni ratio of 1:3, which confirms the presence of the {Cu_2_Ni_6_} core per cluster unit (Figure S6, Supporting Information). The fact that counterions could not be localized on the difference Fourier map and the absence of chloride and iodide signals in the *µ*‐XRF analysis suggest that compound **1** represents a neutral compound “1D‐[Cu_2_Ni_6_S_3_(MCP)_6_]_n_”. This instantly raises questions about the oxidation state of the Cu and Ni atoms, which will be addressed by further analyses below.

Compound **1** exhibits a chain‐like arrangement of the cluster units along the crystallographic *b* axis, where two cluster units are connected through Cu─Cu (2.685(16) Å) and Cu─S (2.685(21) Å) bonds (Figures S7 and S8, Supporting Information). In the extended solid‐state structure, these chains assemble into a lamellar packing along the crystallographic *a* and *c* axes, stabilized by C─H···H─C (2.330(15) Å), C─H···S (2.861(22) Å), C─H···*π* (2.676(20) Å), and *π*···*π* (3.310(15) Å) short contact interactions (Figures S8 and S9, Supporting Information).

Structural *pseudo*‐polymorphism was observed for compound **2** (**2a**,**b**), which simultaneously crystallized as dark green polyhedra (**2a**, triclinic space group type *P* $\bar{1}$ with the cell parameters *a* = 12.3680(5) Å, *b* = 13.3888(5) Å, *c* = 13.7219(4) Å, *α* = 115.397(2)°, *β* = 97.655(3)°, *γ* = 104.303(3)°, *V* = 1913.34(13) Å^3^ and *Z* = 1) and greenish cuboidal crystals (**2b**, monoclinic space group type *P*2_1_/*c* with the cell parameters *a* = 13.8178(14) Å, *b* = 12.4690(16) Å, *c* = 24.384(3) Å, *β* = 98.714(9)°, *V* = 4152.7(8) Å^3^ and *Z* = 2). Crystallographic details and structural refinements are summarized in Tables S2 and S3 and Figure S10 (Supporting Information). Both compounds differ by the presence of two crystal solvent (DMF) molecules per formular unit of compound **2b**, but feature the same cluster motif as compound **2a**.

**Figure 3:**
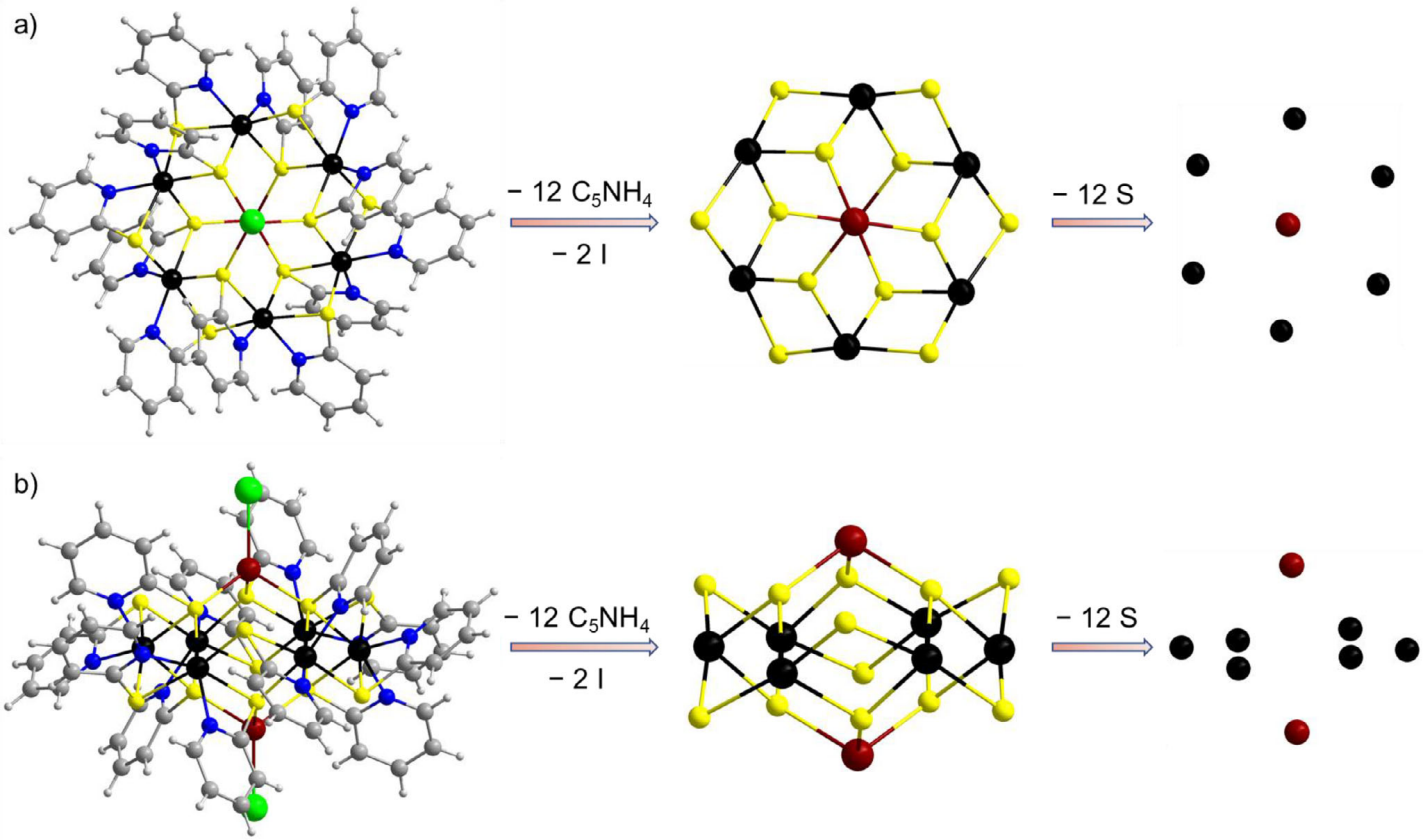
Molecular structure of the [Cu_2_Ni_6_(MCP)_12_I_2_] cluster in **2** in different orientations: a) front view and b) side view, with additional representation by removing, first, all pyridine groups as well as the iodine atoms (center) and, second, all sulfur atoms (right). Atom color code: black = Ni, dark red = Cu, yellow = S, blue = N, grey = C, green = I, white = H.

The clusters in compound **2** are composed of six nickel and two copper atoms that are primarily coordinated by twelve MCP ligands and two iodine atoms (**Figure** **3**). The {Cu_2_Ni_6_} architecture can be described as a non‐bonded distorted hexagonal bipyramid, with six Ni atoms forming the hexagonal base with a very flat chair‐like conformation and the two Cu atoms occupying the axial capping positions. The Ni···Ni distances within the hexagons range from 3.439(18) to 3.518(13) Å (**2a**) and from 3.445(12) to 3.482(12) Å (**2b**) while the Cu···Cu distances are between 4.950(19) Å and 5.180(14) Å for both **2a**,**b** (Figure S11, Supporting Information), indicating no bonding metal‐metal interactions. The nickel atoms are connected via two *µ*_2_‐S ligands (Ni─S bond lengths: 2.402(31)–2.553(29) Å for **2a**, 2.403(20)–2.557(29) Å for **2b**; Ni─S─Ni angles: 87.833(89)–92.149(75)° for **2a** and 88.287(62)–90.672(57)° for **2b**). The Ni/S substructure alone can best be described as a crown‐like ring with a {Ni_6_S_12_} skeleton, which resembles various oligocyclic tiara‐ or crown‐like nickel clusters (Figure S12, Supporting Information).^[^ ^50^, ^51^, ^52^, ^53^, ^54^ ^]^ Two Cu atoms are bonded to the top and bottom of the {Ni_6_S_12_} core via Cu─S bonds featuring distances of 2.334(24)–2.346(26) Å (**2a**) and 2.350(18)–2.370(18) Å (**2b**). The overall {Cu_2_Ni_6_S_12_} motif displays a hexagonal symmetry viewed from top (Figure 3a) and features an oblate shape in side view (Figure 3b).

Each iodine atom binds exclusively to a capping Cu atom (Cu─I bond lengths of 2.489(16) Å for **2a** and 2.555(11) Å for **2b**, respectively). For Cu and Ni being indistinguishable by standard X‐ray diffraction experiments, *µ*‐XRF analyses were carried out to assess the metal atom composition in these clusters, as shown in Figures S13 and S14 (Supporting Information) for **2a**,**b**, respectively. Analysis of three independently crystals from each sample revealed a Cu:Ni ratio of 1:3, further confirming the presence of two Cu and six Ni atoms in the clusters. Co‐crystallized DMF solvent molecules interact with the **2b** cluster through C─H···H─C (2.332(15) Å), I···H─C (3.162(6) Å) and C─H···O (2.681(10) Å) (shown in Figure S15, Supporting Information). In **2a** the [Cu_2_Ni_6_(MCP)_12_I_2_] clusters are packed layer‐like along the crystallographic axes a and b, whereas in **2b** helical packing along the b axis dominates the structure (Figures S16 and S17, Supporting Information). A few recent studies have also reported similar helical‐like packing motifs in metal nanoclusters.^[^ ^74^, ^75^, ^76^ ^]^

To get deeper insight into the molecular structures and bonding situations of **1** and **2**, we performed quantum chemical studies, employing density functional theory (DFT) methods. A full summary of the used methods and procedures is given in the Supporting Information.

Geometry optimization of an isolated cluster unit of **1** showed good agreement between calculated and experimental structural parameters for the central {Ni_6_S_3_} part, but not for the two apical Cu atoms with the surrounding MCP ligands: treating **1** as a monomer leads to an inward movement of the Cu atoms and to calculated Cu···Ni distances, which were up to 0.2 Å shorter than the experimental ones, which indicated the necessity of taking the 1D extension of the cluster units into account. The geometry optimization of a dimeric subunit, however, fit the experimental values in this region well, thus indirectly confirming both the polymeric nature of **1** and the overall neutral charge. A comparison of experimental and calculated bond lengths is given in Table S4 (Supporting Information). The inspection of localized molecular orbitals (LMOs; Figure S19, Supporting Information) showed no signs of direct Cu─Ni bonding, hence the Cu atoms are held in place solely by the MCP ligands. Within the central {Ni_6_S_3_} subunit, we find regular 2‐center 2‐electron (2c2e) Ni─S bonds as well as 3‐center 2‐electron (3c2e) Ni─S─Ni bonds, indicative of some bonding activity also between the metal atoms. The respective monomer units interact via multicenter bonds within the connecting {Cu_2_S_2_} four‐rings.

Optimizing the molecular structure of **2** again showed a good agreement with the experimental structural data. It has to be noted, though, that in the isolated cluster units, the six outer S atoms rotate slightly about the *pseudo*‐sixfold (more closely: threefold) axis in the horizontal plane, leading to more distinct differences between shorter and longer Ni─S bonds as compared to the experimental values (Table S6, Supporting Information). We attribute this observation to the movement of the MCP ligands during the calculation, which is suppressed in the crystal structure due to stabilizing secondary interactions with neighboring molecules. On average however, the bond lengths fit the experimental data, which points to a match of the total electron count and thus correct charges. In the absence of any “naked” S atoms (as in **1**) the bonding situation is less diverse, with all contacts being regular 2c2e Ni─S or Cu─S bonds within the inorganic cluster core of **2** (Figure S21, Supporting Information).

**Figure 4:**
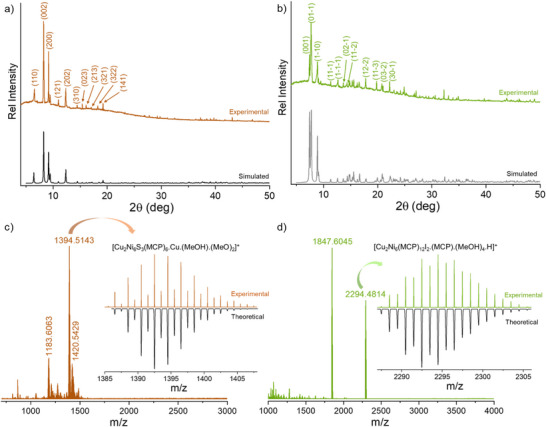
Comparison of the experimental P‐XRD patterns of manually selected crystals with the simulated patterns obtained from SC‐XRD for a) **1** and b) **2a**. The full range positive ion mode ESI‐MS spectrum of c) **1** and d) **2a** dissolved in a mixture of DMF and MeOH. Inset shows the high‐resolution spectra and the isotopic distribution of the experimental spectrum in comparison with the simulated pattern of [Cu_2_Ni_6_S_3_(MCP)_6_.Cu.(MeOH).(MeO)_2_]^+^ and [Cu_2_Ni_6_(MCP)_12_I_2_.(MCP).(MeOH)_4_.H]^+^, respectively.

Crystals of both compounds were manually selected at ambient conditions using stainless steel microneedles for further characterization, including P‐XRD, mass spectrometry, infrared (IR), and Raman spectroscopy. Figure S22 (Supporting Information) displays optical microscopic images of the respective crystals after manual separation. The experimental P‐XRD pattern of **1** aligns closely with the simulated pattern derived from the SC‐XRD data (**Figure** **4**a). The primary reflections at lower 2*θ* angles at 6.44°, 8.26°, 9.16°, 10.98°, and 12.35° correspond to the (110), (002), (200), (121), and (202) lattice planes, respectively. The experimental P‐XRD pattern of **2a** also matches well with the simulated pattern (Figure 4b). Slight variations in intensity and peak splitting of the diffraction peaks can be attributed to the temperature difference between the SC‐XRD (measured at 150 K) and the P‐XRD (measured at 298 K) experiments, and to the fact that at least the platelet‐shaped crystals on the flatbed sample carrier align themselves in a preferred direction due to their shape. For hand‐selected crystals out of such a mixture, this experiment confirms that the phase consists of the respective clusters.

The molecular composition of the clusters was further confirmed through high‐resolution electrospray‐ionization mass spectrometry (ESI‐MS) measurements – as another important tool in light of the indistinguishability of Cu and Ni with standard X‐ray diffraction techniques. The ESI‐MS spectrum in positive ion mode of **1**, freshly dissolved in a mixture of DMF and MeOH (3:1, v/v), exhibits a prominent pattern around *m*/*z* 1394.5143 (Figure 4c). A peak‐to‐peak separation of Δ(*m*/*z*) = 1 suggests the detected species to be a monocation. The mass of the species matches with the molecular composition of the cluster, [Cu_2_Ni_6_S_3_(MCP)_6_·Cu·(MeOH)·(MeO)_2_]^+^, hence corresponding to the cluster [Cu_2_Ni_6_S_3_(MCP)_6_] in **1**, upon attachment of one Cu atom, two (MeO)^−^ ions and one MeOH molecule and concomitant oxidation under ESI‐MS conditions (inset of Figure 4c). In addition to this main peak, two minor peaks referring to monocations were detected at m/z 1183.6063 and 1420.5429. These peaks were assigned to the species [Ni_6_S_3_(MCP)_6_·(DMF)·H]^+^ and [Cu_2_Ni_6_S_3_(MCP)_6_·(DMF)·(MCP)]^+^, respectively (Figure S23, Supporting Information). For compound **2** (again recorded from a solution in a 3:1 DMF:MeOH mixture), the ESI‐MS spectrum in positive ion mode shows two patterns with high intensity at *m*/*z* 2294.4814 and 1847.6045. The peak at *m*/*z* 2294.4814 was attributed to the species [Cu_2_Ni_6_(MCP)_12_I_2_·(MCPH)·(MeOH)_4_]^+^, representing the molecular ion assembled with one additional MCPH molecule and four MeOH molecules. The other peak at *m/z* 1847.6045 corresponds to a cluster fragment identified as [CuNi_6_(MCP)_12_·(MCP)]^+^, which likely results from the loss of two iodide ions and one copper atom from the parent cluster along with the attachment of one MCP ligand (Figure S24, Supporting Information). It is noted that no characteristic peak was observed in the ESI‐MS spectrum in negative ion mode for either of these clusters, indicating the absence of an anionic species.

IR and Raman spectroscopic analyses were undertaken for further confirmation of the compounds’ identity and purity. Comparative IR spectra of compounds **1** and **2a** (Figure S25, Supporting Information) show C─H vibrational bands of the pyridine rings centered at 3045 cm^−1^ for both compounds. The weak appearance of this vibrational band and reduced peak splitting might be due to the unique confinement of the MCP ligands outside the cluster core. There are prominent vibrational bands for C═C (1577 and 1544 cm^−1^), C─N (1264 cm^−1^), and C═N (1668 and 1680 cm^−1^) bonds observed for **1**, along with the C═C (1575 and 1548 cm^−1^), C─N (1260 and 1240 cm^−1^), and C═N (1670 cm^−1^) bands detected for **2a** (Figure S25, Supporting Information).

Comparative Raman spectra displayed identical signatures across three different crystals for each compound (Figures S26 and S27, Supporting Information for **1** and **2a**, respectively). The spectra show C═C/C═N stretching bands at 1547 and 1581 cm^−1^ for **1** and at 1552 and 1579 cm^−1^ for **2a**, along with the strong pyridine ring breathing vibration observed ≈1015 cm^−1^ in both clusters (Figure S28, Supporting Information). Table S8 (Supporting Information) summarizes spectral assignments of the Raman bands. Notably, crystals of **1** exhibit several low frequency vibrational modes at 123, 91, and 74 cm^−1^, corresponding to the combined contribution of the cluster core (Ni─Ni, Ni─S, and Cu─S) vibrations, while the other cluster displays low frequency bands at 255, 204, 123, 98, 70, and 60 cm^−1^ related to Ni─S, Cu─S, and Cu─I vibrational modes.^[^ ^77^, ^78^ ^]^ This difference confirms the presence of different types of metal–sulfide bonding in the two clusters detected by quantum chemistry. A clear assignment of these bands is not possible due to the mixing of several vibrational modes.

**Figure 5:**
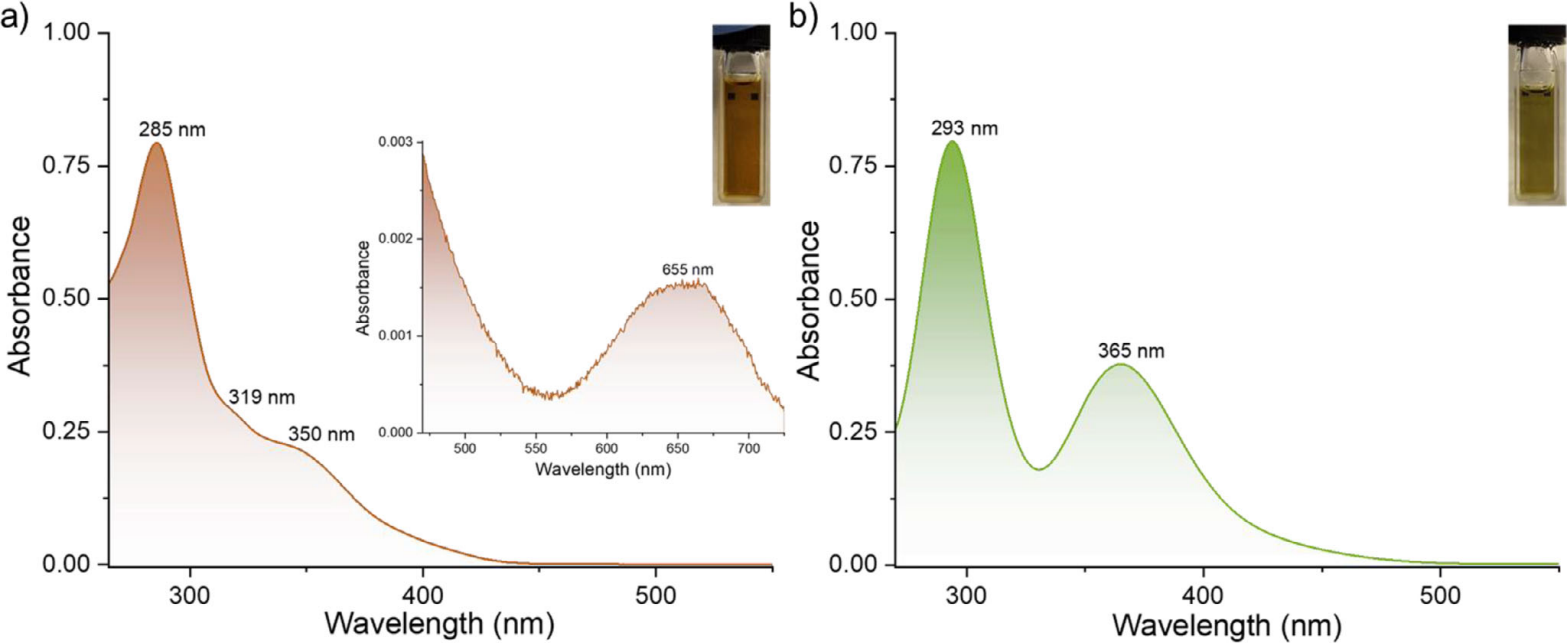
Optical absorption spectra of a) **1** and b) **2a** in DMF solution. The inset shows an enlarged view of the absorption tail of **1**, along with a photographic image of the respective clusters in DMF solutions. Absorption spectra were recorded at concentrations below saturation.

The electronic situation of these clusters was additionally inspected by means of UV–vis absorption spectra of **1** and **2a** in their respective solutions. A DMF solution of **1** exhibits one prominent absorption band centered at 285 nm (4.35 eV) and two weak bands at 319 nm (3.89 eV) and 350 nm (3.54 eV) in a characteristic pattern (**Figure** **5**a). We also observed a weak absorption band centered ≈655 nm (1.89 eV) (inset of Figure 5a). On the other hand, a solution of compound **2a** in DMF has two strong absorption bands centered ≈293 nm (4.23 eV) and 365 nm (3.39 eV) (Figure 5b).

We compared the absorption spectra of the clusters to that of the starting materials, as shown in Figure S31 (Supporting Information). The absorption spectrum of NiCl_2_ exhibits distinct peaks at 271, 408, 696, and 746 nm. In contrast, neither MCPH nor the mixture of MCPH, CuI, and NiCl_2_ shows any clear absorption bands. Comparative analysis of these absorption spectra alongside our {Cu_2_Ni_6_} clusters shows that the charge transfer bands characteristic of the specific cluster are more prominent than the strong d–d transition bands typically seen in Ni(II) compounds. The low‐energy optical absorption bands occurring below 400 nm suggest ligand centered π→π* transitions and ligand‐to‐metal charge transfer (LMCT), while the low‐intensity band ≈655 nm is likely to originate from d‐d transitions within the {Ni_6_} core of compound **1**. Theoretical studies show that optical absorption bands between 340 and 400 nm in thiolated Ni─Pt bimetallic clusters are related to the S(3p,σ)→Ni(3d) transition.^[^ ^27^ ^]^ Extrapolation of the absorption band edges yielded band gaps of 3.00 eV for compound **1** and 2.89 eV for compound **2a**, respectively (Figure S32, Supporting Information).

**Figure 6:**
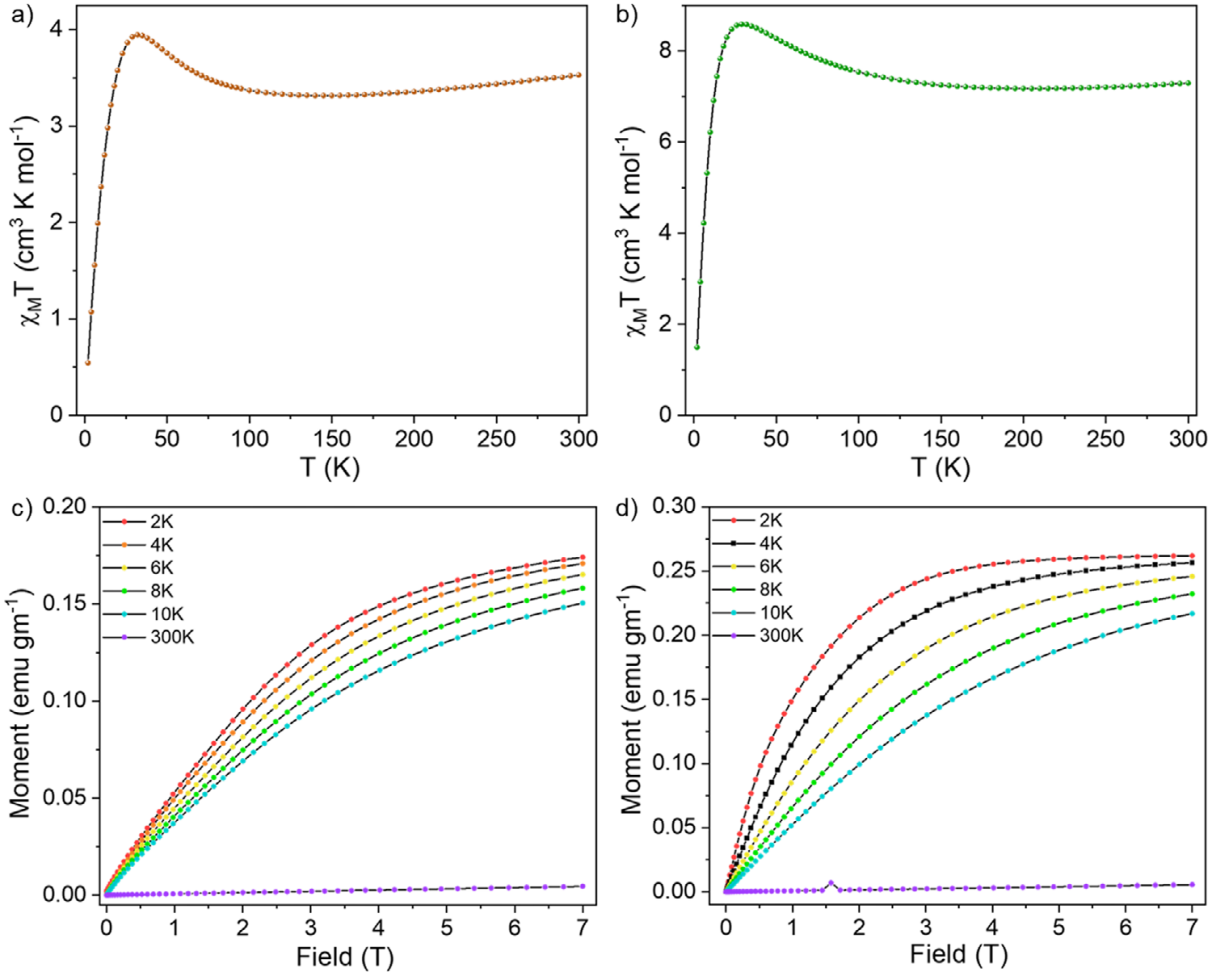
Temperature‐dependent variation of *χ_m_T* for a) **1** and b) **2a** over temperature ranging from 300 to 2 K upon an applied magnetic field of 7 T. Variable temperature magnetization (below 10 K) plots for compounds c) **1** and d) **2a** during a gradual increase in applied magnetic fields up to 7 T. Scattered points: experimental results and solid line: guide to the eye.

Moreover, the magnetic properties of the clusters were investigated using the VSM superconducting quantum interference device (SQUID) technique. Specific details of the instrument are shown in the Supporting Information. Temperature‐dependent magnetic susceptibility measurements for these clusters were carried out over the temperature range from 300 to 2 K under an applied magnetic field of 7 T. At room temperature **1** exhibits a molar magnetic susceptibility times temperature (*χ_m_T*) value of 3.52 cm^3^·K·mol^−1^ (**Figure** **6**a). According to earlier reports, this value is significantly lower than the expected value (6.60 cm^3^·K·mol^−1^) for the combination of six independent Ni(II) centers (spin state of *S* = 1 and *g* value of 2.1) beside two Cu(0) centers.^[^ ^79^, ^80^ ^]^ Upon lowering the temperature, *χ_m_T* gradually decreases up to 100 K, and it reaches to a lowest value of 3.315 cm^3^·K·mol^−1^. Lowering of *χ_m_T* upon reducing the temperature (300 to 130 K) suggests weak antiferromagnetic coupling in compound **1**. Further reducing the temperature shows an upward trend of *χ_m_T* up to 30 K, which attend a maximum value of 3.945 cm^3^·K·mol^−1^. Such enhancement of *χ_m_T* upon lowering the temperature suggests relatively strong ferromagnetic coupling interactions of various Ni(II) centers present in the non‐oligocyclic [Cu_2_Ni_6_S_3_(MCP)_6_]_n_ chain. Further lowering the temperature below 30 K showed a gradual downward trends of *χ_m_T*, which might be due to zero‐field splitting and Zeeman effects.

On the other hand, at room temperature, **2a** exhibits a *χ_m_T* value of 7.47 cm^3^·K·mol^−1^ under an applied magnetic field of 7 T applied field (Figure 6b). This value is higher than that of six uncoupled Ni(II) and two Cu(I) ions (6.61 cm^3^ K mol^−1^, *g* = 2.1). This suggests the lack of antiferromagnetic coupling in the cluster. As the temperature decreases, *χ_m_T* shows a slight downward trend, reaching 7.18 cm^3^·K·mol^−1^ at 200 K. With further cooling, *χ_m_T* increases steadily, reaching a pronounced maximum of 8.79 cm^3^·K·mol^−1^ at 30 K. The increase in *χ_m_T* with decreasing temperature suggests ferromagnetic coupling among spin active Ni(II) ions present in the hexagonal ring of the cluster. Below this temperature, the value declines and drops to 1.496 cm^3^·K·mol^−1^ at 2 K. The subsequent drop at lower temperatures is likely due to the combination of zero‐field splitting and Zeeman effects. A similar type of magnetic behavior has previously been observed in other nickel clusters.^[^ ^81^, ^82^ ^]^ Similar behaviors of *χ_m_T* were also noted under an applied field of 3 T for both compounds (Figure S33, Supporting Information).

Further upon, we calculated the effective magnetic moment (*µ_eff_*) using the following Equation (1):

(1) $$\mu_{\mathrm{eff}}=\sqrt{\frac{3k_{B}\chi T}{N_{A}\mu_{B}^{2}}}$$

(Where, *K_B_* = Boltzmann constant, *N_A_* = Avogadro constant and *µ_B_* = Bohr magneton)

At 30 K, we determined the effective magnetic moment to be 6.29 *µ_B_* for compound **1** and 9.38 *µ_B_* for **2a**, by following the Equation (1). From the magnetic moment, we calculated the ground spin state of the cluster using the Equation (2):

(2) $$\mu_{\mathrm{eff}}=g\sqrt{S\left(S+1\right)}$$

For **1**, where all six Ni(II) ions are present in a trigonal bipyramidal coordination, having a ground state of *S* = 2 suggests having the spin of four Ni(II) centers in one direction, while the remaining spins for two Ni centers point in the opposite direction. In compound **2a**, the six Ni(II) centers exhibit an octahedral coordination. This results in an overall spin state of *S* = 4, which suggests five spins in one and the remaining spins in the opposite direction. We measured the DC magnetization curves for these compounds (Figure 6c,d) as the temperature was lowered from 300 to 2 K. Both compounds demonstrate a trend for an increasing magnetic moment as the applied magnetic field is gradually increased from 1 T to 7 T. For compound **1**, no saturation was observed up to 30 K. At 10 K, we detected a saturated magnetic moment of 0.151 emu gm^−1^ at an applied field of 7 T. In contrast, compound **2a** exhibited a saturated magnetic moment of 0.2157 emu gm^−1^ at the same field strength of 7 T. An enhancement of the magnetic moment was also observed in field dependent magnetization measurements shown in Figure S34 (Supporting Information).

## 3 Conclusion

In summary, we have successfully synthesized two new compounds with copper‐nickel nanoclusters stabilized by MCP ligands, via solvothermal treatment of CuI, NiCl_2_, and MCPH. Single‐crystal X‐ray diffraction experiments and density functional theory calculations reveal the molecular structures of these clusters. The non‐oligocyclic cluster unit in **1** exhibits a trigonal prismatic {Ni_6_} core with two capping Cu atoms, while the oligocyclic cluster **2** features a crown‐like hexagonal bipyramidal {Cu_2_Ni_6_} skeleton. **2** crystallizes either in the triclinic (**2a**) or in the monoclinic (**2b**, with additional solvent molecules) crystal system, exhibiting distinctly different versions of the packing of the cluster molecules, and thus suggesting *pseudo*‐polymorphism. ESI‐MS, and various other spectroscopic and microscopic analyses, provided additional information on the molecular properties of the title compounds. These clusters exhibited distinct multiband absorption features with an optical band gap of 3.0 and 2.89 eV, respectively. Temperature‐dependent magnetic measurements showed dual antiferromagnetic and ferromagnetic coupling behavior for **1**, with a room temperature *χ_m_T* value of 3.52 cm^3^·K·mol^−1^ and a ground spin state of *S* = 2. In contrast, **2a** exhibits only ferromagnetic coupling behavior, with a room temperature *χ_m_T* value of 7.47 cm^3^·K·mol^−1^ and a ground spin state of *S* = 4. This study is anticipated to provide valuable insights into the synthesis of mixed‐metallic nickel‐based nanoclusters while also contributing to the development of active and durable magnetic nanomaterials for advanced technological applications.

## 4 Experimental Section

### Chemicals Used

Anhydrous nickel(II) chloride (98%) and copper(I) iodide were purchased from Thermo Scientific Chemicals and Merck Chemicals, respectively. 2‐mercaptopyridine (98 %) was bought from Apollo Scientific. Extra‐dry *N,N*‐dimethylformamide (99.8 %) and methanol were from Thermo Scientific and Merck KGaA, respectively. All the chemicals were commercially available and used as such without further purification. Yields were calculated based on collected single crystals of the respective compounds. For further studies, the respective crystals were manually separated using a microneedle.

### Solvothermal Synthesis of 1 and 2

The compounds were produced through a one‐step solvothermal synthesis involving anhydrous NiCl_2_, CuI, and MCPH. A borosilicate glass ampule was loaded with 15.5 mg (120 mmol) of anhydrous NiCl_2_, 13 mg (70 mmol) of CuI and 30 mg (270 mmol) of MCPH. The filled ampule was evacuated for 30 min at a pressure of 0.05 mbar. Under a continuous flow of argon, 800 µL of DMF and 200 µL of MeOH were added. After 20 min of ultrasonication, the yellow‐colored solution was flash‐frozen in a liquid nitrogen‐filled dewar, evacuated (≈20 min) to an internal pressure of 0.05 mbar, and then flame‐sealed using a propane burner. The sealed ampule was placed in a sand bath, which was then heated to 120 °C over a period of 72 h. The heating ramp was set at 30 °C per hour, and the cooling ramp was set at 5 °C per hour down to room temperature. After the reaction, a mixture of crystals was observed at the bottom of the ampule. The crystals were thoroughly washed with MeOH and then dried by self‐evaporation of the solvent under ambient conditions inside the fume hood. The crystallized compounds **1** and **2** were obtained with yields of ≈38% and ≈45% (40% **2a**, and 5% **2b**), respectively. No degradation of the crystals was observed during this process and also at room temperature. Both compounds were stable under ambient atmosphere at laboratory conditions. Raman studies and µ‐XRF analysis demonstrate that both compounds remain stable under ambient conditions for over six months (Figures S29 and S30, Supporting Information).

Attempts to synthesize the compounds selectively were undertaken by a series of reactions under variation of the concentration and stoichiometry of NiCl_2_:CuI (x:1, with x = 0.86, 1.71, and 3.43), as well as the reaction temperature (80, 100, 120 °C), while the solvent systems, the amount of MCPH, and the reaction time (72 h) remained constant. Based on the observations, which were summarized in Table S9 (Supporting Information), it was concluded that compounds **2a** and **2b** predominantly form during reactions at 80 °C, while no crystals of compound **1** and **A** were observed under these conditions. Increasing the amount of CuI enhances the synthetic yield of compound **A**. Raising the temperature to 100 °C results in the formation of compound **1**, along with crystals of compounds **2a**, **2b**, and **A**. At 120 °C, a significant quantity of both compounds **1** and **2a** was observed to be present in the ampule.

## Conflict of Interest

The authors declare no conflict of interest.

## Supporting information



Supporting Information

Supplemental cif

## Data Availability

The data that support the findings of this study are available from the corresponding author upon reasonable request.

## References

[1] R. Jin , C. Zeng , M. Zhou , Y. Chen , Chem. Rev. 2016, 116, 10346. 27585252 10.1021/acs.chemrev.5b00703

[2] I. Chakraborty , T. Pradeep , Chem. Rev. 2017, 117, 8208. 28586213 10.1021/acs.chemrev.6b00769

[3] O. Fuhr , S. Dehnen , D. Fenske , Chem. Soc. Rev. 2013, 42, 1871. 22918377 10.1039/c2cs35252d

[4] P. Jena , Q. Sun , Chem. Rev. 2018, 118, 5755. 29812916 10.1021/acs.chemrev.7b00524

[5] S. Maity , S. Kolay , S. Chakraborty , A. Devi , N. Rashi , A. Patra , Chem. Soc. Rev. 2025, 54, 1785. 39670813 10.1039/d4cs00962b

[6] X. Kang , M. Zhu , Chem. Soc. Rev. 2019, 48, 2422. 30838373 10.1039/c8cs00800k

[7] N. Rinn , I. Rojas‐León , B. Peerless , S. Gowrisankar , F. Ziese , N. W. Rosemann , W. C. Pilgrim , S. Sanna , P. R. Schreiner , S. Dehnen , Chem. Sci. 2024, 15, 9438. 38939157 10.1039/d4sc01136h PMC11206280

[8] K. Kwak , D. Lee , Acc. Chem. Res. 2019, 52, 12. 30500153 10.1021/acs.accounts.8b00379

[9] L. Chen , A. Black , W. J. Parak , I. Chakraborty , Aggregate 2022, 3, e132.

[10] W. Fei , S. Y. Tang , M. B. Li , Nanoscale 2024, 16, 19589. 39359125 10.1039/d4nr03111c

[11] F. Tian , J. Chen , F. Chen , Y. Liu , Y. Xu , R. Chen , Appl. Catal. B Environ. 2021, 292, 120158.

[12] Q. J. Wu , D. H. Si , P. P. Sun , Y. L. Dong , S. Zheng , Q. Chen , S. H. Ye , D. Sun , R. Cao , Y. B. Huang , Angew. Chem. – Int. Ed. 2023, 62, 202306822. 10.1002/anie.202306822 37468435

[13] M. P. Maman , T. Gurusamy , A. K. Pal , R. Jana , K. Ramanujam , A. Datta , S. Mandal , Angew. Chem. – Int. Ed. 2023, 62, 202305462. 10.1002/anie.202305462 37129995

[14] S. Srinivasan , Z. Liu , S. House , R. Jin , Inorg. Chem. 2023, 62, 1875. 35862896 10.1021/acs.inorgchem.2c01292

[15] J. Hu , Y. M. Li , B. Zhang , X. Kang , M. Zhu , Inorg. Chem. Front. 2024, 11, 4974.

[16] A. Jose , A. Jana , T. Gupte , A. S. Nair , K. Unni , A. Nagar , A. R. Kini , B. K. Spoorthi , S. K. Jana , B. Pathak , T. Pradeep , ACS Mater. Lett. 2023, 5, 893.

[17] P. D. Jadzinsky , G. Calero , C. J. Ackerson , D. A. Bushnell , R. D. Kornberg , Science 2007, 318, 430. 17947577 10.1126/science.1148624

[18] X. Kang , H. Chong , M. Zhu , Nanoscale 2018, 10, 10758. 29873658 10.1039/c8nr02973c

[19] A. Desireddy , B. E. Conn , J. Guo , B. Yoon , R. N. Barnett , B. M. Monahan , K. Kirschbaum , W. P. Griffith , R. L. Whetten , U. Landman , T. P. Bigioni , Nature 2013, 501, 399. 24005327 10.1038/nature12523

[20] Q. Zhou , S. Kaappa , S. Malola , H. Lu , D. Guan , Y. Li , H. Wang , Z. Xie , Z. Ma , H. Häkkinen , N. Zheng , X. Yang , L. Zheng , Nat. Commun. 2018, 9, 2948. 30054489 10.1038/s41467-018-05372-5 PMC6063937

[21] T. Jia , Z. J. Guan , C. Zhang , X. Z. Zhu , Y. X. Chen , Q. Zhang , Y. Yang , D. Sun , J. Am. Chem. Soc. 2023, 145, 10355. 37104621 10.1021/jacs.3c02215

[22] R. W. Huang , J. Yin , C. Dong , A. Ghosh , M. J. Alhilaly , X. Dong , M. N. Hedhili , E. Abou‐Hamad , B. Alamer , S. Nematulloev , Y. Han , O. F. Mohammed , O. M. Bakr , J. Am. Chem. Soc. 2020, 142, 8696. 32315164 10.1021/jacs.0c00541

[23] T. W. Hayton , R. Jin , D. Jiang , in Atomically Precise Nanochemistry , R. Jin , D. Jiang (Eds) Wiley, 2023, 285.

[24] A. W. Cook , T. W. Hayton , Acc. Chem. Res. 2018, 51, 2456. 30240192 10.1021/acs.accounts.8b00329

[25] Y. Pan , J. Chen , S. Gong , Z. Wang , Dalt. Trans. 2018, 47, 11097. 10.1039/c8dt02059k 30040107

[26] A. Muñoz‐Castro , Chem. Sci. 2014, 5, 4749.

[27] T. Okada , T. Kawawaki , K. Takemae , S. Tomihari , T. Kosaka , Y. Niihori , Y. Negishi , J. Phys. Chem. Lett. 2024, 15, 1539. 38299566 10.1021/acs.jpclett.3c03594 PMC10860137

[28] N. A. Torquato , J. M. Palasz , Q. C. Bertrand , F. M. Brunner , T. Chan , M. Gembicky , A. A. Mrse , C. P. Kubiak , Chem. Sci. 2022, 13, 11382. 36320577 10.1039/d2sc04042e PMC9533397

[29] B. K. Breedlove , P. E. Fanwick , C. P. Kubiak , Inorg. Chem. 2002, 41, 4306. 12184742 10.1021/ic020108a

[30] A. Ceriotti , R. Della Pergola , S. L. Garlaschelli , M. Manassero , N. Masciocchi , M. Sansonib , J. Chem. Soc. Dalt. Trans. 1991, 6, 2357.

[31] C. Femoni , M. C. Iapalucci , G. Longoni , P. H. Svensson , Chem. Commun. 2000, 4, 655. 10.1039/b103610f 12240310

[32] B. Nowicka , K. Stadnicka , W. Nitek , M. Rams , B. Sieklucka , CrystEngComm 2012, 14, 6559.

[33] J. G. Brennan , T. Siegrist , Y. U. Kwon , S. M. Stuczynski , M. L. Steigerwald , J. Am. Chem. Soc. 1992, 114, 10334.

[34] M. M. Shoshani , S. A. Johnson , Nat. Chem. 2017, 9, 1282.

[35] D. Fenske , J. Hachgenei , J. Ohmer , Angew. Chemie – Int. Ed. 1985, 24, 706.

[36] P. R. Hertler , A. J. Touchton , G. Wu , T. Chang , Y. P. Chen , Y. S. Chen , T. W. Hayton , Inorg. Chem. 2025, 64, 2926. 39900359 10.1021/acs.inorgchem.4c05088

[37] X. Hang , B. Liu , X. Zhu , S. Wang , H. Han , W. Liao , Y. Liu , C. Hu , J. Am. Chem. Soc. 2016, 138, 2969. 26894471 10.1021/jacs.6b00695

[38] B. Biswas , U. Pieper , T. Weyhermüller , P. Chaudhuri , Inorg. Chem. 2009, 48, 6781. 19552389 10.1021/ic9007608

[39] J. Esteban , L. Alcázar , M. Torres‐Molina , M. Monfort , M. Font‐Bardia , A. Escuer , Inorg. Chem. 2012, 51, 5503. 22564061 10.1021/ic3004036

[40] A. Gehin , S. Ferlay , J. M. Harrowfield , D. Fenske , N. Kyritsakas , M. W. Hosseini , Inorg. Chem. 2012, 51, 5481. 22515471 10.1021/ic300550v

[41] M. Chen , M. Zhang , X. Wang , Y. Bi , B. Chen , Z. Zheng , Inorg. Chem. 2019, 58, 6276. 30990033 10.1021/acs.inorgchem.9b00495

[42] S. Wang , X. Gao , X. Hang , X. Zhu , H. Han , W. Liao , W. Chen , J. Am. Chem. Soc. 2016, 138, 16236. 27935678 10.1021/jacs.6b11218

[43] S. Muche , I. Levacheva , O. Samsonova , L. Pham , G. Christou , U. Bakowsky , Inorg. Chem. 2014, 53, 7642. 24992258 10.1021/ic500957y

[44] A. Bhanja , R. Herchel , Z. Trávníček , D. Ray , Inorg. Chem. 2019, 58, 12184. 31483643 10.1021/acs.inorgchem.9b01517

[45] M. Kawano , J. W. Bacon , C. F. Campana , B. E. Winger , J. D. Dudek , S. A. Sirchio , S. L. Scruggs , U. Geiser , L. F. Dahl , Inorg. Chem. 2001, 40, 2554. 11350234 10.1021/ic000979p

[46] M. Kawano , J. W. Bacon , C. F. Campana , L. F. Dahl , J. Am. Chem. Soc. 1996, 118, 7869.

[47] H. M. Zhang , Y. G. Li , Y. Lu , R. Clérac , Z. M. Zhang , Q. Wu , X. J. Feng , E. B. Wang , Inorg. Chem. 2009, 48, 10889. 19943688 10.1021/ic901552f

[48] X. B. Han , Y. G. Li , Z. M. Zhang , H. Q. Tan , Y. Lu , E. B. Wang , J. Am. Chem. Soc. 2015, 137, 5486. 25866996 10.1021/jacs.5b01329

[49] T. Hamaguchi , M. D. Doud , J. Hilgar , J. D. Rinehart , C. P. Kubiak , Dalt. Trans. 2016, 45, 2374. 10.1039/c5dt04861c 26750262

[50] J. Ji , G. Wang , T. Wang , X. You , X. Xu , Nanoscale 2014, 6, 9185. 24981393 10.1039/c4nr01063a

[51] A. M. S. Pembere , C. Cui , R. Anumula , H. Wu , P. An , T. Liang , Z. Luo , Phys. Chem. Chem. Phys. 2019, 21, 17933. 31380877 10.1039/c9cp02964h

[52] H. N. Kagalwala , E. Gottlieb , G. Li , T. Li , R. Jin , S. Bernhard , Inorg. Chem. 2013, 52, 9094. 23865570 10.1021/ic4013069

[53] C. Tan , M. Jin , X. Ma , Q. Zhu , Y. Huang , Y. Wang , S. Hu , T. Sheng , X. Wu , Dalt. Trans. 2012, 41, 8472. 10.1039/c2dt30524k 22653469

[54] C. Tan , M. Jin , H. Zhang , S. Hu , T. Sheng , X. Wu , CrystEngComm 2015, 17, 5110.

[55] M. M. Shoshani , R. Beck , X. Wang , M. J. McLaughlin , S. A. Johnson , Inorg. Chem. 2018, 57, 2438. 29140692 10.1021/acs.inorgchem.7b02546

[56] Z. Wang , Z. Jagličić , L. L. Han , G. L. Zhuang , G. G. Luo , S. Y. Zeng , C. H. Tung , D. Sun , CrystEngComm 2016, 18, 3462.

[57] A. J. Touchton , G. Wu , T. W. Hayton , Chem. Sci. 2022, 13, 5171. 35655571 10.1039/d2sc00960a PMC9093199

[58] A. J. Touchton , G. Wu , T. W. Hayton , Inorg. Chem. 2021, 60, 17586. 34762406 10.1021/acs.inorgchem.1c02184

[59] M. G. Hilfiger , H. Zhao , A. Prosvirin , W. Wernsdorfer , K. R. Dunbar , Dalt. Trans. 2009, 5155. 10.1039/b903691a 19562176

[60] T. Ochsenbein , M. Murrie , E. Rusanov , H. Stoeckli‐Evans , C. Sekine , H. U. Güdel , Inorg. Chem. 2002, 41, 5133. 12354047 10.1021/ic020252w

[61] M. K. Wojnar , D. W. Laorenza , R. D. Schaller , D. E. Freedman , J. Am. Chem. Soc. 2020, 142, 14826. 32786760 10.1021/jacs.0c06909

[62] C. H. Lee , L. Liu , C. Bejger , A. Turkiewicz , T. Goko , C. J. Arguello , B. A. Frandsen , S. C. Cheung , T. Medina , T. J. S. Munsie , R. D'Ortenzio , G. M. Luke , T. Besara , R. A. Lalancette , T. Siegrist , P. W. Stephens , A. C. Crowther , L. E. Brus , Y. Matsuo , E. Nakamura , Y. J. Uemura , P. Kim , C. Nuckolls , M. L. Steigerwald , X. Roy , J. Am. Chem. Soc. 2014, 136, 16926. 25379957 10.1021/ja5098622

[63] C. Papatriantafyllopoulou , E. E. Moushi , G. Christou , A. J. Tasiopoulos , Chem. Soc. Rev. 2016, 45, 1597. 26767319 10.1039/c5cs00590f

[64] Y. Li , R. Jin , J. Phys. Chem. C 2021, 125, 15773.

[65] M. Zhu , C. M. Aikens , M. P. Hendrich , R. Gupta , H. Qian , G. C. Schatz , R. Jin , J. Am. Chem. Soc. 2009, 131, 2490. 19178295 10.1021/ja809157f

[66] V. G. Albano , F. Demartin , C. Femoni , M. C. Iapalucci , G. Longoni , M. Monari , P. Zanello , J. Organomet. Chem. 2000, 593–594, 325.

[67] A. K. Boudalis , M. Pissas , C. P. Raptopoulou , V. Psycharis , B. Abarca , R. Ballesteros , Inorg. Chem. 2008, 47, 10674. 18925738 10.1021/ic801441d

[68] B. Peng , X. Zhang , D. G. A. L. Aarts , R. P. A. Dullens , Nat. Nanotechnol. 2018, 13, 478. 29610527 10.1038/s41565-018-0108-0

[69] K. Chakarawet , M. Atanasov , J. Marbey , P. C. Bunting , F. Neese , S. Hill , J. R. Long , J. Am. Chem. Soc. 2020, 142, 19161. 33111523 10.1021/jacs.0c08460

[70] M. Chibani , S. Benamara , H. Zitoune , M. Lasmi , L. Benchalal , L. Lamiri , M. Samah , Int. J. Quantum Chem. 2025, 125, e70007.

[71] G. Pacchioni , N. Rosch , Acc. Chem. Res. 1995, 28, 390.

[72] S. Kamal , A. I. Inamdar , K. R. Chiou , A. Pathak , A. W. Yibeltal , J. W. Chen , W. F. Liaw , M. Hayashi , B. Sainbileg , C. H. Hung , K. L. Lu , J. Phys. Chem. C 2022, 126, 6300.

[73] J.‐P. Dong , Y. Xu , X.‐G. Zhang , H. Zhang , L. Yao , R. Wang , S.‐Q. Zang , Angew. Chemie – Int. Ed. 2023, 62, e202313648. 10.1002/anie.202313648 37801352

[74] Y. Li , M. Zhou , Y. Song , T. Higaki , H. Wang , R. Jin , Nature 2021, 594, 380. 34135522 10.1038/s41586-021-03564-6

[75] H. Li , P. Wang , C. Zhu , W. Zhang , M. Zhou , S. Zhang , C. Zhang , Y. Yun , X. Kang , Y. Pei , M. Zhu , J. Am. Chem. Soc. 2022, 144, 23205. 36484475 10.1021/jacs.2c11341

[76] G. Dong , Z. Pan , B. Han , Y. Tao , X. Chen , G.‐G. Luo , P. Sun , C. Sun , D. Sun , Angew. Chemie – Int. Ed. 2023, 62, e202302595. 10.1002/anie.202302595 37052323

[77] K. R. Krishnadas , A. Baghdasaryan , R. Kazan , E. Banach , J. Teyssier , V. P. Nicu , T. Buergi , Small 2021, 17, 2101855. 10.1002/smll.202101855 34405952

[78] X. Wang , Y. Zhong , T. Li , K. Wang , W. Dong , M. Lu , Y. Zhang , Z. Wu , A. Tang , X. Bai , Nat. Commun. 2025, 16, 587. 39799142 10.1038/s41467-025-55975-y PMC11724975

[79] J. Y. Xu , X. Qiao , H. Bin Song , S. P. Yan , D. Z. Liao , S. Gao , Y. Journaux , J. Cano , Chem. Commun. 2008, 8, 6414. 10.1039/b813705f 19048173

[80] A. K. Ghosh , M. Pait , M. Shatruk , V. Bertolasi , D. Ray , Dalt. Trans. 2014, 43, 1970. 10.1039/c3dt52999a 24296683

[81] C. G. Efthymiou , L. Cunha‐Silva , S. P. Perlepes , E. K. Brechin , R. Inglis , M. Evangelisti , C. Papatriantafyllopoulou , Dalt. Trans. 2016, 45, 17409. 10.1039/c6dt03511f 27731458

[82] J. L. Wang , Y. Bai , H. Pan , G. S. Zheng , D. B. Dang , Dalt. Trans. 2017, 46, 12771. 10.1039/c7dt02889j 28926046

