# Supplementary material for: Bonding Features and Magnetic Ordering in Thiolate‐Bridged Copper‐Nickel Clusters Synthesized at Elevated Temperature
Source: Small. 2025 Aug 8;21(38):e06920. doi: 10.1002/smll.202506920 (PMC12462566; doi:10.1002/smll.202506920)
Supplement: Supplementary file 1 — Supporting Information [file SMLL-21-e06920-s001.pdf]

NANO · MICRO
small

Supporting Information

for *Small*, DOI 10.1002/smll.202506920

Bonding Features and Magnetic Ordering in Thiolate-Bridged Copper-Nickel Clusters Synthesized at Elevated Temperature

*Arijit Jana, Yaofeng Wang, Lukas Guggolz, Yaorong Chen, Franziska Ganslmaier, Bastian Weinert, Mario Ruben and Stefanie Dehnen**

Supporting Information

Bonding Features and Magnetic Ordering in Thiolate-Bridged Copper-Nickel Clusters Synthesized at Elevated Temperature

Arijit Jana,[1] Yaofeng Wang,[1] Lukas Guggolz,[1] Yaorong Chen,[1] Franziska Ganslmaier,[1] Bastian Weinert,[1] Mario Ruben,[1,2,3] Stefanie Dehnen[1]*

[1] Institute of Nanotechnology, Karlsruhe Institute of Technology, Kaiserstraße 12, 76131 Karlsruhe, Germany.

[2] Institute of Quantum Materials and Technologies (IQMT), Karlsruhe Institute of Technology, Kaiserstraße 12, 76131 Karlsruhe, Germany.

[3] Centre Européen de Sciences Quantiques, Institut de Science et d'Ingénierie Supramoléculaires (ISIS, UMR 7006), CNRS-Université de Strasbourg, 8 allée Gaspard Monge BP 70028 67083 Strasbourg Cedex France, France.

*Email: stefanie.dehnen@kit.edu

Table of content

1. Instrumentation

2. Methods of the quantum chemical calculations

3. Details of crystallographic analyses and micro-XRF spectroscopy

4. Quantum chemical analyses

5. High-resolution mass spectrometry, vibrational, and optical spectroscopy

6. Analyses of the magnetic properties

7. References

1. Instrumentation

Optical microscopy

Optical microscopic image of the crystals was collected using OLYMPUS SZX10 light microscope equipped with KL 1600 LED white light source and EP50 camara.

UV-visible absorption spectroscopy

Optical absorption spectra were obtained in transmission mode from the corresponding solution using a Varian Cary 5000 UV-Vis-NIR spectrometer from Agilent. The optical path length for the measurement is set to 1 cm. The spectral data were collected at a scan rate of 500 nm/min with a 1 nm data interval, using the Cary WinUV Scan Application software (version 6.2.0.1588).

Micro-X-Ray Fluorescence Spectroscopy (μ-XRF)

Micro-XRF analysis was carried out using a HORIBA XGT X-ray analytical microscope. Single crystals of the respective clusters were mounted on a plastic sheet using NVH immersion oil. Measurements were conducted under vacuum conditions (1 mbar) utilizing an Rh X-ray source operated at 50 kV and 632 μA. Fluorescence emission was recorded over a measurement time of 180 seconds. Quantitative analysis of Cu and Ni was based on their characteristic Kα X-ray emission lines. μ-XRF spectra from three independent single crystals of each sample were recorded.

Mass spectrometry

The ESI-MS has been performed on a Orbitrap Exploris 240 mass spectrometer by Thermo Fischer Scientific in the positive ion mode with application mode small molecules: Spray voltage 2.507– 4.972 kV, spray current 0.52– 4.15 μA, capillary temperature 320.01 °C, sheath gas pressure 21.27 psi, aux gas pressure 13.67 psi, sweep gas pressure 2.59 psi, C-trap RF frequency 3.203 MHz, C-trap RF amp. 2759.948 MHz and Orbitrap resolution 240,000 mass resolving power. Spectral measurements were conducted with a cluster sample concentration of approximately 1 µg/ml.

Infrared spectroscopy

Infrared spectrum was recorded using the Thermo Scientific NICOLET iS50 FTIR instrument. The crystalline samples were placed on the diamond ATR single crystal for the spectral measurements. Data collection was performed using OMNIC software.

Raman spectroscopy

Raman spectra were obtained using a Renishaw inVia confocal Raman spectrometer, equipped with a 532 nm and 785 nm laser source, diffraction gratings (532 and 785 nm), and an ultra-high sensitivity CCD detector. Objective lenses with 20x and 100x magnification were used to focus the incident beam. Data acquisition was performed using selected single crystals at room temperature using WiRE software, with the detector providing a spectral resolution of 0.5 cm^{-1}.

Single crystal X-ray diffraction

Single crystal X-ray diffraction data was collected at 150 K on a STOE STADIVARI diffractometer with Ga/K_α radiation (λ = 1.34143 Å). Data collection, integration, scaling (ABSPACK) and absorption correction were performed in X-area 2.1 software. The structures were solved using SHELXT from SHELXL-2018/136, and refined by full matrix least-squares methods against F2 with the SHELXL program. Olex2 was used for viewing and to prepare CIF files. Refinement was performed with anisotropic temperature factors for all non-hydrogen atoms. Hydrogen atoms were calculated on idealized positions. Figures were created using Diamond V4 and Mercury 2022.3.0. Solvent masks were used for back-Fourier transforming residual electron density form heavily disordered solvent molecules that could not be assigned. Details are given along with the crystallographic data.

Powder X-ray diffraction

The PXRD were measured in transmission mode using Stoe StadiP diffractometer equipped with Cu K_α X-ray radiation (λ = 1.54186 Å) source and a Mythen photodetector. Single crystals are placed inside a scotch tape for the measurement. Data measurement and analysis was performed using WinXPOW software.

Magnetic measurements

The DC magnetic measurements for compounds **1** and **2a** were performed using MPMS3 SQUID VSM magnetometer from Quantum Design. The magnetic susceptibility curves were measured as a function of temperature at two different applied magnetic fields, 3 and 7 T, after cooling down the samples from 300 K to 2 K. The results were corrected for the contributions of the sample holder and eicosane. The magnetization curves of the respective samples as a function of applied magnetic field (from 0.5 T to 7 T) were measured from 300 K to 10 K. Selected crystalline samples were finely ground using a mortar and pestle. Measured quantities of 9.2 mg for sample **1** and 13.0 mg for sample **2a** were combined with small amounts of eicosane to reduce the alignment of microcrystallites in a magnetic field. These mixtures were then loaded into gelatin capsules, which were inserted into a straw for magnetic measurements.

2. Methods of the quantum chemical calculations

Computational studies were done using the program system Turbomole V7.8[1] and employing DFT methods. We applied the hybrid functional PBE0[2] with a grid of size 3 and basis sets of quality dhf-TZVP.[3] The SCF convergence criterion was set to 10^{-8} Hartree. The resolution-of-the-identity (RI) approximation[4] and corresponding auxiliary bases[5] were used to speed the geometry optimizations up. The geometry optimizations were performed without any symmetry restrictions. The cartesian coordinates of the optimized molecular structures can be found in the file Calculated_Coordinates.txt. Mulliken[6] and natural population analyses (NPA)[7] were used to calculate partial charges. LMOs were obtained via Boys' method.[9] The optimized molecular structures were confirmed as minima on the potential energy hypersurface by calculation of force constants with the module Aoforce,[10] as implemented in Turbomole. For these follow-up studies we used a finer grid of size 5.

3. Details of the crystallographic analyses and micro-XRF spectroscopy

Table S1. Crystallographic parameters and structural refinement data for **1**.

Identification code	$[Cu_2Ni_6S_3(MCP)_6]$
Empirical formula	$C_{30}H_{24}N_6Cu_2Ni_6S_9$ [$\cdot$0.6 C_3H_7NO]
Formula weight	1280.29
Temperature/K	150
Crystal system	Orthorhombic
Space group	*Pbcn*
a/Å	19.2770(18)
b/Å	19.490(2)
c/Å	21.376(3)
α/°	90
β/°	90
γ/°	90
V/Å^3	8031.2(15)
Z	8
ρ_{calc}/cm^3	2.118
μ/mm^{-1}	23.775
Absorption correction type / T_{min} / T_{max}	multi-scan / 0.110 / 0.305
F(000)	1520
Crystal size/mm^3	0.20 × 0.10 × 0.05
Radiation	GaK$_\alpha$ (λ = 1.34143)
2θ range for data collection/°	5.61 to 125.908
Index ranges	$-25 \leq h \leq 19$, $-17 \leq k \leq 24$, $-26 \leq l \leq 28$
Reflections collected	39897
Independent reflections	9386 [R_{int} = 0.0642, R_{sigma} = 0.0750]
Data/restraints/parameters	9386/0/478
Goodness-of-fit on F^2	0.907
Final R indexes [$I >= 2\sigma (I)$]	R_1 = 0.0398, wR_2 = 0.0732
Final R indexes [all data]	R_1 = 0.0811, wR_2 = 0.0819
Largest diff. peak/hole /e Å^{-3}	0.609/−0.648
CCDC	2457678

A solvent mask was calculated and 200 electrons were found in a volume of 1016 A^3 in 2 voids per unit cell. This is consistent with the presence of 0.6 C_3H_7NO molecules per asymmetric unit (= one formula unit) and 4.8 C_3H_7NO molecules per unit cell, which account for 192 electrons per unit cell.

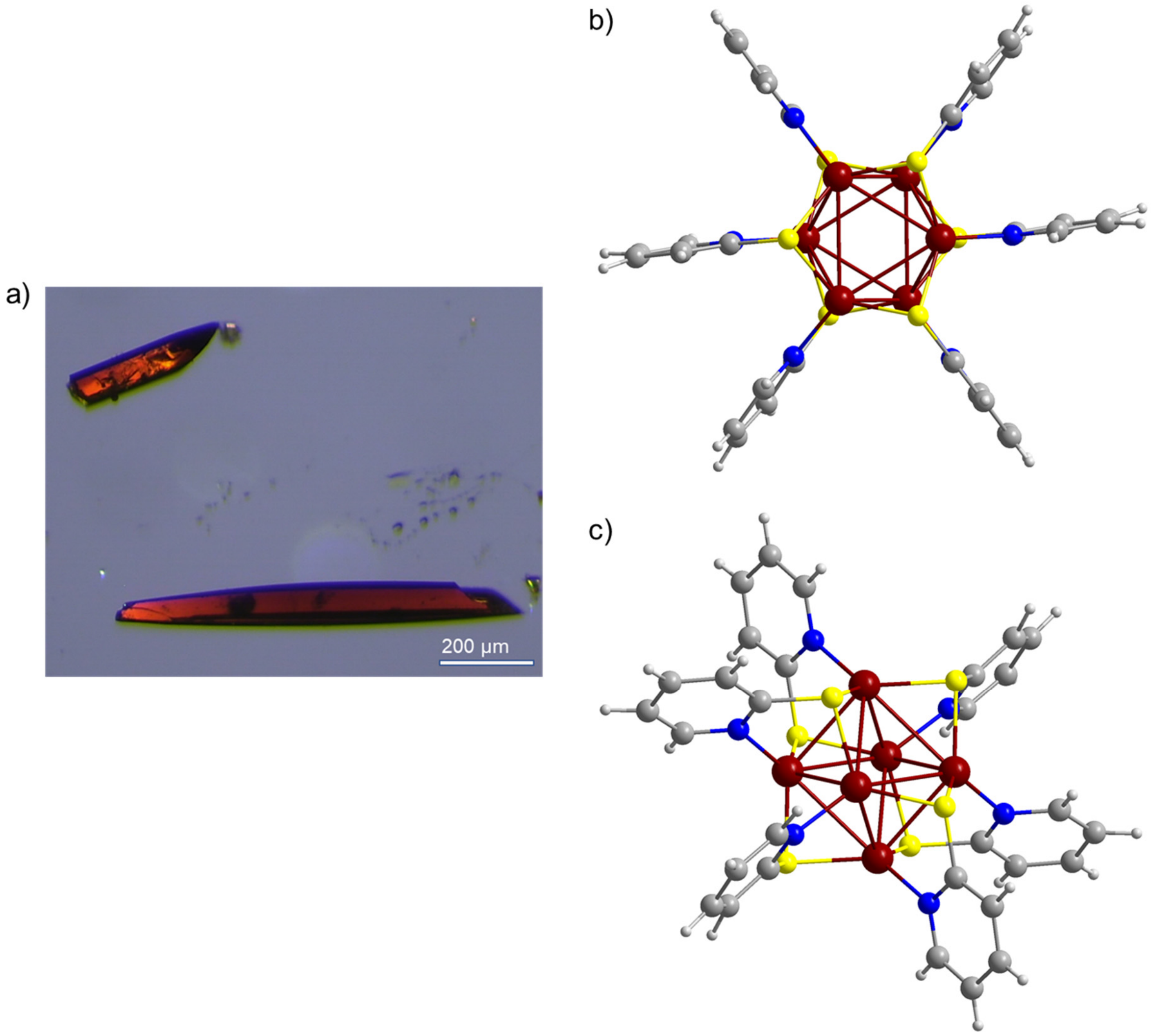

Figure S1. a) Optical photograph of single crystals of $[Cu_6(MCP)_6]$ cluster in **A**. b) and c) Complete molecular structure of the $[Cu_6(MCP)_6]$ cluster, viewed from two different orientation (dark red = Cu, yellow = S, blue = N, grey = C, white = H).

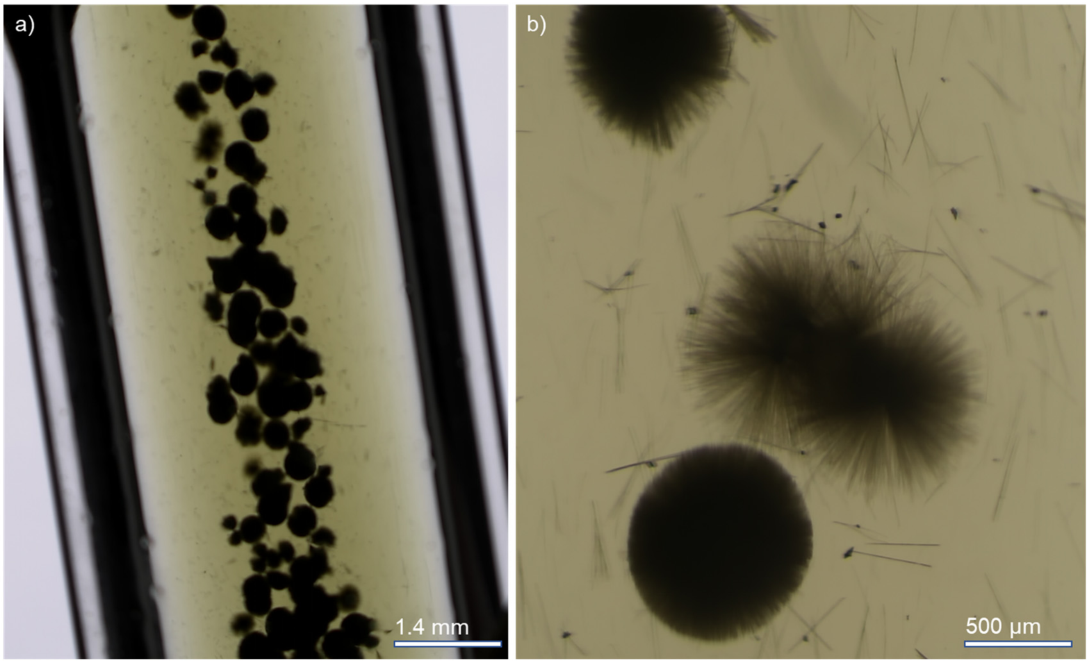

Figure S2. a) Optical photograph of the ampule reveals spherical aggregates formed by the solvothermal reaction (120 °C for 72 h) between $NiCl_2$ and MCP. b) Magnified view of the ampule displaying microneedles assembled spherical aggregates.

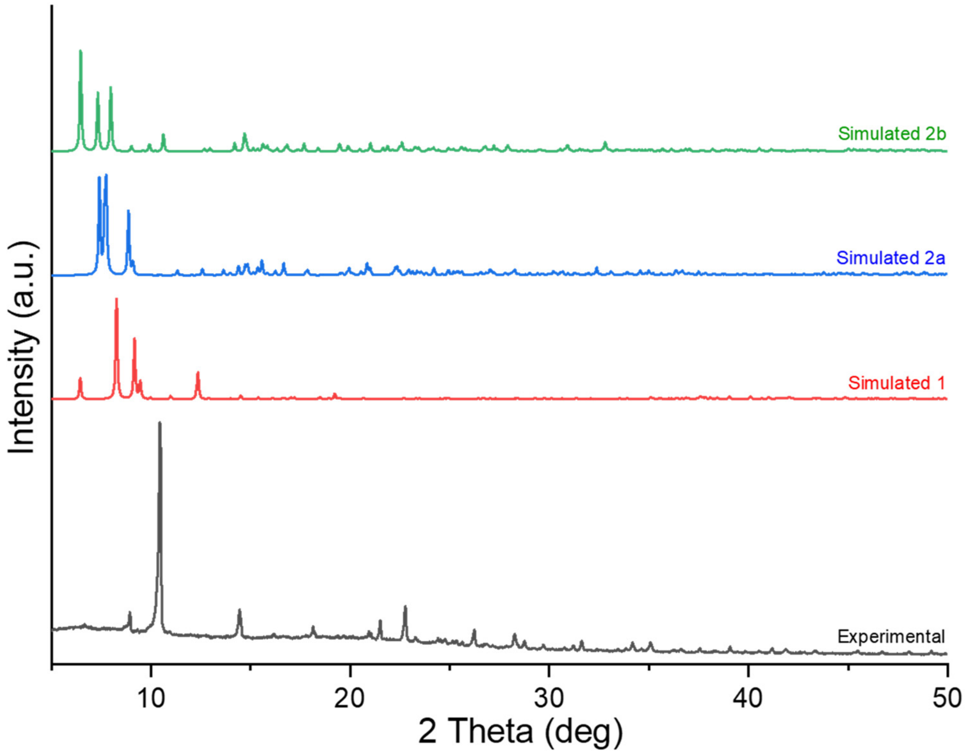

Figure S3. Experimental P-XRD pattern of the spherical aggregates from Figure S2 compared with the simulated pattern obtained from compounds **1** and **2a**, **2b**.

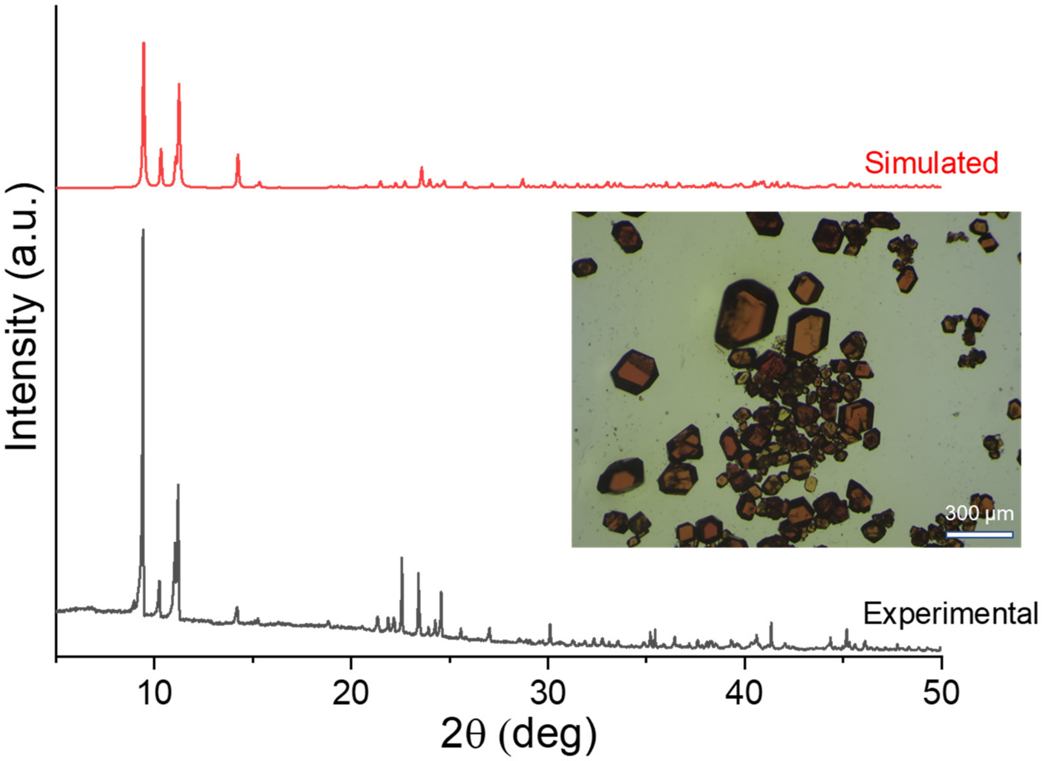

Figure S4. Comparison between the experimental P-XRD pattern of the product formed from the hydrothermal reaction of MCPH and CuI, and the simulated pattern derived from SC-XRD data of the [Cu_6MCP_6] cluster in **A**. The inset displays a photograph of the obtained crystals.

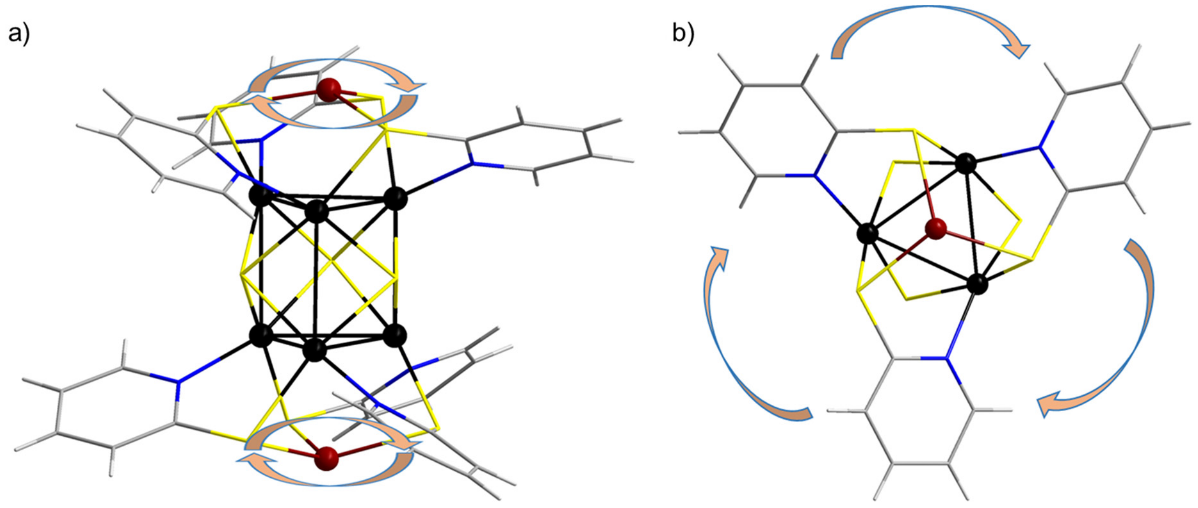

Figure S5. a) Side view of the [$Cu_2Ni_6S_3(MCP)_6$] cluster in **1**, illustrating the helical arrangement of three MCP ligands on each side. b) Top view of the cluster (black = Ni, dark red = Cu, yellow = S, blue = N, grey = C, white = H).

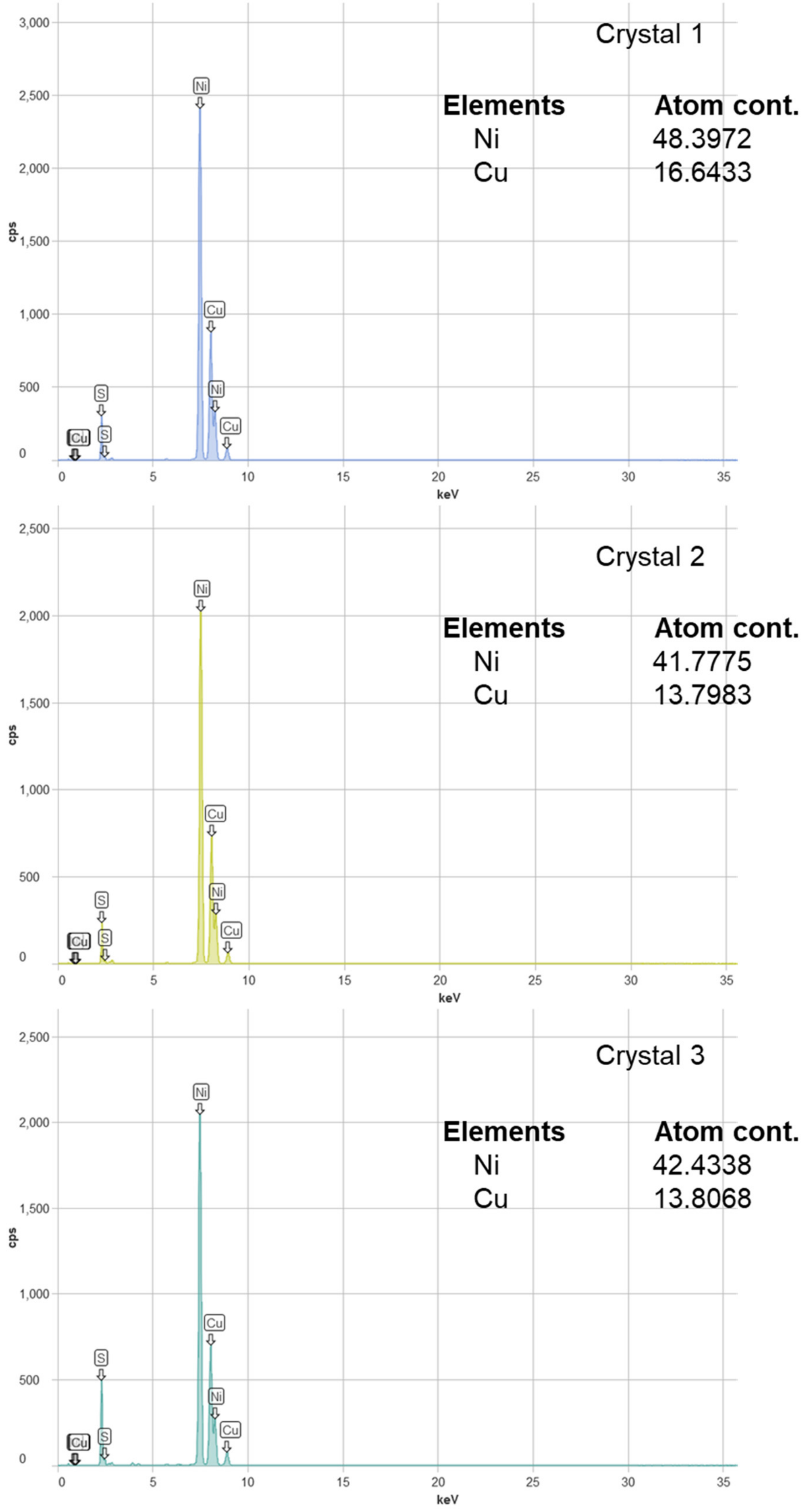

Figure S6. μ-XRF spectra of three single crystals of compound **1**, with insets displaying the Ni and Cu content in each respective crystal.

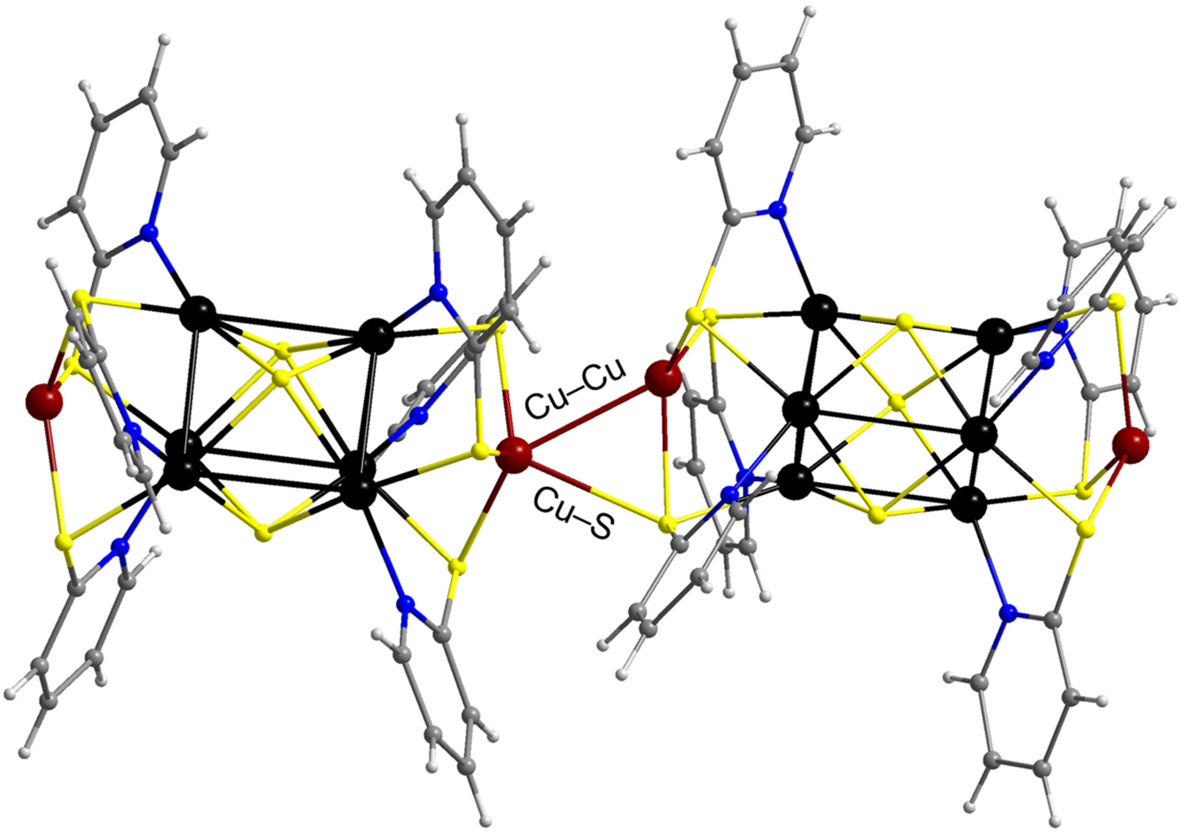

Figure S7. Two [$Cu_2Ni_6S_3(MCP)_6$] cluster units in **1** linked together via Cu–Cu and Cu–S bonds (black = Ni, dark red = Cu, yellow = S, blue = N, grey = C, white = H). The motif extends to the left and right in the same way.

a) b) c)

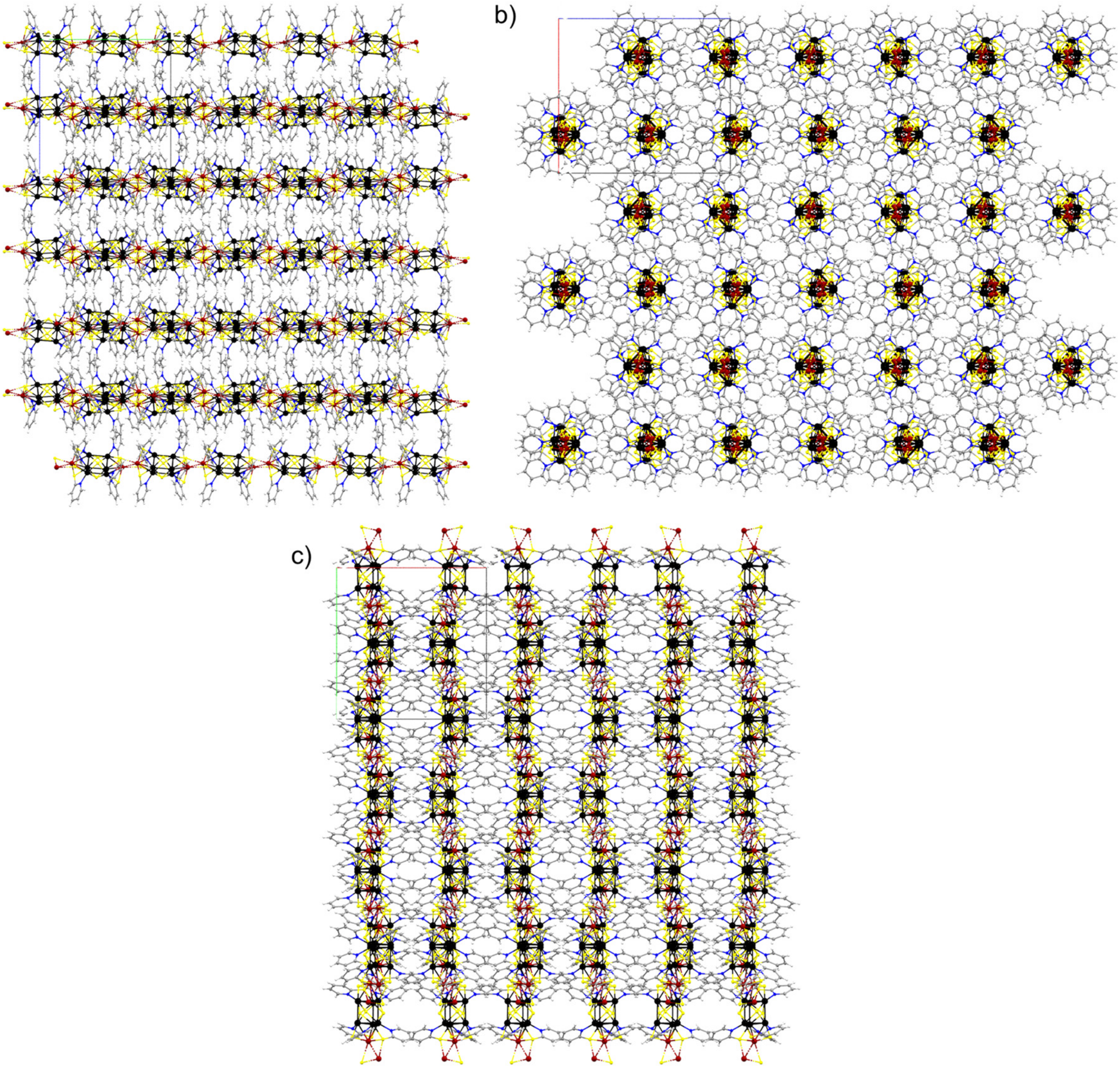

Figure S8. (2×2×2) extended supercell of clusters in **1** viewed along a) a, b) b and c) c crystallographic axis. Atom color code: black = Ni, dark red = Cu, yellow = S, blue = N, grey = C, white = H.

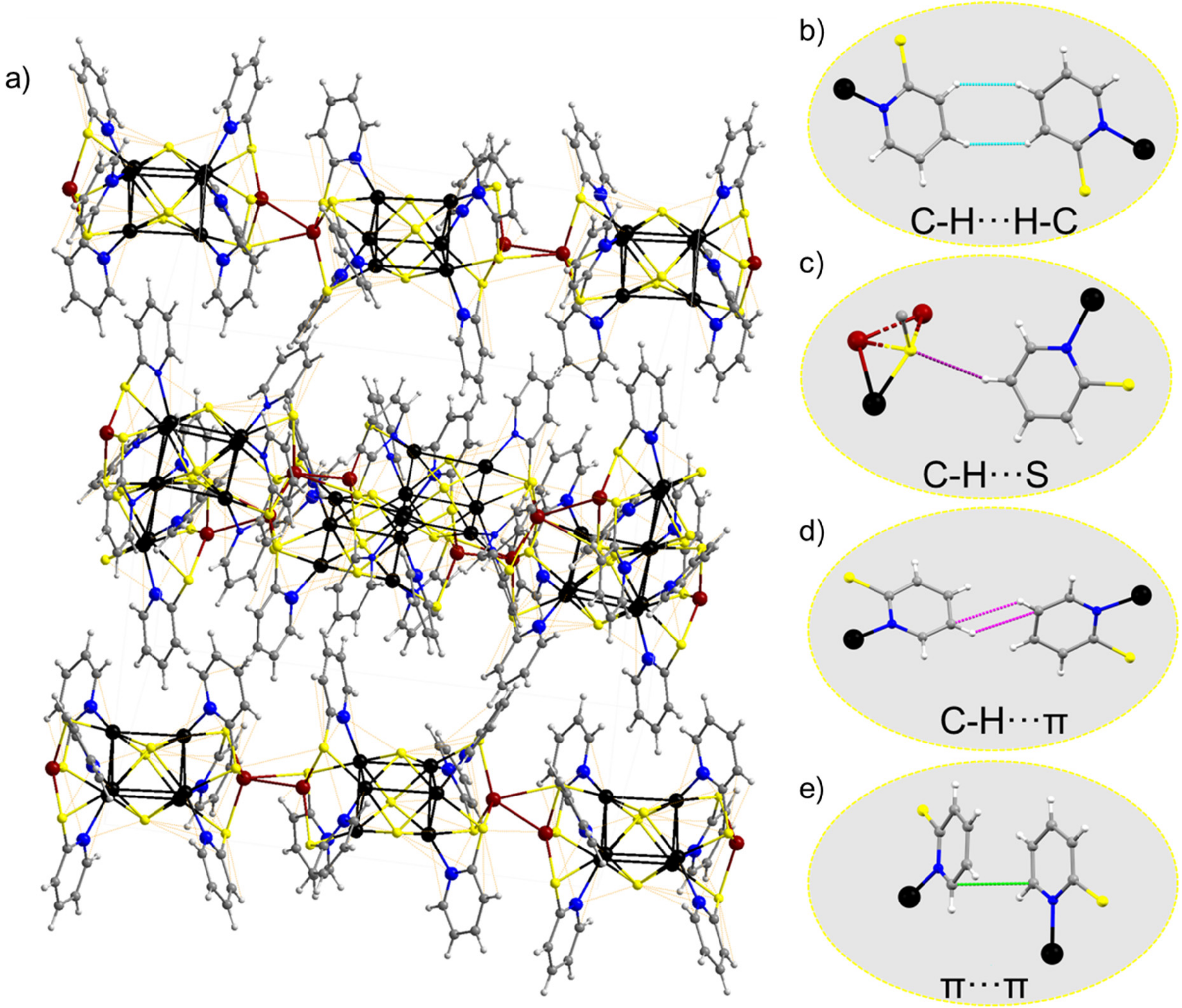

Figure S9. a) Various short contact interactions exist between the layers of the $[Cu_2Ni_6S_3(MCP)_6]_n$ clusters in **1**. For clarity, hanging contacts have been removed. Expanded views illustrate (b) C–H···H–C, (c) C–H···S, (d) C–H···π, and (e) π···π interactions occurring between the layers (black = Ni, dark red = Cu, yellow = S, blue = N, grey = C, white = H).

Table S2. Crystallographic parameters and structural refinement data for **2a**.

Identification code	$[Cu_2Ni_6(MCP)_{12}I_2]$
Empirical formula	$C_{60}H_{48}I_2N_{12}Cu_2Ni_6S_{12}$ [·0.6 C_3H_7NO]
Formula weight	2329.54
Temperature/K	150
Crystal system	Triclinic
Space group	$P\bar{1}$
a/Å	12.3680(5)
b/Å	13.3888(5)
c/Å	13.7219(4)
α/°	115.397(2)
β/°	97.655(3)
γ/°	104.303(3)
V/Å^3	1913.34(13)
Z	1
ρ_{calc}/cm^3	2.022
μ/mm^{-1}	18.766
Absorption correction type / T_{min} / T_{max}	multi-scan / 0.153 / 0.174
F(000)	1144
Crystal size/mm^3	0.12 × 0.10 × 0.10
Radiation	GaK_α (λ = 1.34143)
2θ range for data collection/°	6.45 to 126.326
Index ranges	$-15 \le h \le 16$, $-17 \le k \le 17$, $-11 \le l \le 18$
Reflections collected	36281
Independent reflections	9171 [R_{int} = 0.1110, R_{sigma} = 0.1005]
Data/restraints/parameters	9171/0/424
Goodness-of-fit on F^2	0.962
Final R indexes [$I >= 2\sigma (I)$]	R_1 = 0.0625, wR_2 = 0.1718
Final R indexes [all data]	R_1 = 0.1018, wR_2 = 0.1879
Largest diff. peak/hole /e Å^{-3}	1.324/−2.713
CCDC	2457679

A solvent mask was calculated and 27 electrons were found in a volume of 262 Å^3 in 1 void per unit cell. This is consistent with the presence of 0.3 C_3H_7NO molecules per asymmetric unit (= half a formula unit) and 0.6 C_3H_7NO molecules per unit cell, which account for 24 electrons per unit cell.

Table S3. Crystallographic parameters and structural refinement data for **2b**.

Identification code	$[Cu_2Ni_6(MCP)_{12}I_2]\cdot 2DMF$
Empirical formula	$C_{60}H_{48}I_2N_{12}Cu_2Ni_6S_{12}\cdot 2\ C_3H_7NO\ [\cdot 0.6\ C_3H_7NO]$
Formula weight	2245.01
Temperature/K	150
Crystal system	Monoclinic
Space group	$P2_1/c$
a/Å	13.8178(14)
b/Å	12.4690(16)
c/Å	24.384(3)
α/°	90
β/°	98.714(9)
γ/°	90
V/Å^3	4152.7(8)
Z	2
ρ_{calc}/cm^3	1.795
μ/mm^{-1}	16.150
Absorption correction type / T_{min} / T_{max}	multi-scan / 0.144 / 0.169
F(000)	2240
Crystal size/mm^3	0.15 × 0.14 × 0.12
Radiation	GaK$_\alpha$ (λ = 1.34143)
2θ range for data collection/°	5.63 to 123.96
Index ranges	$-9 \leq h \leq 17, -15 \leq k \leq 16, -32 \leq l \leq 30$
Reflections collected	37225
Independent reflections	9576 [R_{int} = 0.0514, R_{sigma} = 0.0481]
Data/restraints/parameters	9576/0/471
Goodness-of-fit on F^2	1.048
Final R indexes [$I >= 2\sigma (I)$]	R_1 = 0.0540, wR_2 = 0.1384
Final R indexes [all data]	R_1 = 0.0830, wR_2 = 0.1529
Largest diff. peak/hole /e Å^{-3}	1.151/−1.570
CCDC	2457680

A solvent mask was calculated and 52 electrons were found in a volume of 332 Å^3 in 2 voids per unit cell. This is consistent with the presence of 0.3 C_3H_7NO molecules per asymmetric unit (= half a formula unit) and 1.2 C_3H_7NO molecules per unit cell, which account for 48 electrons per unit cell.

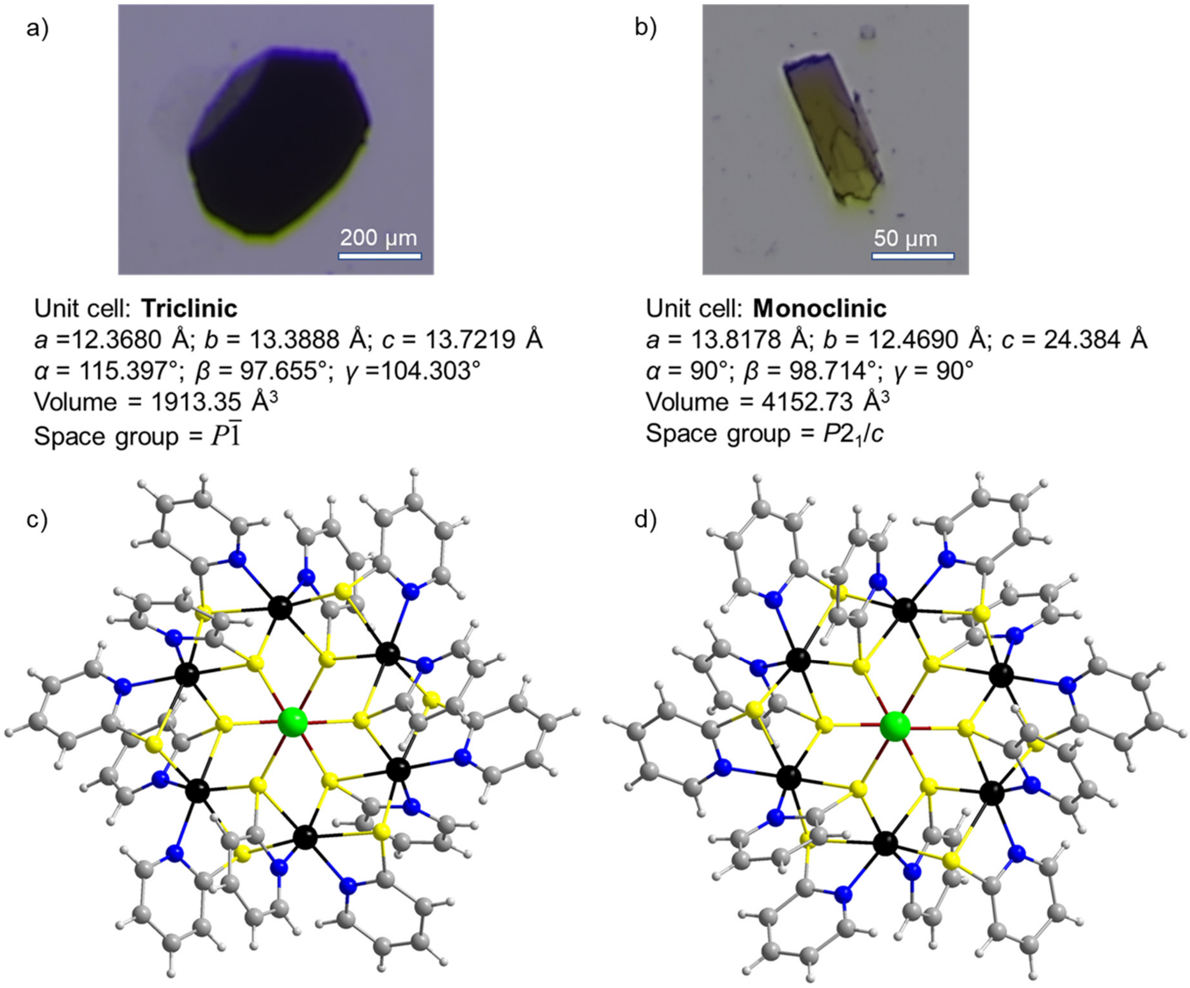

Figure S10. a) Optical photograph of a dark green colored polyhedral crystal of **2a** with unit cell parameters of the same crystal. b) Optical photograph of a green colored cuboidal crystal of **2b** with unit cell parameters of the same crystal. Identical molecular structure of the [$Cu_2Ni_6(MCP)_{12}I_2$] cluster in c) **2a** and d) **2b**. Atom color code: black = Ni, dark red = Cu, yellow = S, blue = N, grey = C, green = I, white = H.

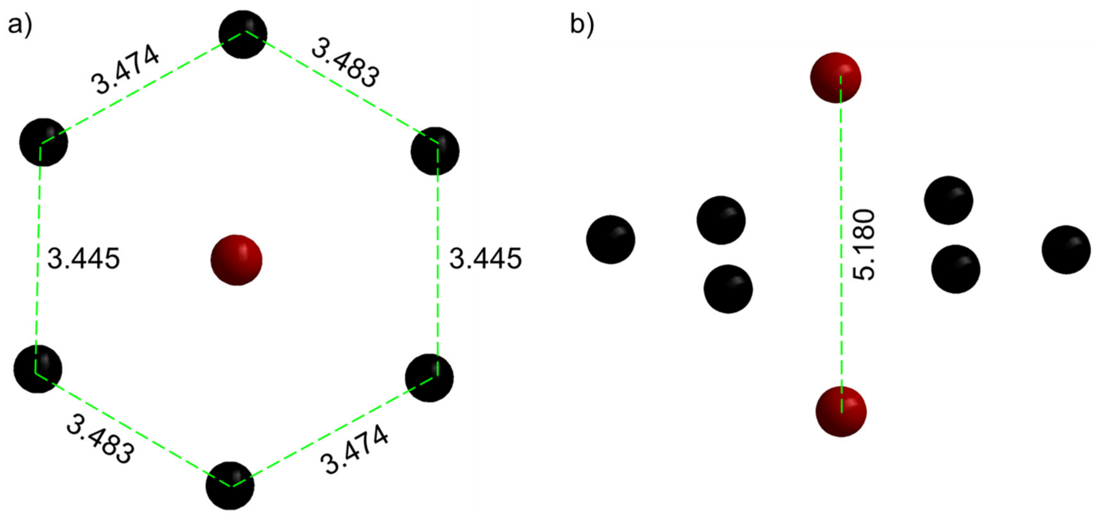

Figure S11. Interatomic Ni···Ni distances in the Ni_6 hexagonal ring suggesting the lack of intermetallic bonding in a) **2a** and b) **2b** cluster. Cu···Cu distances between two capping Cu atoms in c) **2a** and d) **2b**.

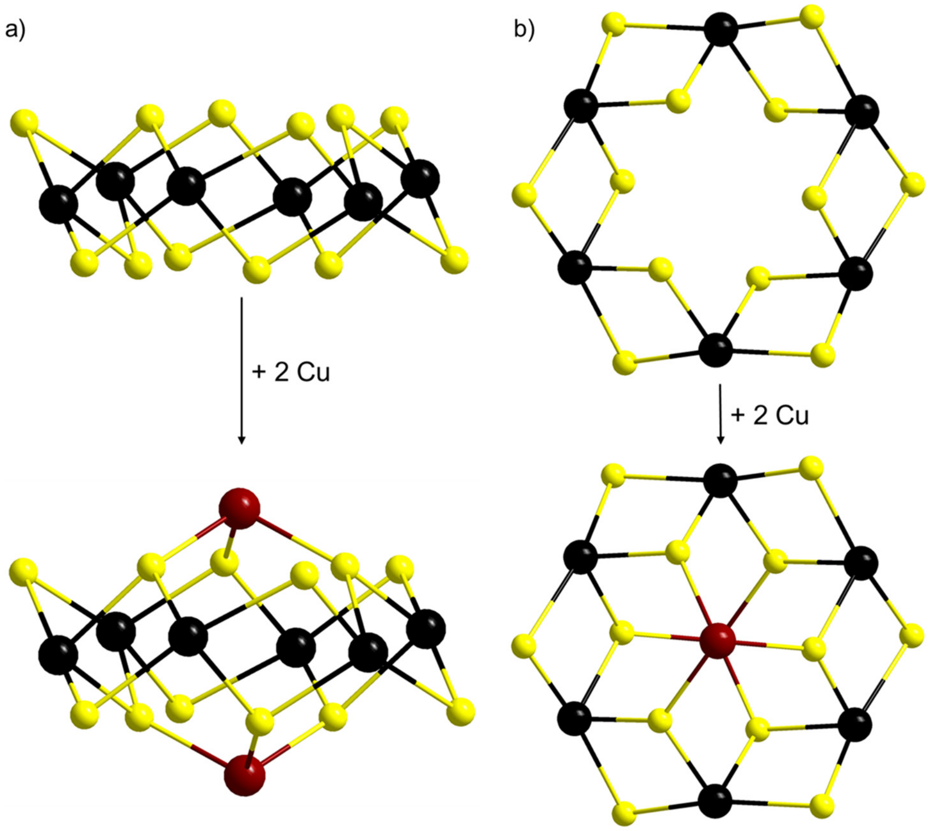

Figure S12. The tiara-like structure of the Ni_6S_{12} skeleton in the $[Cu_2Ni_6(MCP)_{12}I_2]$ cluster, as observed from (a) the side view and (b) the front view (black = Ni, dark red = Cu, yellow = S). The subsequent capping of the two Cu atoms by the Ni_6S_{12} framework is illustrated here.

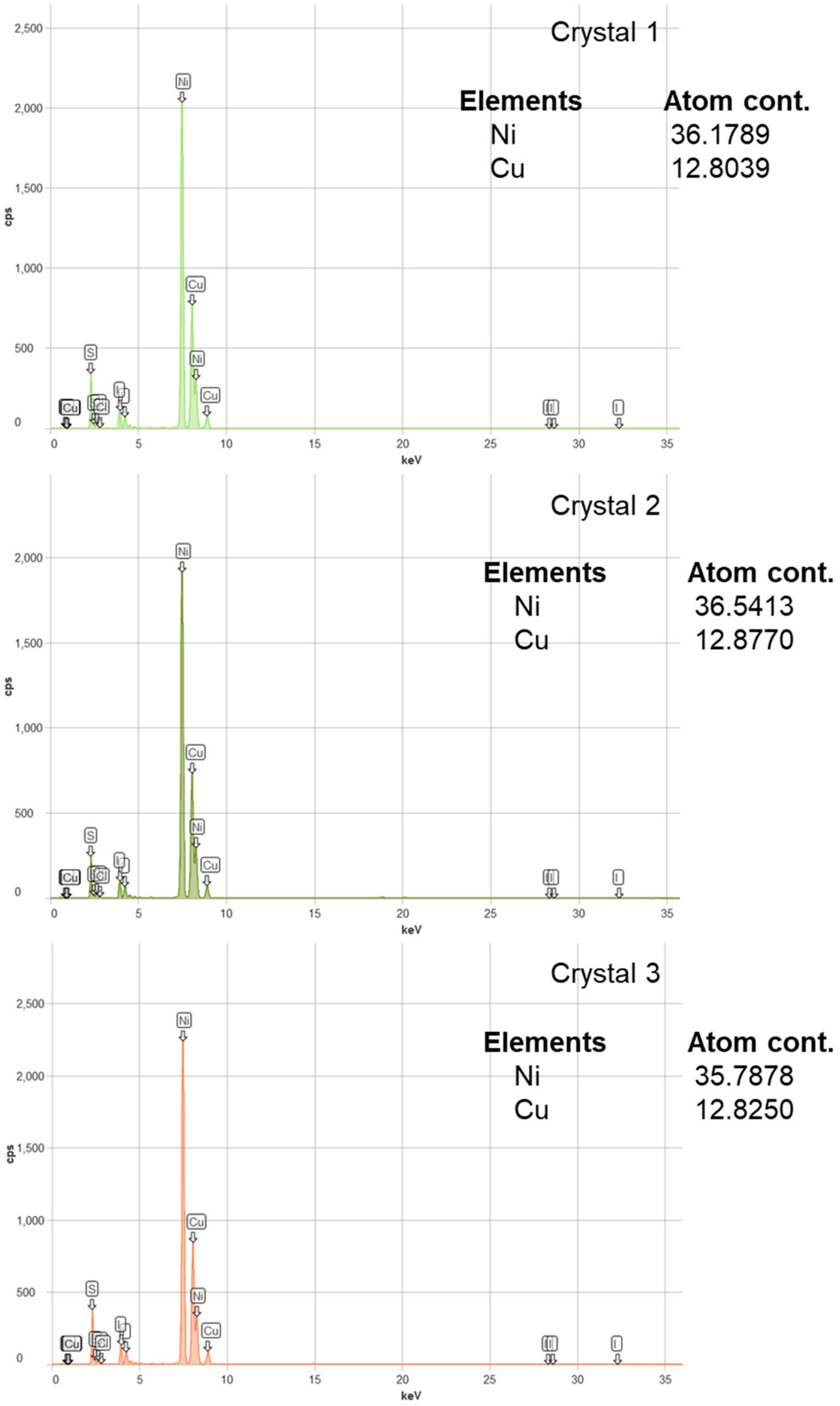

Figure S13. μ-XRF spectra of three single crystals of compound **2a**, with insets displaying the Ni and Cu content in each respective crystal.

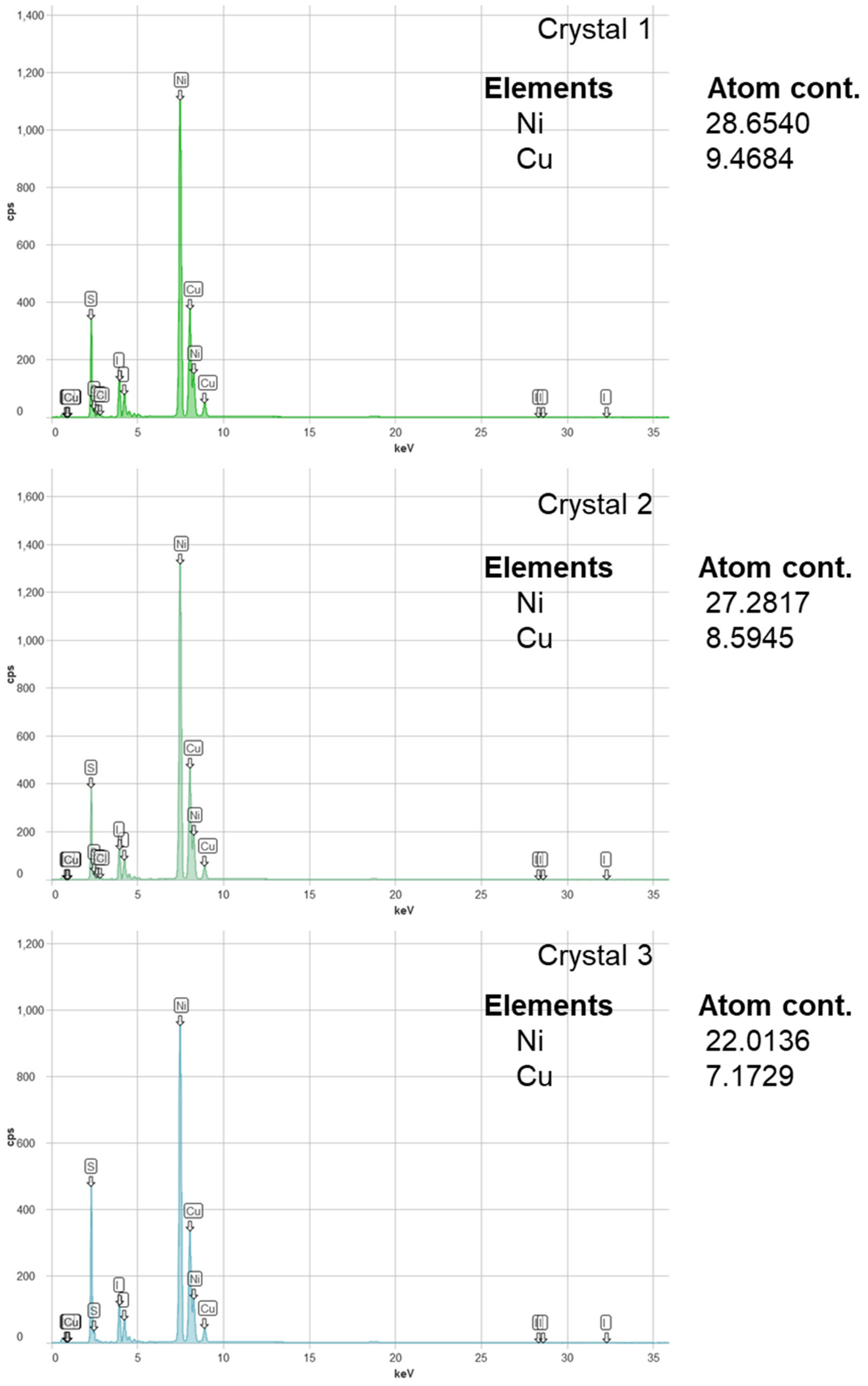

Figure S14. μ-XRF spectra of three single crystals of compound **2b**, with insets displaying the Ni and Cu content in each respective crystal.

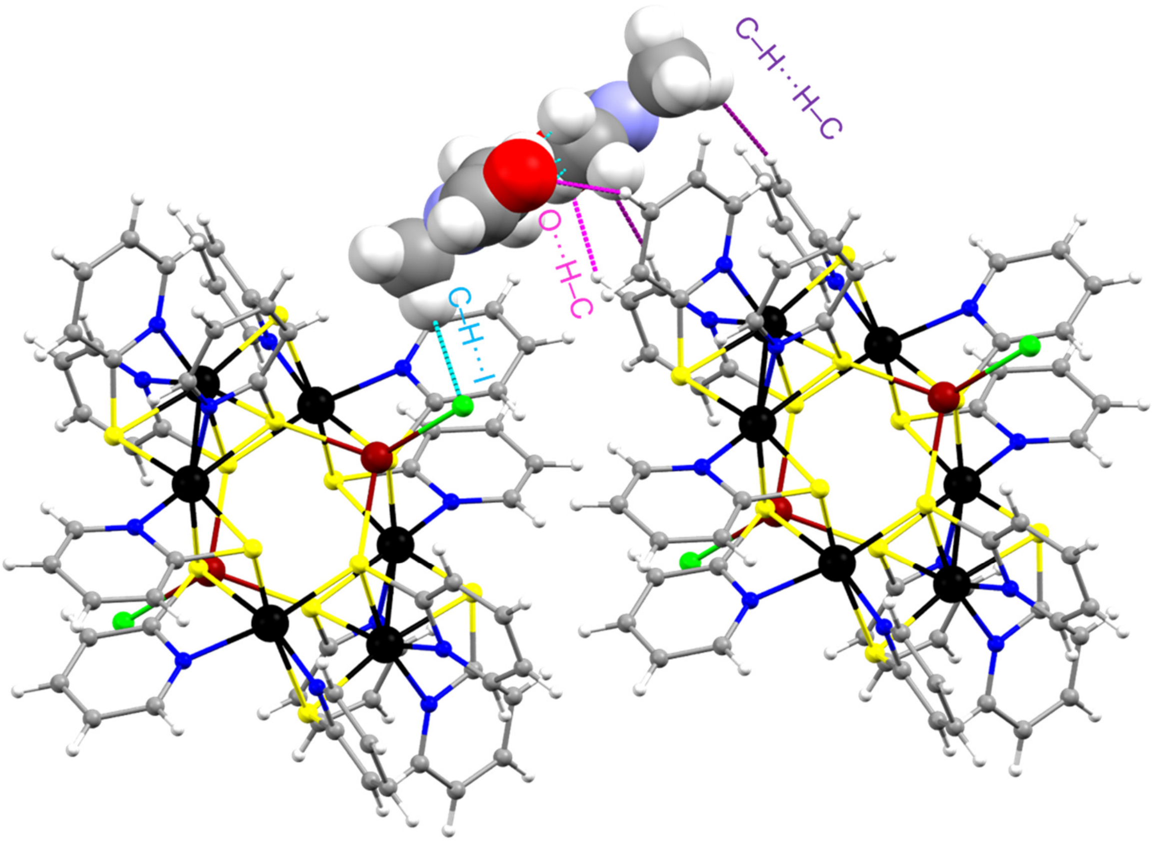

Figure S15. Various short contact intermolecular interactions between DMF and $[Cu_2Ni_6(MCP)_{12}I_2]$ cluster in **2b**. Atom color code: black = Ni, dark red = Cu, yellow = S, blue = N, red = O, grey = C, green = I, white = H.

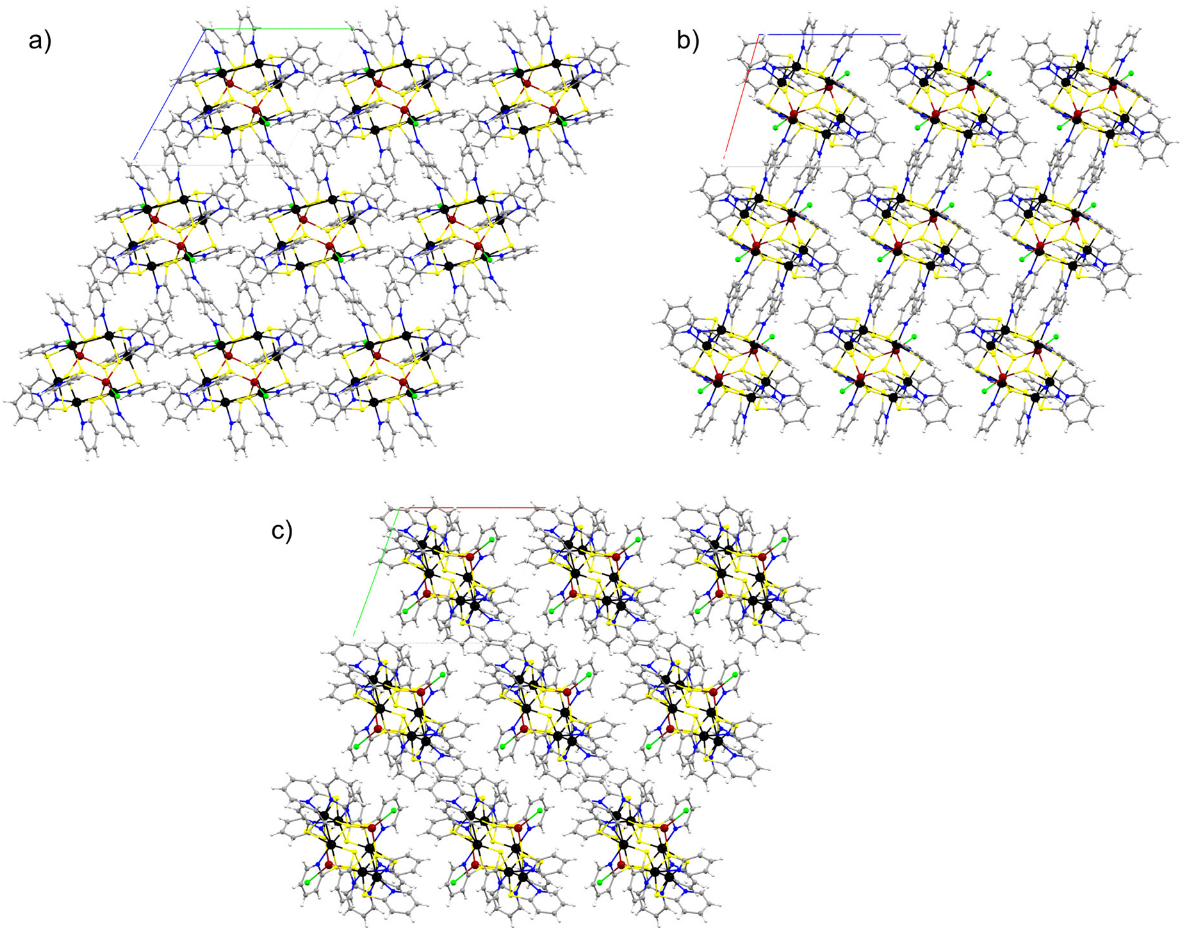

Figure S16. (3×3×3) extended packing of [$Cu_2Ni_6(MCP)_{12}I_2$] clusters in triclinic system viewed along a) a, b) b, and c) c crystallographic axis in **2a**. Atom color code: black = Ni, dark red = Cu, yellow = S, blue = N, grey = C, green = I, white = H.

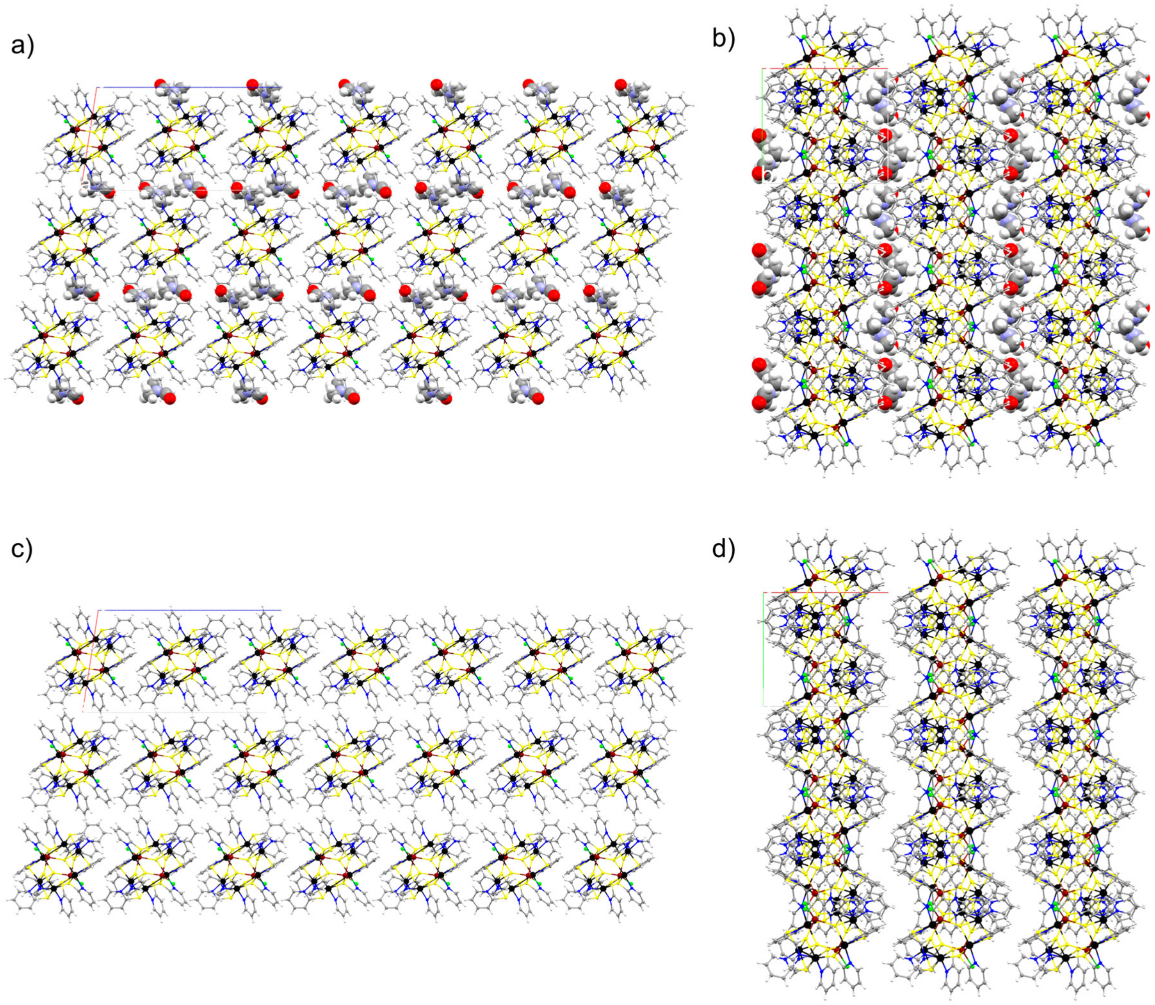

Figure S17. (3×3×3) extended packing of [$Cu_2Ni_6(MCP)_{12}I_2$] clusters in monoclinic system viewed along a, c) b and b, d) c crystallographic axis in **2b**. a, b) Packing with DMF and c and d) Packing without DMF. Atom color code: black = Ni, dark red = Cu, yellow = S, blue = N, red = O, grey = C, green = I, white = H.

4. Quantum chemical analyses

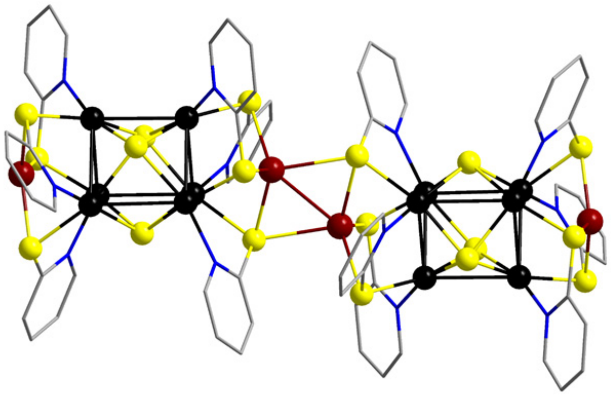

Figure S18. Computationally optimized minimum structure of a dimeric subunit of **1**. Note that the outermost Cu atoms moved inward during the geometry optimization in contrast to the experimental structure. Atom color code: black = Ni, dark red = Cu, yellow = S, blue = N, red = O, grey = C; H atoms are omitted for clarity.

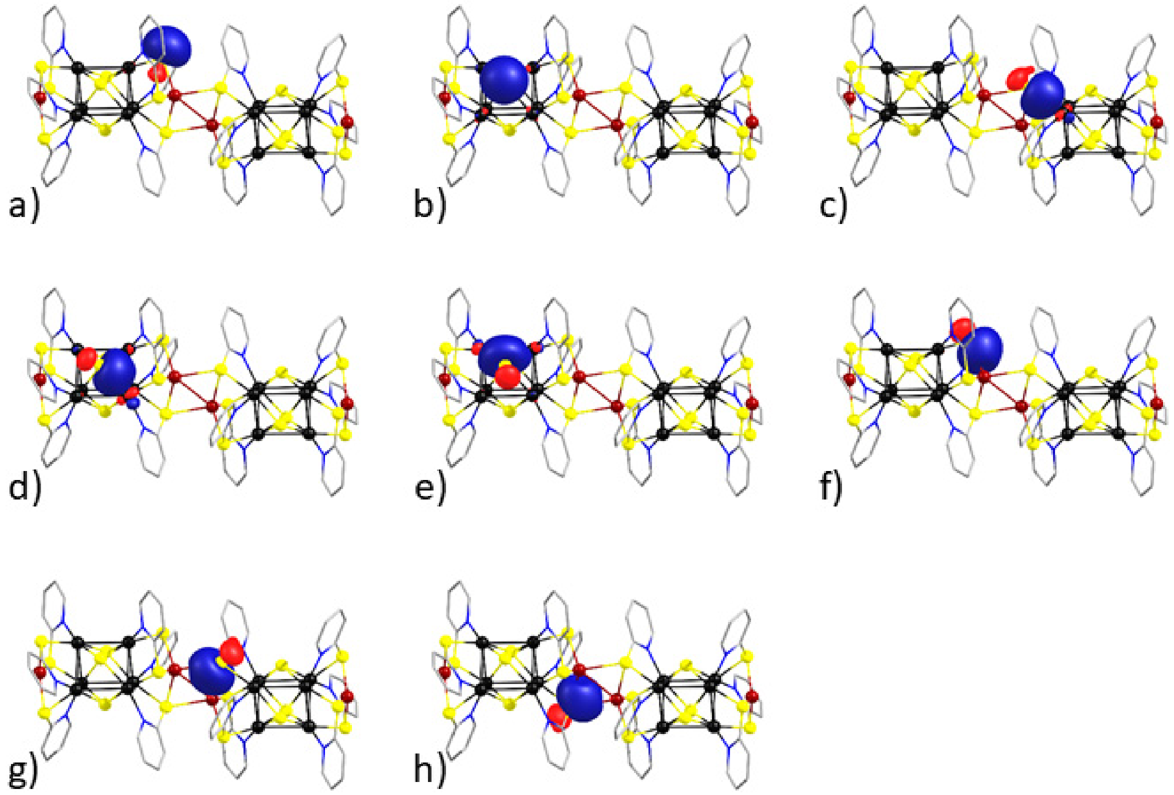

Figure S19. Representative localized molecular orbitals for a dimeric subunit of **1**: a) and b) lone-pairs at an S atom, c) and d) 2c2e Ni–S bonds, e) 3c2e Ni–S–Ni bond, f) 2c2e Cu–S bond, g) and h) 3c2e Cu–S–Cu bonds. Atom color code: black = Ni, dark red = Cu, yellow = S, blue = N, red = O, grey = C; H atoms are omitted for clarity.

Table S4. Comparison of experimental and calculated bond length ranges in **1**. Deviations marked with an * are due to **1** being treated as a dimer instead as a polymer during calculations.

	Experimental / Å	Calculated / Å
Ni–Ni	2.689 – 2.857	2.721 – 2.810
Cu–Cu	2.767	2.763
Ni–S	2.195 – 2.304	2.197 – 2.281*
Cu–S	2.293 – 2.380	2.319 – 2.560*
Ni···Cu	2.741 – 2.897	2.655* – 2.914

Table S5. Partial charges for Ni and Cu atoms in **1** obtained by Mulliken and natural population analyses (NPA). Values marked with an * are due to **1** being treated as a dimer instead as a polymer during calculations.

	Mulliken	NPA
Ni	0.1	0.5
Cu	0.0* – 0.3	0.7

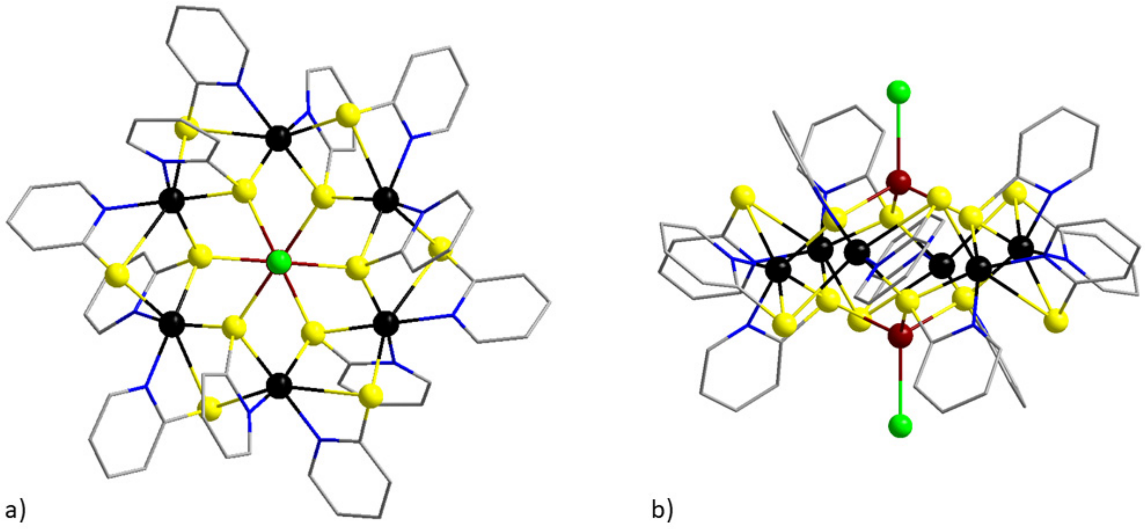

Figure S20. Computationally optimized minimum structure of **2**, a) top-view, b) side-view. Atom color code: black = Ni, dark red = Cu, yellow = S, blue = N, red = O, grey = C, green = I; H atoms are omitted for clarity.

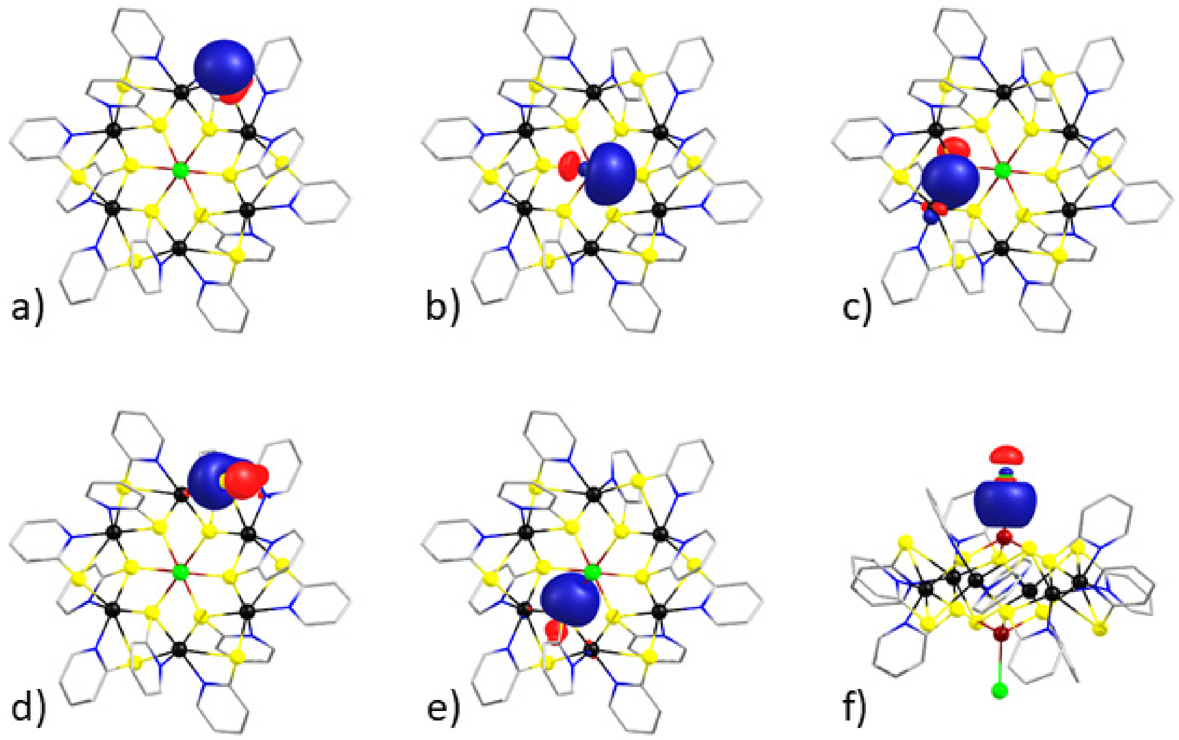

Figure S21. Representative LMOs of **2**: a) lone-pair at an S atom, b) lone-pair at an I atom, c) and d) 2c2e Ni–S bonds, e) 2c2e Cu–S bond, f) Cu–I bond (side-view). Atom color code: black = Ni, dark red = Cu, yellow = S, blue = N, red = O, grey = C, green = I; H atoms are omitted for clarity.

Table S6. Comparison of experimental and calculated bond length ranges in **2**.

	Experimental / Å	Calculated / Å
Ni–S	2.400 – 2.555	2.244 – 3.002
Cu–S	2.348 – 2.368	2.431 – 2.433

Table S7. Partial charges for Ni and Cu atoms in **1** obtained by Mulliken and natural population analyses (NPA).

	Mulliken	NPA
Ni	0.2	0.9
Cu	−0.1	0.7

5. High-resolution mass spectrometry, vibrational, and optical spectroscopy

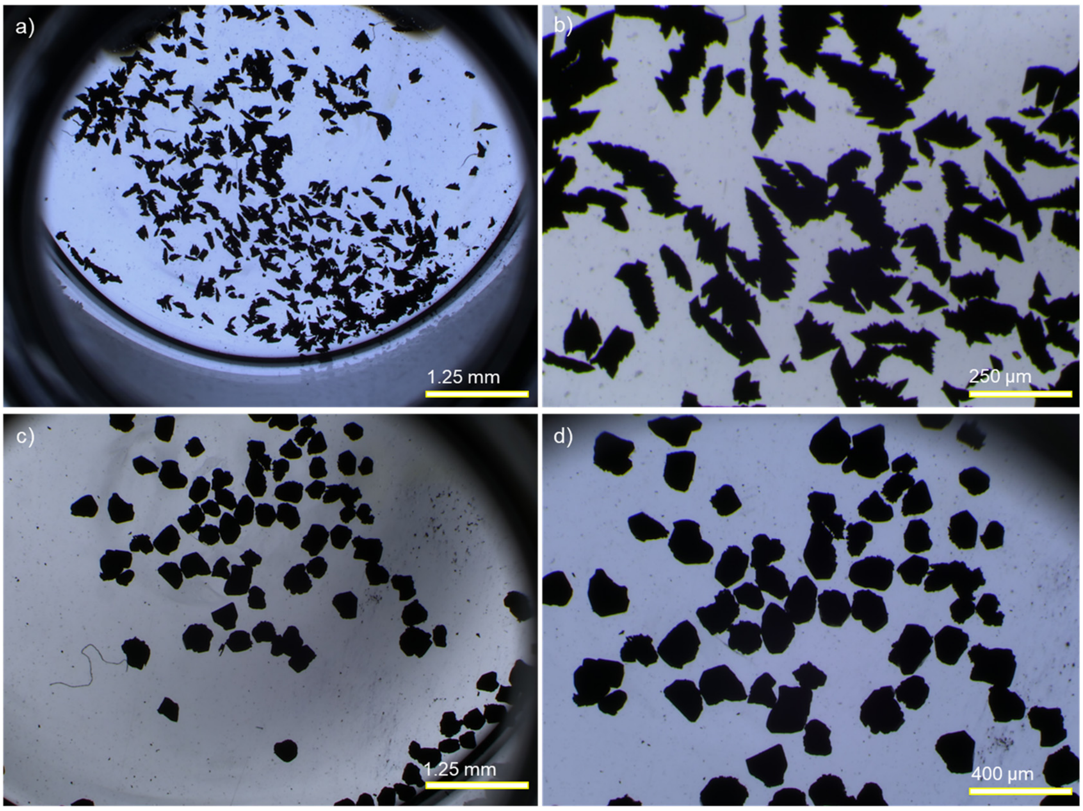

Figure S22. Optical photographs of the manually collected single crystals: a) and b) for **1** as well as c) and d) for **2a**. b) and d) are the magnified views of the optical micrographs.

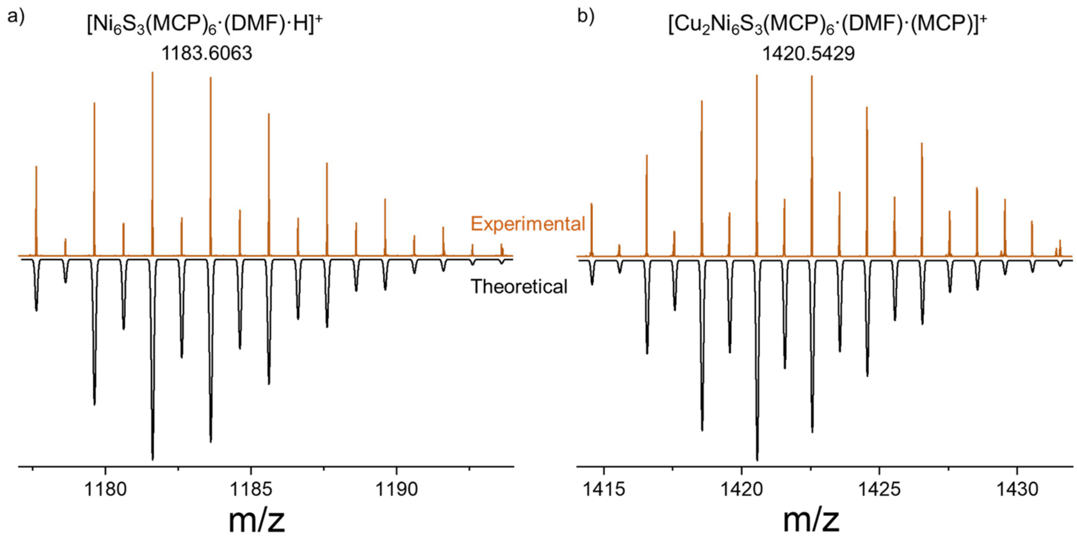

Figure S23. High-resolution mass spectra show the isotopic distribution of the experimental spectrum in comparison with the theoretical pattern of a) *m/z* 1183.6063 and b) *m/z* 1420.5429 peak for compound **1**.

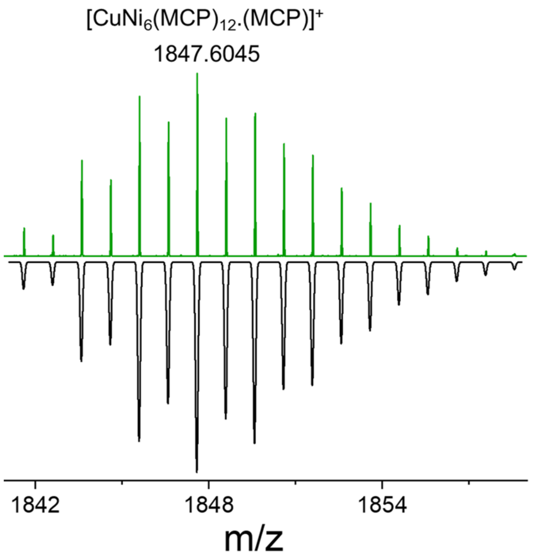

Figure S24. High-resolution mass spectra show the isotopic distribution of the experimental spectrum in comparison with the theoretical pattern of *m/z* 1847.6045 peak for compound **2**.

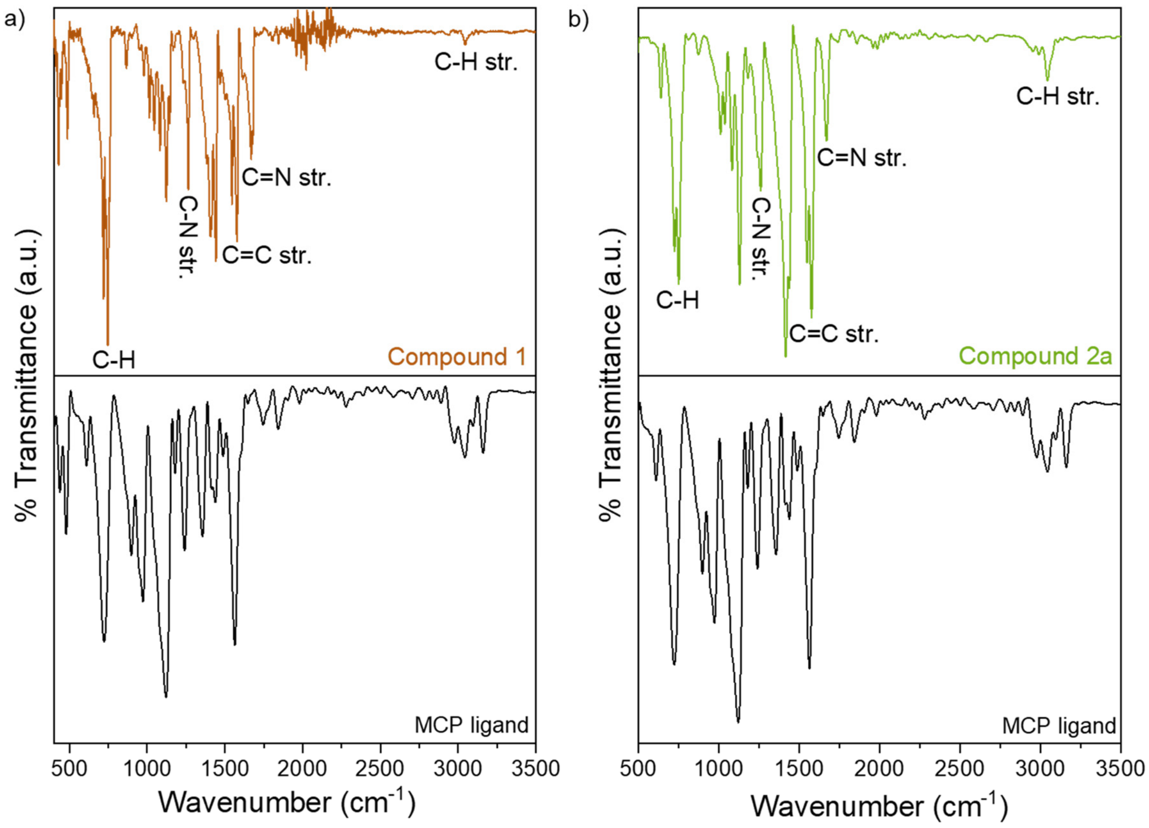

Figure S25. Comparative FT-IR spectra of a) **1** and b) **2a** analyzed alongside the MCP ligand. Each spectrum was recorded in ATR mode using selected single crystals of the respective clusters and ligand.

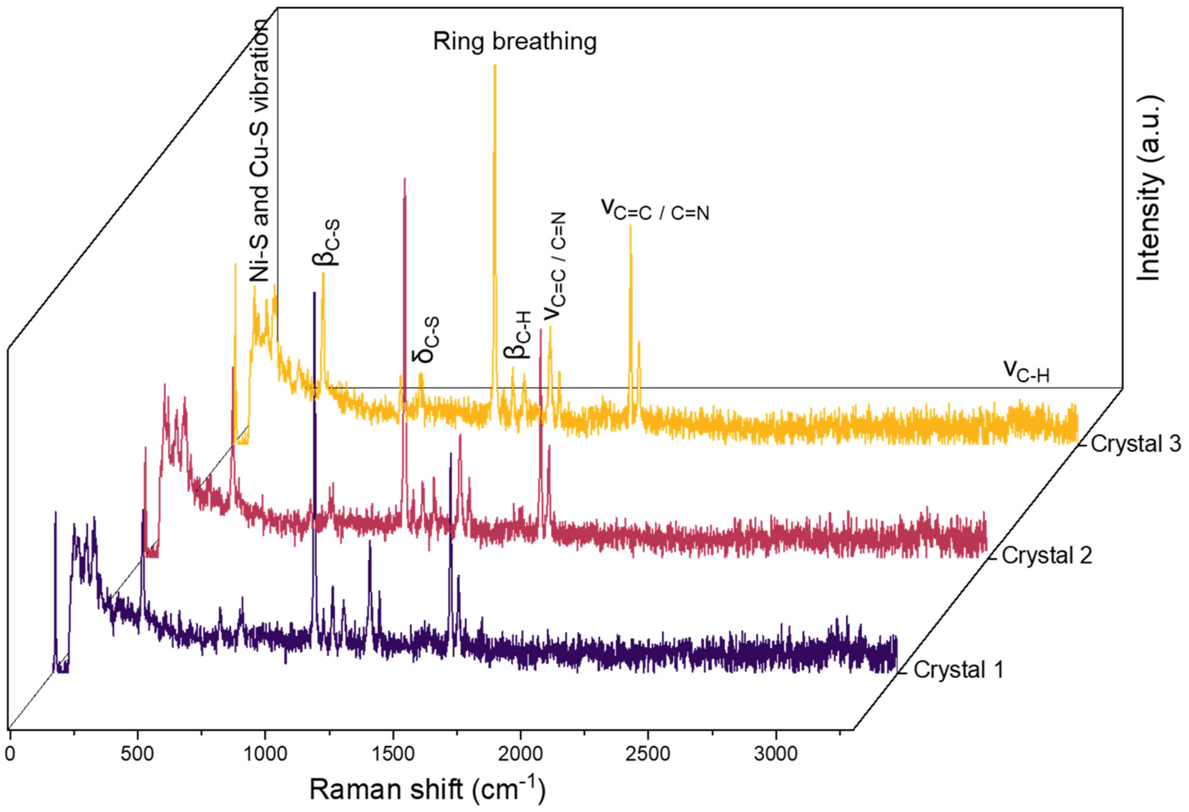

Figure S26. Comparative Raman spectra of three single crystals of **1**. A 532 nm laser with a power of 0.5 mW was employed for the measurements. The spectral average of three scans is used for each crystal.

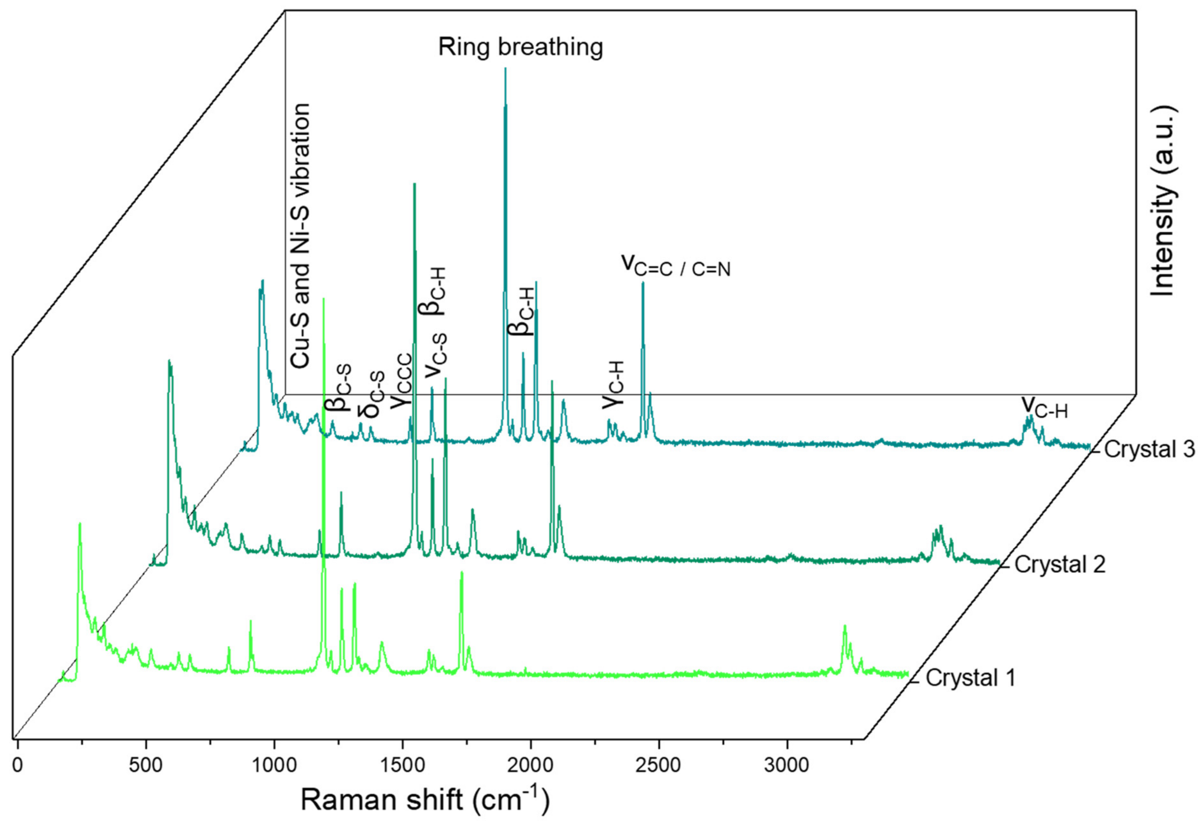

Figure S27. Comparative Raman spectra of three single crystals of **2a**. A 532 nm laser with a power of 0.5 mW was employed for the measurements. The spectral average of three scans is used for each crystal.

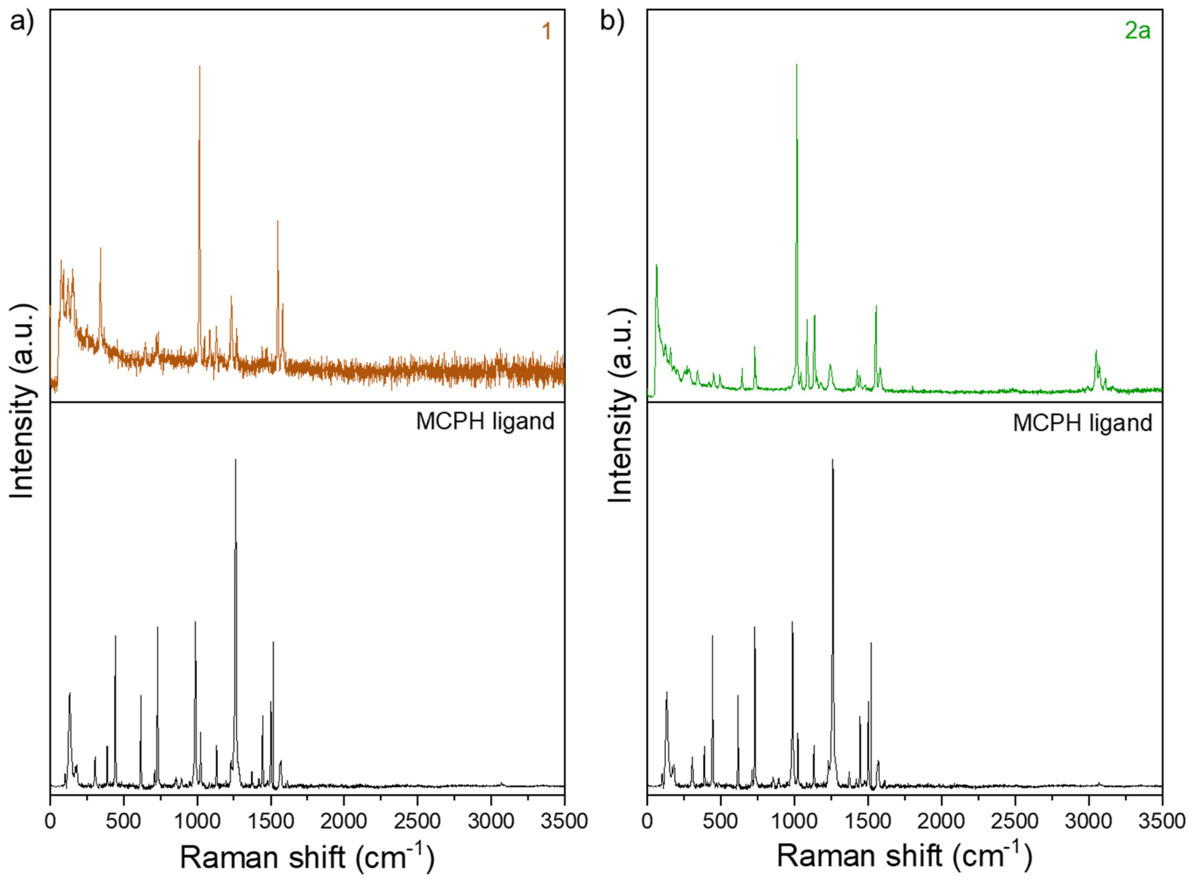

Figure S28. Comparative Raman spectra of a) **1**, b) **2a** and the MCP ligand. A 785 nm laser (laser power 0.5 mW) was utilized for the spectral measurement of MCP crystals, while a 532 nm laser with a power of 0.5 mW was employed for the spectral measurements of the cluster crystals.

Table S8. Assignment of the Raman vibrational bands for **1**, **2a** and the MCP ligand, with all Raman values expressed in cm^{-1}.

No.	MCP ligand	**1**	**2a**	Assignments
1.	3067	3059	3111, 3068, 3052, 3040	$\nu_{C–H}$
2.	1569, 1501, 1445,	1580, 1548	1580, 1553	$\nu_{C=C}$ and $\nu_{C=N}$
3.	1419,1371		1420, 1444	$\gamma_{C–H}$
4.	1261,	1269, 1233	1242, 1183	$\nu_{C=C}$ and $\nu_{C=N}$
5.	1132	1131, 1086	1136, 1086, 1044,	Ring breathing and $\beta_{C–H}$
6.	1023, 987	1015	1016	Ring breathing
7.	894, 856			
8.	730, 710,	724	730	$\nu_{C–S}$ and $\beta_{C–H}$
9.	616	646	646	γ_{CCC}
10.	443		490, 452,	$\delta_{C–S}$
11.	388, 306	343	342, 280	$\beta_{C–S}$
12.	180, 170, 132	153,	184, 157	
13		123, 91, 74	255, 204, 123 98, 70, 60	Ni–Ni and Ni–Cu, Ni–S, Cu–S vibrations

Legend for the symbols: ν = stretching; β = bending, δ = in plane deformation, γ = out of plane deformation.

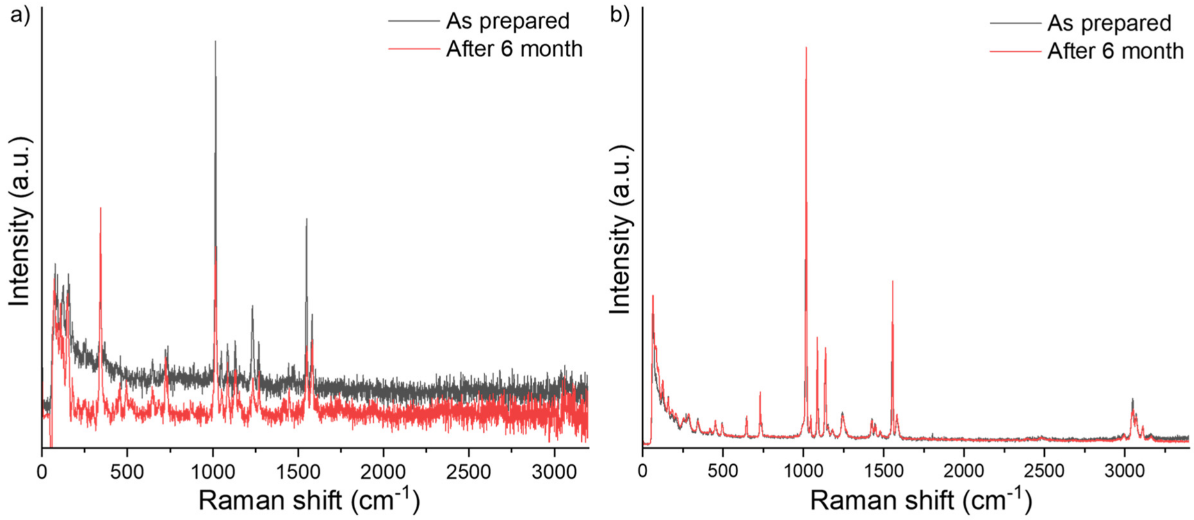

Figure S29. Comparative Raman spectra of a) compound **1** and compound b) **2a**, both as prepared and six months after their synthesis and storage under ambient conditions.

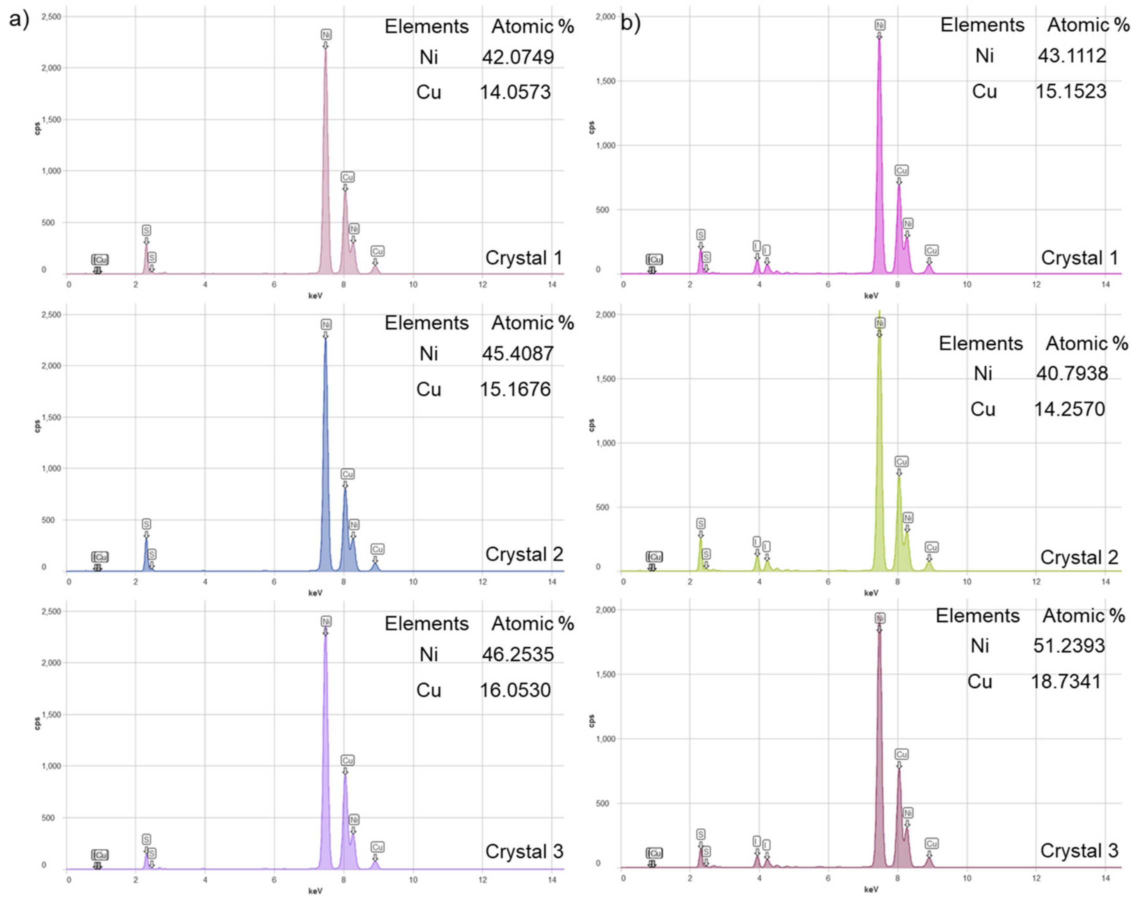

Figure S30. μ-XRF spectra of compounds a) **1** and b) **2a**, recorded six months after their synthesis and storage under ambient conditions.

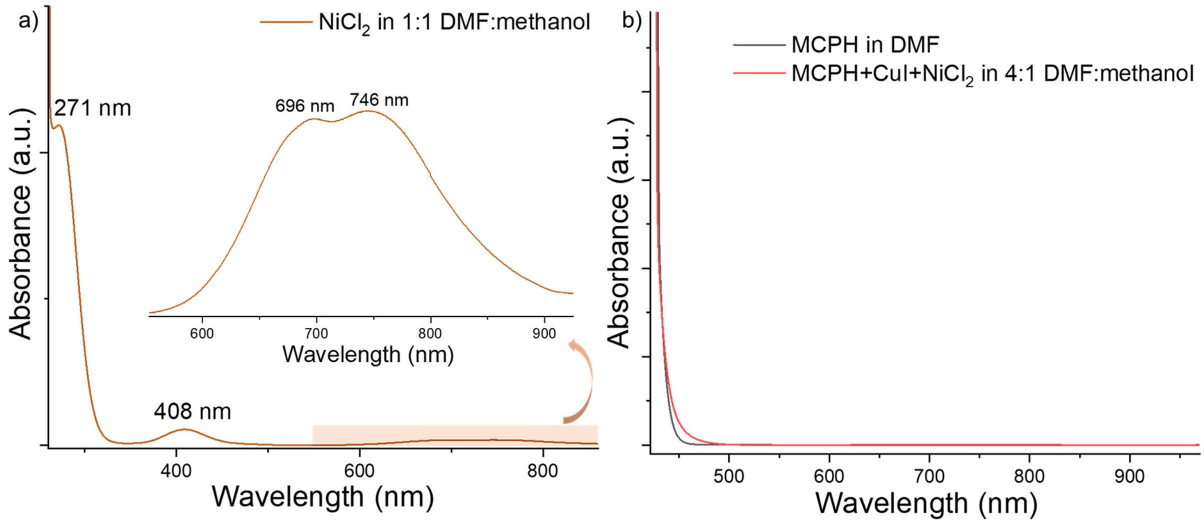

Figure S31. a) UV-vis absorption spectrum of $NiCl_2$ in 1:1 DMF: methanol. The inset shows a zoom into the highlighted region. b) Absorption spectrum of MCPH as compared to a spectrum of the mixture of all precursors in the respective solvent system.

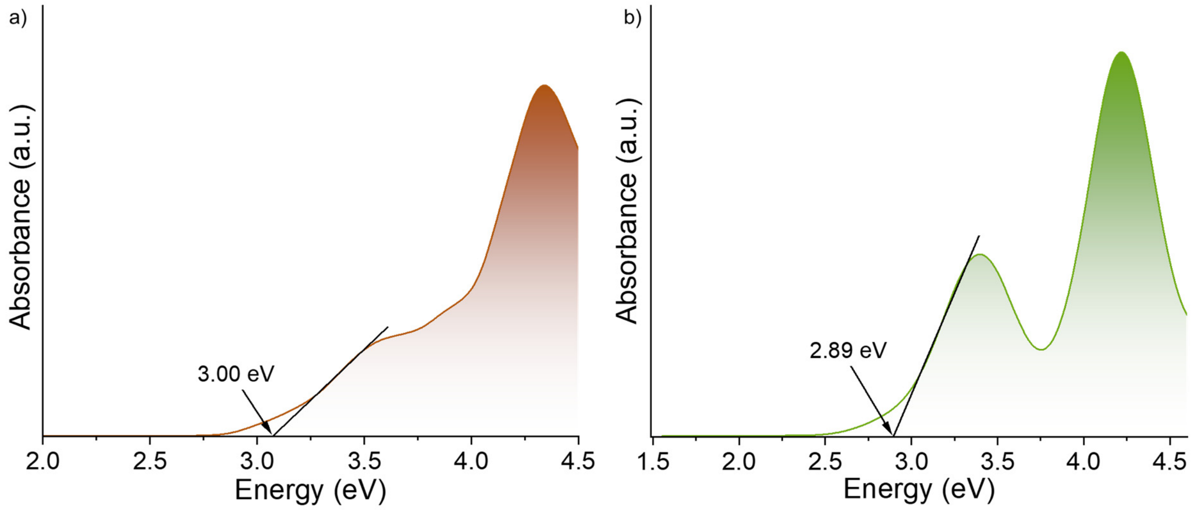

Figure S32. UV-vis optical absorption spectra of a DMF solution of a) **1** and b) **2a** to determine the optical bandgap of the compounds.

6. Analyses of the magnetic properties

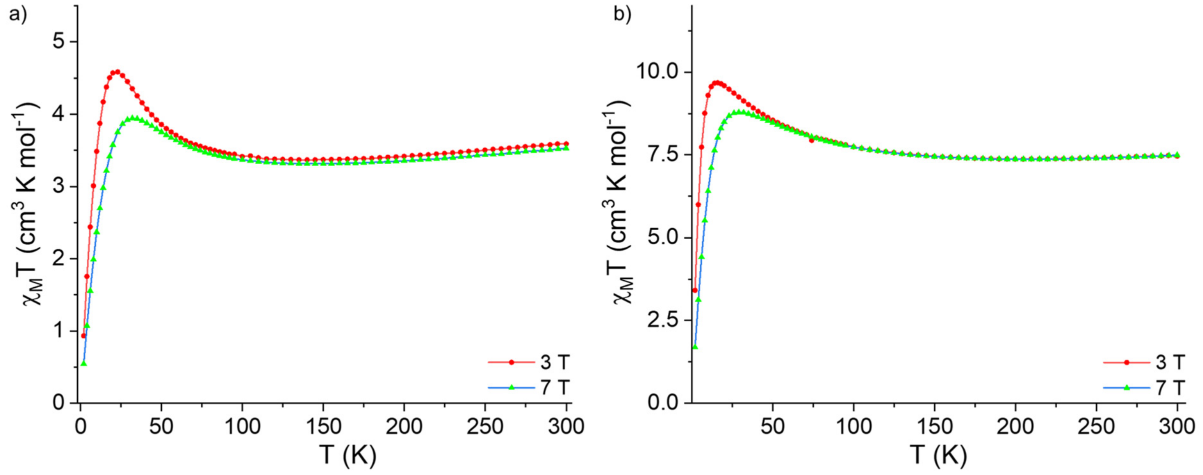

Figure S33. Comparative analysis of χ_mT for a) compound **1** and b) compound **2a** over a temperature range from 300 to 2 K, under applied magnetic fields of 3, and 7 T, solid lines are a guide to the eye.

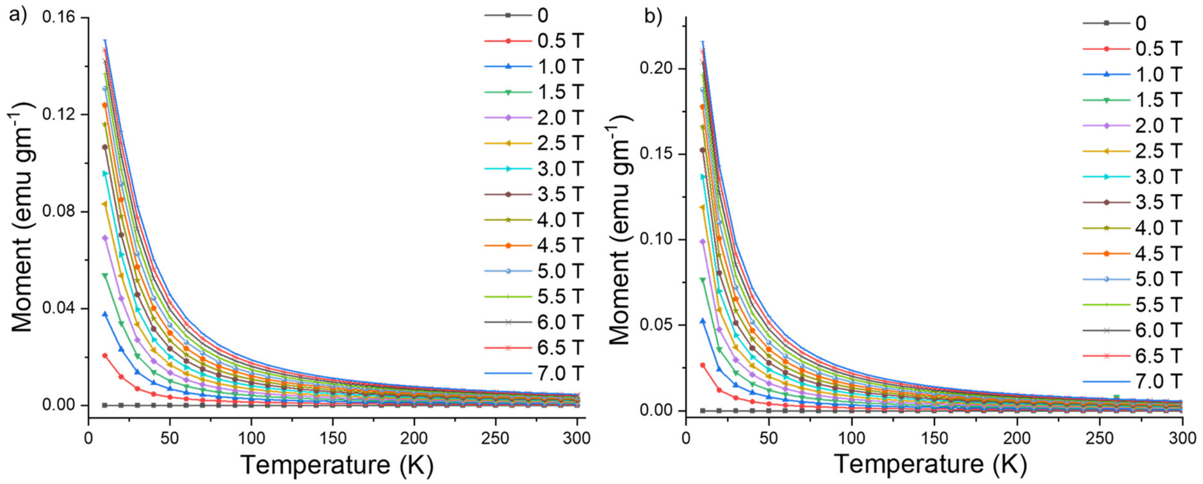

Figure S34. Field-dependent magnetization of compounds a) **1** and b) **2a** as the temperature decreases from 300 K to 10 K, solid lines are a guide to the eye.

Table S9. Summary of solvothermal reactions under variation of the stoichiometry of the precursors ($NiCl_2$:CuI) and the temperature. All reactions were carried out in a mixture of DMF and methanol (4:1, v/v).

No.	$NiCl_2$	CuI	Ni:Cu	MCPH	T (time)	Observation
1.	15.5 mg (120 mM)	6.5 mg (35 mM)	3.43:1	30 mg (270 mM)	120 °C (72 h)	Mixture of **1**, **2a**, **2b** and **A** crystals
2.	15.5 mg (120 mM)	26 mg (140 mM)	0.86:1	30 mg (270 mM)	120 °C (72 h)	Mixture of **1**, **2a** and **A** crystals, minor amount of **2b** crystals
3.	15.5 mg (120 mM)	13 mg (70 mM)	1.71:1	30 mg (270 mM)	120 °C (72 h)	Mixture of **1** and **2a** crystals, minor quantity of **A** and **2b** crystals
4.	31 mg (240 mM)	13 mg (70 mM)	3.43:1	30 mg (270 mM)	120 °C (72 h)	Mixture of **1**, **2a**, **2b** and **A** crystals**,** also some amorphous precipitates
5.	15.5 mg (120 mM)	6.5 mg (35 mM)	3.43:1	30 mg (270 mM)	100 °C (72 h)	Mixture of **2a**, **2b** and **A** crystals, minor amount of **1** crystals
6.	15.5 mg (120 mM)	26 mg (140 mM)	0.86:1	30 mg (270 mM)	100 °C (72 h)	Major amount of **A** crystals and minor amount of **1**, **2a** and **2b**
7.	15.5 mg (120 mM)	13 mg (70 mM)	1.71:1	30 mg (270 mM)	100 °C (72 h)	Mixture of **1**, **2a**, **2b** and **A** crystals
8.	31 mg (240 mM)	13 mg (70 mM)	3.43:1	30 mg (270 mM)	100 °C (72 h)	Mostly **2a** crystals, minor amount of **1**, **2b** and **A** crystals
9.	15.5 mg (120 mM)	6.5 mg (35 mM)	3.43:1	30 mg (270 mM)	80 °C (72 h)	Mostly **2a** and **2b** crystals, no **1** and **A** crystals
10.	15.5 mg (120 mM)	26 mg (140 mM)	0.86:1	30 mg (270 mM)	80 °C (72 h)	Mostly **A** crystals, a few **2a** and **2b** crystals
11.	15.5 mg (120 mM)	13 mg (70 mM)	1.71:1	30 mg (270 mM)	80 °C (72 h)	Mostly **2a**, **2b** and **A** crystals, no **1** crystal
12.	31 mg (240 mM)	13 mg (70 mM)	3.43:1	30 mg (270 mM)	80 °C (72 h)	Mostly **2a** and **2b** crystals, no **1** and **A** crystals

7. References

[1] TURBOMOLE V7.8 2024, a development of University of Karlsruhe and Forschungszentrum Karlsruhe GmbH, 1989–2007, TURBOMOLE GmbH, since 2007; available from http://www.turbomole.com. S. G. Balasubramani, G. P. Chen, S. Coriani, M. Diedenhofen, M. S. Frank, Y. J. Franzke, F. Furche, R. Grotjahn, M. E. Harding, C. Hättig, A. Hellweg, B. Helmich-Paris, C. Holzer, U. Huniar, M. Kaupp, A. Marefat Khah, S. Karbalaei Khani, T. Müller, F. Mack, B. D. Nguyen, S. M. Parker, E. Perlt, D. Rappoport, K. Reiter, S. Roy, M. Rückert, G. Schmitz, M. Sierka, E. Tapavicza, D. P. Tew, C. van Wüllen, V. K. Voora, F. Weigend, A. Wodyński, J. M. Yu, *J. Chem. Phys.* **2020**, *152* (18), 184107.

[2] a) J. P Perdew, K. Burke, M. Ernzerhof, *Phys. Rev. Lett.* **1996**, *77*, 3865–3868. b) C. Adamo, V. Barone, *J. Chem. Phys.*, **1999**, *110*, 6158–6170.

[3] F. Weigend, R. Ahlrichs, *Phys. Chem. Chem. Phys.* **2005**, *7* (18), 3297–3305.

[4] a) K. Eichkorn, O. Treutler, H. Öhm, M. Häser, R. Ahlrichs, *Chem. Phys. Lett.* **1995**, *240*, 283–290. b) K. Eichkorn, F. Weigend, O. Treutler, R. Ahlrichs, *Theor. Chem. Acc.* **1997**, *97*, 119–124.

[5] F. Weigend, *Chem. Chem. Phys.* **2005**, *8* (9), 1057–1065.

[6] R. S. Mulliken, *J. Chem. Phys.* **1955**, *23*, 2338–2342.

[7] A. E. Reed, R. B. Weinstock, F. Weinhold, *J. Chem. Phys.* **1985**, *83*, 735–746.

[9] S. F. Boys, *in Quantum Theory of Atoms, Molecules and the Solid State* (Ed.: P.-O. Löwdin), New York, **1966**, 253–262.

[10] a) P. Deglmann, F. Furche, R. Ahlrichs, *Chem. Phys. Lett.* **2002**, *362*, 511–518. b) P. Deglmann, F. Furche, *J. Chem. Phys.* **2002**, *117*, 9535–9538. c) P. Deglmann, K. May, F. Furche, R. Ahlrichs, *Chem. Phys. Lett.* **2004**, *384*, 103–107.
