# Supplementary material for: Bonding Features and Magnetic Ordering in Thiolate‐Bridged Copper‐Nickel Clusters Synthesized at Elevated Temperature
Source: Small. 2025 Aug 8;21(38):e06920. doi: 10.1002/smll.202506920 (PMC12462566; doi:10.1002/smll.202506920)

checkCIF/PLATON report

Structure factors have been supplied for datablock(s) 1

THIS REPORT IS FOR GUIDANCE ONLY. IF USED AS PART OF A REVIEW PROCEDURE FOR PUBLICATION, IT SHOULD NOT REPLACE THE EXPERTISE OF AN EXPERIENCED CRYSTALLOGRAPHIC REFEREE.

No syntax errors found. CIF dictionary Interpreting this report

Datablock: 1

Bond precision:	C-C = 0.0058 A	Wavelength=1.34143	
Cell:	a=19.2770(18)	b=19.490(2)	c=21.376(3)
	alpha=90	beta=90	gamma=90
Temperature:	150 K		

	Calculated	Reported
Volume	8031.2(16)	8031.2(15)
Space group	P b c n	P b c n
Hall group	-P 2n 2ab	-P 2n 2ab
Moiety formula	C30 H24 Cu2 N6 Ni6 S9 [+ solvent]	C30 H24 Cu2 N6 Ni6 S9, 0.6[C3H7NO]
Sum formula	C30 H24 Cu2 N6 Ni6 S9 [+ solvent]	C31.80 H28.20 Cu2 N6.60 Ni6 O0.60 S9
Mr	1236.33	1280.29
Dx,g cm-3	2.045	2.118
Z	8	8
Mu (mm-1)	23.746	23.775
F000	4928.0	5120.0
F000'	4849.79	
h,k,lmax	25,25,28	25,24,28
Nref	9853	9386
Tmin,Tmax	0.110,0.305	0.110,0.305
Tmin'	0.005	

Correction method= # Reported T Limits: Tmin=0.110 Tmax=0.305
AbsCorr = MULTI-SCAN

Data completeness= 0.953 Theta(max)= 62.954

R(reflections)= 0.0398(5934)

wR2(reflections)= 0.0819(9386)

S = 0.907 Npar= 478

The following ALERTS were generated. Each ALERT has the format
test-name_ALERT_alert-type_alert-level.
Click on the hyperlinks for more details of the test.

Alert level C

```
ABSTY02_ALERT_1_C  An _exptl_absorpt_correction_type has been given without
            a literature citation. This should be contained in the
            _exptl_absorpt_process_details field.
            Absorption correction given as multi-scan
PLAT767_ALERT_4_C INS Embedded LIST 6 Instruction Should be LIST 4     Please Check
PLAT905_ALERT_3_C Negative K value in the Analysis of Variance ...     -0.196 Report
PLAT911_ALERT_3_C Missing FCF Refl Between Thmin & STh/L=    0.600         16 Report
                6 22  0,  10 16  0,   8 17  1,  10 16  1,   8 17  2,   3 18  5,
                0 18  6,  15 10  6,  17  6  6,   5 17  7,   3 17  8,  19  0 10,
               15 12 14,   2 12 16,   4 11 16,   7  3 19,
PLAT918_ALERT_3_C Reflection(s) with I(obs) much Smaller I(calc) .          1 Check
```

Alert level G

```
FORMU01_ALERT_2_G  There is a discrepancy between the atom counts in the
            _chemical_formula_sum and the formula from the _atom_site* data.
            Atom count from _chemical_formula_sum:C31.8 H28.2 Cu2 N6.6 Ni6 O0.6 S9
            Atom count from the _atom_site data:  C30 H24 Cu2 N6 Ni6 S9
ABSMU01_ALERT_1_G  Calculation of _exptl_absorpt_correction_mu
                not performed for this radiation type.
CELLZ01_ALERT_1_G Difference between formula and atom_site contents detected.
CELLZ01_ALERT_1_G ALERT: Large difference may be due to a
            symmetry error - see SYMMG tests
           From the CIF: _cell_formula_units_Z    8
           From the CIF: _chemical_formula_sum  C31.80 H28.20 Cu2 N6.60 Ni6 O0.60
           TEST: Compare cell contents of formula and atom_site data

           atom    Z*formula  cif sites diff
           C        254.40    240.00   14.40
           H        225.60    192.00   33.60
           Cu        16.00     16.00    0.00
           N         52.80     48.00    4.80
           Ni        48.00     48.00    0.00
           O          4.80      0.00    4.80
           S         72.00     72.00    0.00
PLAT004_ALERT_5_G Polymeric Structure Found with Maximum Dimension          1 Info
PLAT041_ALERT_1_G Calc. and Reported SumFormula    Strings  Differ     Please Check
              Calc: C30 H24 Cu2 N6 Ni6 S9
              Rep.: C31.80 H28.20 Cu2 N6.60 Ni6 O0.60 S9
PLAT042_ALERT_1_G Calc. and Reported MoietyFormula Strings  Differ     Please Check
              Calc: C30 H24 Cu2 N6 Ni6 S9
              Rep.: C30 H24 Cu2 N6 Ni6 S9, 0.6[C3H7NO]
PLAT066_ALERT_1_G Predicted and Reported Tmin&Tmax Range Identical          ? Check
PLAT232_ALERT_2_G Hirshfeld Test Diff (M-X)  Cu07     --S00G_a    .        9.3 s.u.
PLAT232_ALERT_2_G Hirshfeld Test Diff (M-X)  Cu08     --S00E_b    .       15.0 s.u.
```

```
PLAT232_ALERT_2_G Hirshfeld Test Diff (M-X)  Ni05     --S00F     .        5.2 s.u.
PLAT605_ALERT_4_G Largest Solvent Accessible VOID in the Structure        203 A**3
PLAT720_ALERT_4_G Number of Unusual/Non-Standard Labels ..........         77 Note
              Ni01    Ni02    Ni03    Ni04    Ni05    Ni06    Cu07    Cu08
              S009    S00A    S00B    S00C    S00D    S00E    S00F    S00G
              S00H    N00I    N00J    N00K    N00L    N00M    N00N    C00O
              C00P    H00P    C00Q    C00R    H00R    C00S    H00S    C00T
              H00T    C00U    H00U    C00V    C00W    C00X    H00X    C00Y
              H00Y    C00Z    C010    H010    C011    H011    C012    H012
              C013    C014    H014    C015    H015    C016    H016    C017
              H017    C018    H018    C019    H019    C01A    H01A    C01B
              H01B    C01C    H01C    C01D    H01D    C01E    H01E    C01F
              H01F    C01G    H01G    C01H    H01H
PLAT764_ALERT_4_G Overcomplete CIF Bond List Detected (Rep/Expd) .       1.20 Ratio
PLAT794_ALERT_5_G Tentative Bond Valency for Ni01      (II)      .       1.97 Info
PLAT794_ALERT_5_G Tentative Bond Valency for Ni02      (II)      .       2.09 Info
PLAT794_ALERT_5_G Tentative Bond Valency for Ni03      (II)      .       2.02 Info
PLAT794_ALERT_5_G Tentative Bond Valency for Ni04      (II)      .       1.99 Info
PLAT794_ALERT_5_G Tentative Bond Valency for Ni05      (II)      .       2.00 Info
PLAT794_ALERT_5_G Tentative Bond Valency for Ni06      (II)      .       1.96 Info
PLAT868_ALERT_4_G ALERTS Due to the Use of _smtbx_masks Suppressed          ! Info
PLAT912_ALERT_4_G Missing # of FCF Reflections Above STh/L=  0.600        416 Note
PLAT941_ALERT_3_G Average HKL Measurement Multiplicity ...........        4.3 Low
PLAT969_ALERT_5_G The 'Henn et al.' R-Factor-gap value ...........      3.005 Note
              Predicted wR2: Based on SigI**2  2.73 or SHELX Weight  9.02
PLAT978_ALERT_2_G Number C-C Bonds with Positive Residual Density.          0 Info
```

---

0 **ALERT level A** = Most likely a serious problem - resolve or explain
0 **ALERT level B** = A potentially serious problem, consider carefully
5 **ALERT level C** = Check. Ensure it is not caused by an omission or oversight
25 **ALERT level G** = General information/check it is not something unexpected

7 ALERT type 1 CIF construction/syntax error, inconsistent or missing data
5 ALERT type 2 Indicator that the structure model may be wrong or deficient
4 ALERT type 3 Indicator that the structure quality may be low
6 ALERT type 4 Improvement, methodology, query or suggestion
8 ALERT type 5 Informative message, check

It is advisable to attempt to resolve as many as possible of the alerts in all categories. Often the minor alerts point to easily fixed oversights, errors and omissions in your CIF or refinement strategy, so attention to these fine details can be worthwhile. In order to resolve some of the more serious problems it may be necessary to carry out additional measurements or structure refinements. However, the purpose of your study may justify the reported deviations and the more serious of these should normally be commented upon in the discussion or experimental section of a paper or in the "special_details" fields of the CIF. checkCIF was carefully designed to identify outliers and unusual parameters, but every test has its limitations and alerts that are not important in a particular case may appear. Conversely, the absence of alerts does not guarantee there are no aspects of the results needing attention. It is up to the individual to critically assess their own results and, if necessary, seek expert advice.

Publication of your CIF in IUCr journals

A basic structural check has been run on your CIF. These basic checks will be run on all CIFs submitted for publication in IUCr journals (*Acta Crystallographica*, *Journal of Applied Crystallography*, *Journal of Synchrotron Radiation*); however, if you intend to submit to *Acta Crystallographica Section C* or *E* or *IUCrData*, you should make sure that full publication checks are run on the final version of your CIF prior to submission.

Publication of your CIF in other journals

Please refer to the *Notes for Authors* of the relevant journal for any special instructions relating to CIF submission.

PLATON version of 02/02/2025; check.def file version of 02/02/2025

Datablock 1 - ellipsoid plot

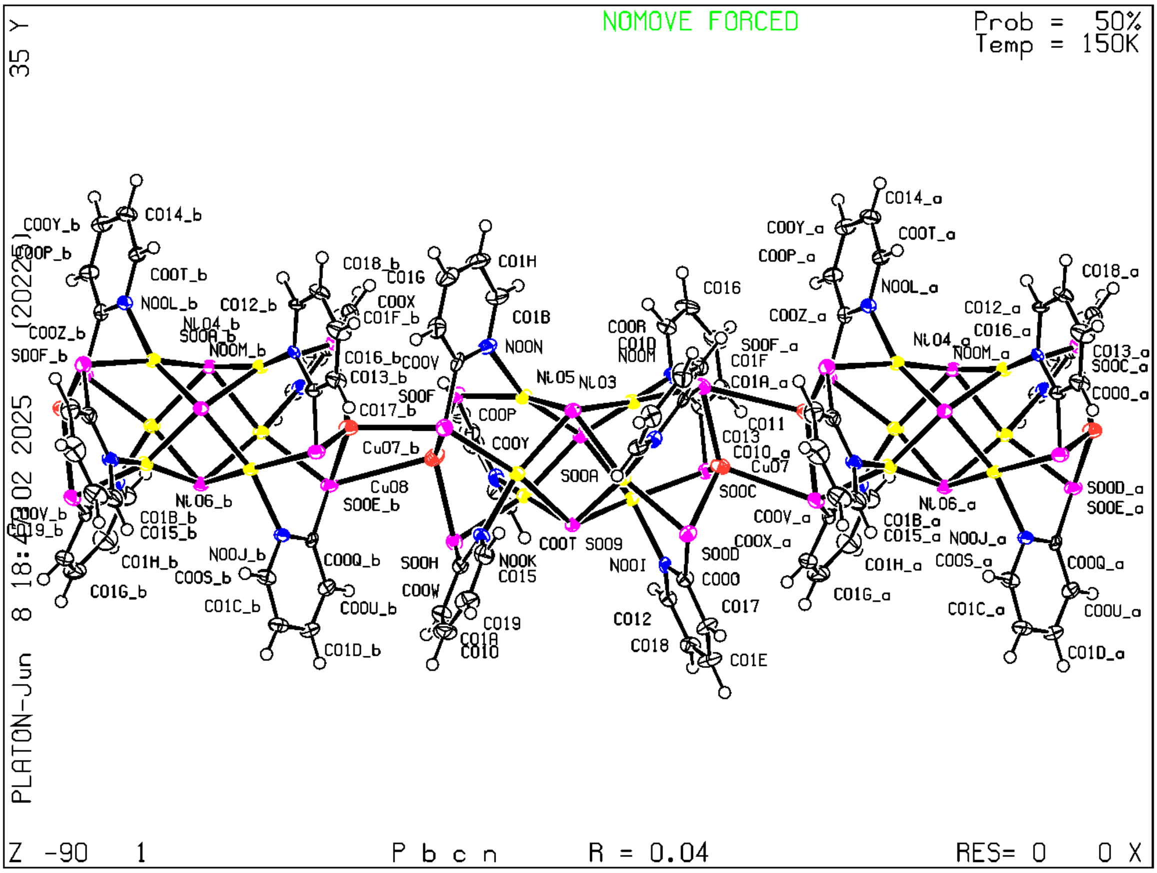

Supplement: Supplementary file 2 — Supplemental cif [file SMLL-21-e06920-s002.zip › 1-checkcif.pdf]
