# Supplementary material for: Bonding Features and Magnetic Ordering in Thiolate‐Bridged Copper‐Nickel Clusters Synthesized at Elevated Temperature
Source: Small. 2025 Aug 8;21(38):e06920. doi: 10.1002/smll.202506920 (PMC12462566; doi:10.1002/smll.202506920)

checkCIF/PLATON report

Structure factors have been supplied for datablock(s) nipyt9blackcuboid

THIS REPORT IS FOR GUIDANCE ONLY. IF USED AS PART OF A REVIEW PROCEDURE FOR PUBLICATION, IT SHOULD NOT REPLACE THE EXPERTISE OF AN EXPERIENCED CRYSTALLOGRAPHIC REFEREE.

No syntax errors found. CIF dictionary Interpreting this report

Datablock: nipyt9blackcuboid

Bond precision: C-C = 0.0138 A Wavelength=1.34143

Cell:	a=12.3680(5)	b=13.3888(5)	c=13.7219(4)
	alpha=115.397(2)	beta=97.655(3)	gamma=104.303(3)
Temperature:	150 K		

	Calculated	Reported
Volume	1913.35(14)	1913.34(13)
Space group	P -1	P -1
Hall group	-P 1	-P 1
Moiety formula	C60 H48 Cu2 I2 N12 Ni6 S12 [+ solvent]	C60 H48 Cu2 I2 N12 Ni6 S12, 0.6[C3H7NO]
Sum formula	C60 H48 Cu2 I2 N12 Ni6 S12 [+ solvent]	C61.80 H52.20 Br2 Cl2 Cu2 I2 N12.60 Ni6 O0.60 S12
Mr	2054.86	2329.54
Dx,g cm-3	1.783	2.022
Z	1	1
Mu (mm-1)	17.465	18.766
F000	1016.0	1144.0
F000'	1007.75	
h,k,lmax	16,17,18	16,17,18
Nref	9444	9171
Tmin,Tmax	0.158,0.153	0.153,0.174
Tmin'	0.070	

Correction method= # Reported T Limits: Tmin=0.153 Tmax=0.174
AbsCorr = MULTI-SCAN

Data completeness= 0.971 Theta(max)= 63.163

R(reflections)= 0.0625(5600) wR2(reflections)= 0.1879(9171)

S = 0.962 Npar= 424

The following ALERTS were generated. Each ALERT has the format
test-name_ALERT_alert-type_alert-level.
Click on the hyperlinks for more details of the test.

Alert level B

```
PLAT972_ALERT_2_B Check Calcd Resid. Dens.  0.58Ang From I3           -2.60 eA-3
```

Author Response: This is due to a slightly limited quality of the crystal and the diffraction data.

Alert level C

```
ABSTY02_ALERT_1_C  An _exptl_absorpt_correction_type has been given without
            a literature citation. This should be contained in the
            _exptl_absorpt_process_details field.
            Absorption correction given as multi-scan
PLAT213_ALERT_2_C Atom C012           has ADP max/min Ratio .....        3.2 prolat
PLAT213_ALERT_2_C Atom C01B           has ADP max/min Ratio .....        3.1 prolat
PLAT220_ALERT_2_C NonSolvent   Resd 1  C   Ueq(max)/Ueq(min) Range       3.8 Ratio
PLAT241_ALERT_2_C High   'MainMol' Ueq as Compared to Neighbors of      C01B Check
PLAT342_ALERT_3_C Low Bond Precision on  C-C Bonds ..............    0.01379 Ang.
PLAT767_ALERT_4_C INS Embedded LIST 6 Instruction Should be LIST 4     Please Check
PLAT911_ALERT_3_C Missing FCF Refl Between Thmin & STh/L=    0.600         49 Report
                 0  1  0, -12  2  0,   0  2  0,   0  3  0,   0 -3  1,   2 -2  1,
                -3 -1  1,   2 -1  1,   3 -1  1,  -2  0  1,  -1  0  1,  -3  1  1,
                 0  1  1,   0 -6  2,  -1 -1  2,  -3  0  2,  -2  0  2,   1  0  2,
                 4-13  3,  -2 -1  3,  -3  0  3,  -3  1  3,  -5 -2  4,   5  6  4,
                 0  9  4,   5-13  5,  -2  8  6,   2-13  8,   5  2  8,   4  3  8,
                 8 -5  9,   5 -1 10,   3  1 10,  -2-11 11,  -9 -7 11,   6 -6 11,
                -1  2 11,  -7 -4 12,   4 -4 12,  -7 -1 12,   2 -5 13,   1 -3 13,
                -3 -2 13,  -2 -2 13,  -1 -2 13,   3 -4 14,   4 -4 14,   4 -3 14,
                 3 -5 15,
PLAT972_ALERT_2_C Check Calcd Resid. Dens.  0.69Ang From I3           -2.50 eA-3
```

Author Response: This is due to a slightly limited quality of the crystal and the diffraction data.

```
PLAT972_ALERT_2_C Check Calcd Resid. Dens.  0.52Ang From I3           -2.48 eA-3
```

Author Response: This is due to a slightly limited quality of the crystal and the diffraction data.

```
PLAT972_ALERT_2_C Check Calcd Resid. Dens.  0.50Ang From I3            -2.30 eA-3
```

Author Response: This is due to a slightly limited quality of the crystal and the diffraction data.

```
PLAT973_ALERT_2_C Check Calcd Positive Resid. Density on       Ni1       1.41 eA-3
```

Alert level G

```
FORMU01_ALERT_1_G  There is a discrepancy between the atom counts in the
            _chemical_formula_sum and _chemical_formula_moiety. This is
            usually due to the moiety formula being in the wrong format.
            Atom count from _chemical_formula_sum:   C61.8 H52.2 Br2 Cl2 Cu2 I2 N1
            Atom count from _chemical_formula_moiety:C61.8 H52.2 Cu2 I2 N12.6 Ni6
FORMU01_ALERT_2_G  There is a discrepancy between the atom counts in the
            _chemical_formula_sum and the formula from the _atom_site* data.
            Atom count from _chemical_formula_sum:C61.8 H52.2 Br2 Cl2 Cu2 I2 N12.6
            Atom count from the _atom_site data:  C60 H48 Cu2 I2 N12 Ni6 S12
ABSMU01_ALERT_1_G  Calculation of _exptl_absorpt_correction_mu
                not performed for this radiation type.
CELLZ01_ALERT_1_G Difference between formula and atom_site contents detected.
CELLZ01_ALERT_1_G ALERT: Large difference may be due to a
            symmetry error - see SYMMG tests
           From the CIF: _cell_formula_units_Z    1
           From the CIF: _chemical_formula_sum  C61.80 H52.20 Br2 Cl2 Cu2 I2 N12.6
           TEST: Compare cell contents of formula and atom_site data

           atom    Z*formula  cif sites diff
           C         61.80     60.00    1.80
           H         52.20     48.00    4.20
           Br         2.00      0.00    2.00
           Cl         2.00      0.00    2.00
           Cu         2.00      2.00    0.00
           I          2.00      2.00    0.00
           N         12.60     12.00    0.60
           Ni         6.00      6.00    0.00
           O          0.60      0.00    0.60
           S         12.00     12.00    0.00
PLAT041_ALERT_1_G Calc. and Reported SumFormula    Strings  Differ     Please Check
              Calc: C60 H48 Cu2 I2 N12 Ni6 S12
              Rep.: C61.80 H52.20 Br2 Cl2 Cu2 I2 N12.60 Ni6 O0.60
                    S12
PLAT042_ALERT_1_G Calc. and Reported MoietyFormula Strings  Differ     Please Check
              Calc: C60 H48 Cu2 I2 N12 Ni6 S12
              Rep.: C60 H48 Cu2 I2 N12 Ni6 S12, 0.6[C3H7NO]
PLAT051_ALERT_1_G Mu(calc) and Mu(cif) Ratio Differs from 1.0 by .       6.93 %
PLAT072_ALERT_2_G SHELXL First  Parameter in WGHT  Unusually Large       0.11 Report
PLAT232_ALERT_2_G Hirshfeld Test Diff (M-X)  I3       --Cu3      .       25.8 s.u.
PLAT232_ALERT_2_G Hirshfeld Test Diff (M-X)  Cu3      --S006     .        5.1 s.u.
PLAT232_ALERT_2_G Hirshfeld Test Diff (M-X)  Ni1      --S006     .        5.1 s.u.
PLAT605_ALERT_4_G Largest Solvent Accessible VOID in the Structure        211 A**3
PLAT720_ALERT_4_G Number of Unusual/Non-Standard Labels ..........         67 Note
              Ni02    S006    S007    S008    S009    S00A    S00B    N00C
              N00D    N00E    N00F    N00G    N00H    C00I    C00J    C00K
              C00L    C00M    H00M    C00N    H00N    C00O    H00O    C00P
```

```
              H00P    C00Q    H00Q    C00R    H00R    C00S    H00S    C00T
              C00U    H00U    C00V    C00W    H00W    C00X    H00X    C00Y
              H00Y    C00Z    H00Z    C010    H010    C011    H011    C012
              H012    C013    H013    C014    H014    C015    H015    C016
              H016    C017    H017    C018    H018    C019    H019    C01A
              H01A    C01B    H01B
PLAT794_ALERT_5_G Tentative Bond Valency for Ni1       (II)      .       1.90 Info
PLAT794_ALERT_5_G Tentative Bond Valency for Ni02      (II)      .       1.84 Info
PLAT794_ALERT_5_G Tentative Bond Valency for Ni2       (II)      .       1.91 Info
PLAT868_ALERT_4_G ALERTS Due to the Use of _smtbx_masks Suppressed          ! Info
PLAT883_ALERT_1_G Absent Datum for _atom_sites_solution_primary ..     Please Do !
PLAT912_ALERT_4_G Missing # of FCF Reflections Above STh/L=  0.600        224 Note
PLAT933_ALERT_2_G Number of HKL-OMIT Records in Embedded .res File          4 Note
               -3 -1  1,  -3  1  1,   1  0  2,   2 -2  1,
PLAT941_ALERT_3_G Average HKL Measurement Multiplicity ...........        4.0 Low
PLAT969_ALERT_5_G The 'Henn et al.' R-Factor-gap value ...........      3.850 Note
              Predicted wR2: Based on SigI**2  4.88 or SHELX Weight 19.53
PLAT978_ALERT_2_G Number C-C Bonds with Positive Residual Density.          1 Info
```

---

  0 **ALERT level A** = Most likely a serious problem - resolve or explain
  1 **ALERT level B** = A potentially serious problem, consider carefully
 12 **ALERT level C** = Check. Ensure it is not caused by an omission or oversight
 24 **ALERT level G** = General information/check it is not something unexpected

  9 ALERT type 1 CIF construction/syntax error, inconsistent or missing data
 16 ALERT type 2 Indicator that the structure model may be wrong or deficient
  3 ALERT type 3 Indicator that the structure quality may be low
  5 ALERT type 4 Improvement, methodology, query or suggestion
  4 ALERT type 5 Informative message, check

It is advisable to attempt to resolve as many as possible of the alerts in all categories. Often the minor alerts point to easily fixed oversights, errors and omissions in your CIF or refinement strategy, so attention to these fine details can be worthwhile. In order to resolve some of the more serious problems it may be necessary to carry out additional measurements or structure refinements. However, the purpose of your study may justify the reported deviations and the more serious of these should normally be commented upon in the discussion or experimental section of a paper or in the "special_details" fields of the CIF. checkCIF was carefully designed to identify outliers and unusual parameters, but every test has its limitations and alerts that are not important in a particular case may appear. Conversely, the absence of alerts does not guarantee there are no aspects of the results needing attention. It is up to the individual to critically assess their own results and, if necessary, seek expert advice.

Publication of your CIF in IUCr journals

A basic structural check has been run on your CIF. These basic checks will be run on all CIFs submitted for publication in IUCr journals (*Acta Crystallographica*, *Journal of Applied Crystallography*, *Journal of Synchrotron Radiation*); however, if you intend to submit to *Acta Crystallographica Section C* or *E* or *IUCrData*, you should make sure that full publication checks are run on the final version of your CIF prior to submission.

Publication of your CIF in other journals

Please refer to the *Notes for Authors* of the relevant journal for any special instructions relating to CIF submission.

PLATON version of 02/02/2025; check.def file version of 02/02/2025

Datablock nipyt9blackcuboid - ellipsoid plot

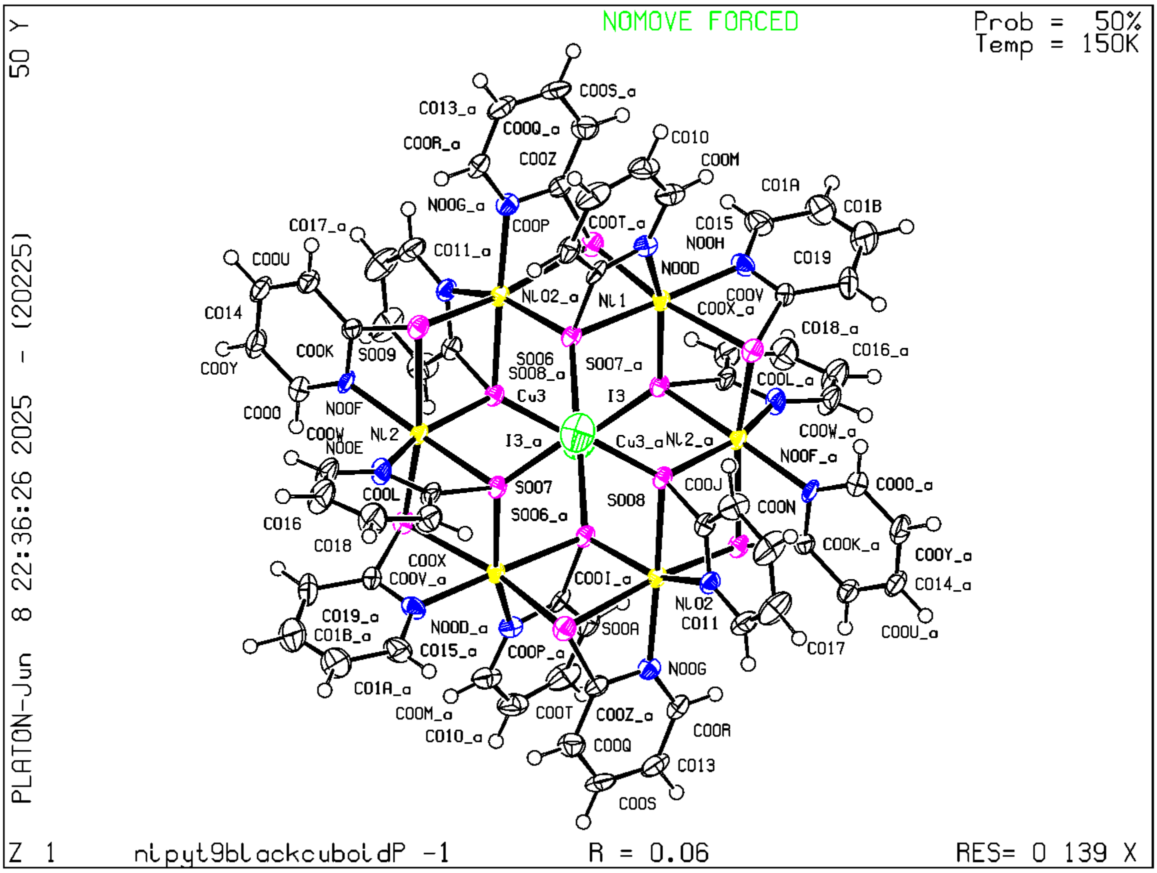

Supplement: Supplementary file 2 — Supplemental cif [file SMLL-21-e06920-s002.zip › 2a-checkcif.pdf]
