# Supplementary material for: Bonding Features and Magnetic Ordering in Thiolate‐Bridged Copper‐Nickel Clusters Synthesized at Elevated Temperature
Source: Small. 2025 Aug 8;21(38):e06920. doi: 10.1002/smll.202506920 (PMC12462566; doi:10.1002/smll.202506920)

checkCIF/PLATON report

Structure factors have been supplied for datablock(s) 2b

THIS REPORT IS FOR GUIDANCE ONLY. IF USED AS PART OF A REVIEW PROCEDURE FOR PUBLICATION, IT SHOULD NOT REPLACE THE EXPERTISE OF AN EXPERIENCED CRYSTALLOGRAPHIC REFEREE.

No syntax errors found. CIF dictionary Interpreting this report

Datablock: 2b

```
Bond precision:    C-C = 0.0091 A           Wavelength=1.34143

Cell:              a=13.8178(14)      b=12.4690(16)       c=24.384(3)
                   alpha=90           beta=98.714(9)      gamma=90
Temperature:       150 K
```

	Calculated	Reported
Volume	4152.7(9)	4152.7(8)
Space group	P 21/c	P 1 21/c 1
Hall group	-P 2ybc	-P 2ybc
Moiety formula	C60 H48 Cu2 I2 N12 Ni6 S12, 2(C3 H7 N O) [+ solvent]	C60 H48 Cu2 I2 N12 Ni6 S12, 2(C3 H7 N O), 0.6[C3H7NO]
Sum formula	C66 H62 Cu2 I2 N14 Ni6 O2 S12 [+ solvent]	C67.80 H66.20 Cu2 I2 N14.60 Ni6 O2.60 S12
Mr	2201.06	2245.01
Dx,g cm-3	1.760	1.795
Z	2	2
Mu (mm-1)	16.140	16.150
F000	2192.0	2240.0
F000'	2175.75	
h,k,lmax	18,16,32	17,16,32
Nref	9917	9576
Tmin,Tmax	0.169,0.144	0.144,0.169
Tmin'	0.047	

```
Correction method= # Reported T Limits: Tmin=0.144 Tmax=0.169
AbsCorr = MULTI-SCAN

Data completeness= 0.966           Theta(max)= 61.980
```

R(reflections)= 0.0540(6783)   wR2(reflections)= 0.1529(9576)

S = 1.048   Npar= 471

The following ALERTS were generated. Each ALERT has the format
test-name_ALERT_alert-type_alert-level.
Click on the hyperlinks for more details of the test.

Alert level C

```
ABSTY02_ALERT_1_C  An _exptl_absorpt_correction_type has been given without
            a literature citation. This should be contained in the
            _exptl_absorpt_process_details field.
            Absorption correction given as multi-scan
PLAT244_ALERT_4_C Low    'Solvent' Ueq as Compared to Neighbors of       N011 Check
PLAT260_ALERT_2_C Large Average Ueq of Residue Including      O019      0.102 Check
PLAT342_ALERT_3_C Low Bond Precision on  C-C Bonds ...............    0.00912 Ang.
PLAT767_ALERT_4_C INS Embedded LIST 6 Instruction Should be LIST 4     Please Check
PLAT911_ALERT_3_C Missing FCF Refl Between Thmin & STh/L=    0.600          83 Report
               -2 14  1,   3 10  1,   5 11  1,  13  2  1, -12  5  2,  -2  1  2,
               -2 12  2,  -1  1  2,   0  0  2,   1  0  2,   2 12  2,   5 11  2,
              -12  5  3,  -2 12  3,  -1  1  3,  -1  2  3,  -1 12  3,   0 12  3,
                1 12  3,   5 11  3,  -1  0  4,  -1  2  4,  -1  3  4,  -1 12  4,
                0 12  4,   1 12  4,   2  0  4,  -2 13  5,   1 12  5, -16  0  6,
                3  0  6,   3  1  6,  12  2  6, -16  1  7,  -4 12  7, -16  0  8,
              -16  1  8, -15  0  8, -16  1  9,  -8  9  9,  -3 11  9,  -2 11  9,
                0  2  9,   3 13  9, -16  0 10, -16  1 10, -15  0 10, -13  0 10,
               -2  0 10,  -2 11 10,   0 11 10,   3 13 10,   5  0 10,  11  2 10,
              -16  1 11,   0  1 11,   7  8 11,   7  8 12,  10  3 12,  -2 10 13,
                9  4 13,  10  1 13, -12  0 14,   6  7 14,   9  1 15, -15  0 16,
               -6  6 19,  -4  7 19,  -3  7 19,  -2  7 19,   8  5 19,   3  5 20,
                8  5 20,  -6  4 21,   4  2 21,   8  5 21,  -6  2 22,  -4  4 22,
                2  3 22,   3  0 22,   3  1 22,  -5  1 23,   7  0 24,
PLAT918_ALERT_3_C Reflection(s) with I(obs) much Smaller I(calc) .          3 Check
PLAT972_ALERT_2_C Check Calcd Resid. Dens.  0.67Ang From I              -1.56 eA-3
PLAT972_ALERT_2_C Check Calcd Resid. Dens.  0.57Ang From I              -1.53 eA-3
```

Alert level G

```
FORMU01_ALERT_2_G  There is a discrepancy between the atom counts in the
            _chemical_formula_sum and the formula from the _atom_site* data.
            Atom count from _chemical_formula_sum:C67.8 H66.2 Cu2 I2 N14.6 Ni6 O2.
            Atom count from the _atom_site data:  C66 H62 Cu2 I2 N14 Ni6 O2 S12
ABSMU01_ALERT_1_G  Calculation of _exptl_absorpt_correction_mu
                not performed for this radiation type.
CELLZ01_ALERT_1_G Difference between formula and atom_site contents detected.
CELLZ01_ALERT_1_G ALERT: Large difference may be due to a
            symmetry error - see SYMMG tests
           From the CIF: _cell_formula_units_Z    2
           From the CIF: _chemical_formula_sum  C67.80 H66.20 Cu2 I2 N14.60 Ni6 O2
           TEST: Compare cell contents of formula and atom_site data

           atom    Z*formula  cif sites diff
           C        135.60    132.00    3.60
           H        132.40    124.00    8.40
```

```
          Cu         4.00      4.00    0.00
          I          4.00      4.00    0.00
          N         29.20     28.00    1.20
          Ni        12.00     12.00    0.00
          O          5.20      4.00    1.20
          S         24.00     24.00    0.00
PLAT041_ALERT_1_G Calc. and Reported SumFormula    Strings  Differ     Please Check
             Calc: C66 H62 Cu2 I2 N14 Ni6 O2 S12
             Rep.: C67.80 H66.20 Cu2 I2 N14.60 Ni6 O2.60 S12
PLAT042_ALERT_1_G Calc. and Reported MoietyFormula Strings  Differ     Please Check
             Calc: C60 H48 Cu2 I2 N12 Ni6 S12, 2(C3 H7 N O)
             Rep.: C60 H48 Cu2 I2 N12 Ni6 S12, 2(C3 H7 N O), 0.6[
                   C3H7NO]
PLAT232_ALERT_2_G Hirshfeld Test Diff (M-X)  I        --Cu05     .       15.5 s.u.
PLAT232_ALERT_2_G Hirshfeld Test Diff (M-X)  Cu05     --S007     .        5.7 s.u.
PLAT232_ALERT_2_G Hirshfeld Test Diff (M-X)  Cu05     --S008     .        6.9 s.u.
PLAT232_ALERT_2_G Hirshfeld Test Diff (M-X)  Ni04     --S007     .        7.1 s.u.
PLAT380_ALERT_4_G Incorrectly? Oriented X(sp2)-Methyl Moiety .....       C01F Check
PLAT432_ALERT_2_G Short Inter X...Y Contact  C01F     ..C01F     .       3.17 Ang.
                                                 2-x,-y,1-z  =      3_756 Check
PLAT605_ALERT_4_G Largest Solvent Accessible VOID in the Structure         64 A**3
PLAT720_ALERT_4_G Number of Unusual/Non-Standard Labels ..........         82 Note
             Ni02    Ni03    Ni04    Cu05    S006    S007    S008    S009
             S00A    S00B    N00C    N00D    N00E    N00F    N00G    N00H
             C00I    C00J    C00K    H00K    C00L    H00L    C00M    H00M
             C00N    H00N    C00O    C00P    H00P    C00Q    C00R    C00S
             H00S    C00T    C00U    H00U    C00V    H00V    C00W    H00W
             C00X    H00X    C00Y    H00Y    C00Z    H00Z    C010    H010
             N011    C012    H012    C013    H013    C014    H014    C015
             H015    C016    H016    C017    H017    C018    H018    O019
             C01A    H01A    C01B    H01B    C01C    H01C    C01D    H01D
             C01E    H01E    H01F    H01G    C01F    H01H    H01I    H01J
             C01G    H01K
PLAT794_ALERT_5_G Tentative Bond Valency for Ni02      (II)      .       1.86 Info
PLAT794_ALERT_5_G Tentative Bond Valency for Ni03      (II)      .       1.87 Info
PLAT794_ALERT_5_G Tentative Bond Valency for Ni04      (II)      .       1.85 Info
PLAT868_ALERT_4_G ALERTS Due to the Use of _smtbx_masks Suppressed          ! Info
PLAT912_ALERT_4_G Missing # of FCF Reflections Above STh/L=  0.600        241 Note
PLAT933_ALERT_2_G Number of HKL-OMIT Records in Embedded .res File          2 Note
              -2  1  2,   1  0  2,
PLAT941_ALERT_3_G Average HKL Measurement Multiplicity ..........         3.9 Low
PLAT969_ALERT_5_G The 'Henn et al.' R-Factor-gap value ..........       6.369 Note
             Predicted wR2: Based on SigI**2  2.40 or SHELX Weight 14.59
PLAT978_ALERT_2_G Number C-C Bonds with Positive Residual Density.          0 Info
PLAT984_ALERT_1_G The Cu-f'=   -2.9183 Deviates from the B&C-Value    -2.7974 Check
PLAT985_ALERT_1_G The Cu-f"=    3.6937 Deviates from the B&C-Value     3.6876 Check
```

---

   0 **ALERT level A** = Most likely a serious problem - resolve or explain
   0 **ALERT level B** = A potentially serious problem, consider carefully
   9 **ALERT level C** = Check. Ensure it is not caused by an omission or oversight
  25 **ALERT level G** = General information/check it is not something unexpected

   8 ALERT type 1 CIF construction/syntax error, inconsistent or missing data

```
11 ALERT type 2 Indicator that the structure model may be wrong or deficient
 4 ALERT type 3 Indicator that the structure quality may be low
 7 ALERT type 4 Improvement, methodology, query or suggestion
 4 ALERT type 5 Informative message, check
```

It is advisable to attempt to resolve as many as possible of the alerts in all categories. Often the minor alerts point to easily fixed oversights, errors and omissions in your CIF or refinement strategy, so attention to these fine details can be worthwhile. In order to resolve some of the more serious problems it may be necessary to carry out additional measurements or structure refinements. However, the purpose of your study may justify the reported deviations and the more serious of these should normally be commented upon in the discussion or experimental section of a paper or in the "special_details" fields of the CIF. checkCIF was carefully designed to identify outliers and unusual parameters, but every test has its limitations and alerts that are not important in a particular case may appear. Conversely, the absence of alerts does not guarantee there are no aspects of the results needing attention. It is up to the individual to critically assess their own results and, if necessary, seek expert advice.

Publication of your CIF in IUCr journals

A basic structural check has been run on your CIF. These basic checks will be run on all CIFs submitted for publication in IUCr journals (*Acta Crystallographica*, *Journal of Applied Crystallography*, *Journal of Synchrotron Radiation*); however, if you intend to submit to *Acta Crystallographica Section C* or *E* or *IUCrData*, you should make sure that full publication checks are run on the final version of your CIF prior to submission.

Publication of your CIF in other journals

Please refer to the *Notes for Authors* of the relevant journal for any special instructions relating to CIF submission.

PLATON version of 02/02/2025; check.def file version of 02/02/2025

Datablock 2b - ellipsoid plot

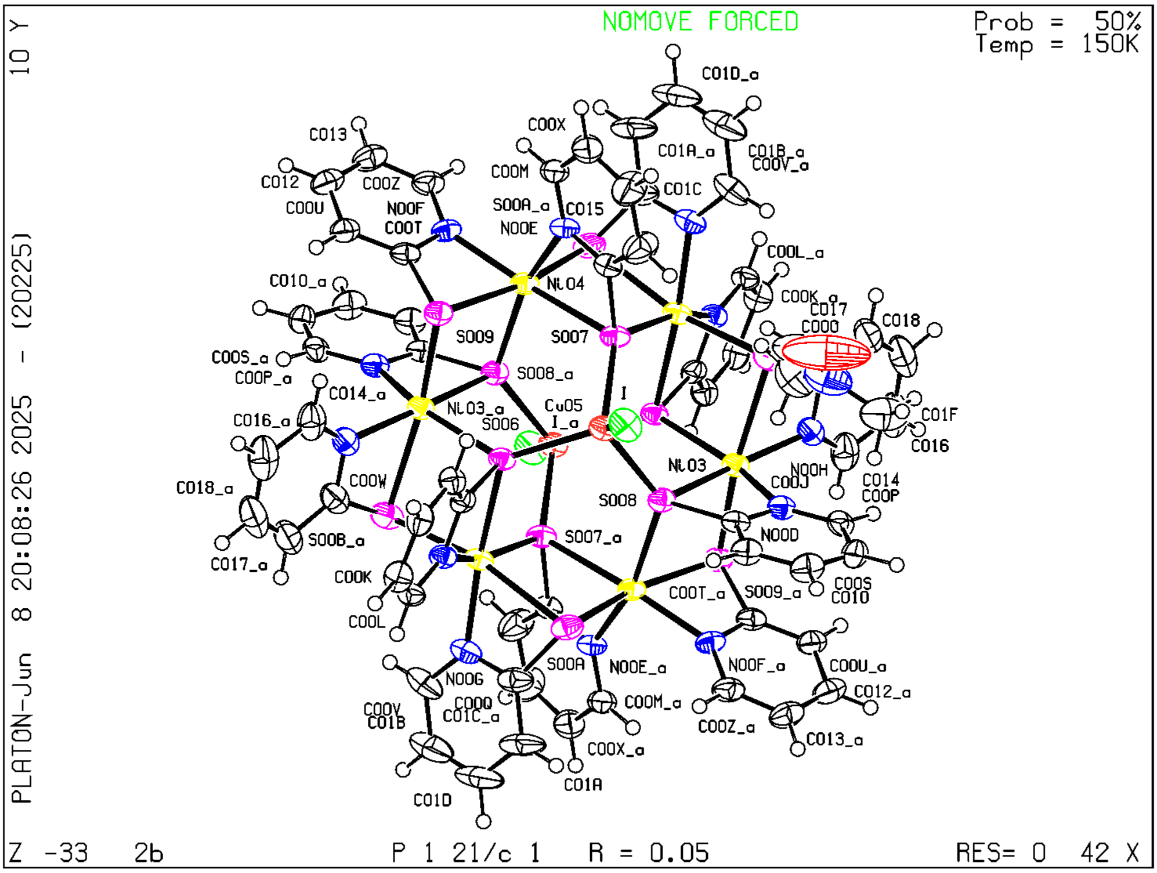

Supplement: Supplementary file 2 — Supplemental cif [file SMLL-21-e06920-s002.zip › 2b-checkcif.pdf]
